# Pathways to defense metabolites and evading fruit bitterness in genus *Solanum* evolved through 2-oxoglutarate-dependent dioxygenases

**DOI:** 10.1038/s41467-019-13211-4

**Published:** 2019-11-14

**Authors:** Pablo D. Cárdenas, Prashant D. Sonawane, Uwe Heinig, Adam Jozwiak, Sayantan Panda, Bekele Abebie, Yana Kazachkova, Margarita Pliner, Tamar Unger, Dalia Wolf, Itai Ofner, Ester Vilaprinyo, Sagit Meir, Olga Davydov, Amit Gal-on, Saul Burdman, Ashok Giri, Dani Zamir, Tali Scherf, Jedrzej Szymanski, Ilana Rogachev, Asaph Aharoni

**Affiliations:** 1 Department of Plant and Environmental Sciences, Weizmann Institute of Science, Rehovot, 7610001 Israel; 2 Department of Plant Pathology and Microbiology and The Otto Warburg Minerva Center for Agricultural Biotechnology, The Robert H. Smith Faculty of Agriculture, Food and Environment, The Hebrew University of Jerusalem, Rehovot, 7610001 Israel; 3 Department of Plant Pathology and Weed Research, Agricultural Research Organization, Volcani Center, Rishon LeZion, 7505101 Israel; 4 Israel Structural Proteomics Centre, Weizmann Institute of Science, Rehovot, 7610001 Israel; 5 Department of Vegetable Research, Agricultural Research Organization, Volcani Center, Rishon LeZion, 7505101 Israel; 6 The Institute of Plant Sciences and Genetics in Agriculture, The Robert H. Smith Faculty of Agriculture, Food and Environment, The Hebrew University of Jerusalem, Rehovot, 7610001 Israel; 7 Department of Basic Medical Sciences, University of Lleida-IRBLleida, Lleida, Spain; 8 Plant Molecular Biology Unit, Division of Biochemical Sciences, Council of Scientific and Industrial Research-National Chemical Laboratory, Pune, 411008 Maharashtra India; 9 NMR unit, Department of Chemical Research Support, Weizmann Institute of Science, Rehovot, 7610001 Israel; 10 Department of Molecular Genetics, Leibniz Institute of Plant Genetics and Crop Plant Research, OT Gatersleben, Corrensstraße 3, 06466 Seeland, Germany; 11 Present Address: Department of Plant and Environmental Sciences, Faculty of Science, University of Copenhagen, Frederiksberg C, Denmark

**Keywords:** Agricultural genetics, Molecular engineering in plants, Secondary metabolism

## Abstract

The genus *Solanum* comprises three food crops (potato, tomato, and eggplant), which are consumed on daily basis worldwide and also producers of notorious anti-nutritional steroidal glycoalkaloids (SGAs). Hydroxylated SGAs (i.e. leptinines) serve as precursors for leptines that act as defenses against Colorado Potato Beetle (*Leptinotarsa decemlineata* Say), an important pest of potato worldwide. However, SGA hydroxylating enzymes remain unknown. Here, we discover that 2-OXOGLUTARATE-DEPENDENT-DIOXYGENASE (2-ODD) enzymes catalyze SGA-hydroxylation across various *Solanum* species. In contrast to cultivated potato, *Solanum chacoense*, a widespread wild potato species, has evolved a 2-ODD enzyme leading to the formation of leptinines. Furthermore, we find a related 2-ODD in tomato that catalyzes the hydroxylation of the bitter *α*-tomatine to hydroxytomatine, the first committed step in the chemical shift towards downstream ripening-associated non-bitter SGAs (e.g. esculeoside A). This 2-ODD enzyme prevents bitterness in ripe tomato fruit consumed today which otherwise would remain unpleasant in taste and more toxic.

## Abstract (web summary)

Steroidal glycoalkaloids (SGAs) accumulate in *Solanum*, but their hydroxylating enzymes are unknown. Here, the authors report 2-OXOGLUTARATE DEPENDENT DIOXYGENASE enzymes that catalyze the committed hydroxylation steps in the biosynthesis of leptinine insecticidal compounds in wild potato or non-bitter SGAs in cultivated tomato.

## Introduction

**Fig. 1:**
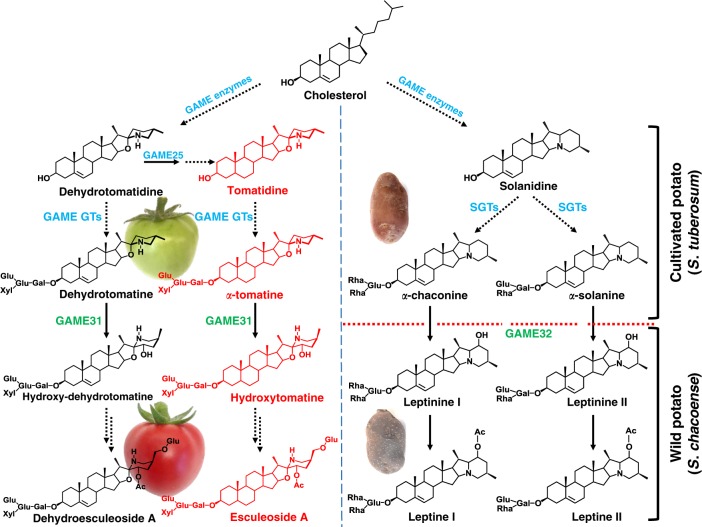
A simplified scheme of core steroidal glycoalkaloid modification in tomato and potato species. In tomato, during transition from green to red fruit, the core *α*-tomatine is bitter steroidal glycoalkaloid (SGA) and converted to the non-bitter esculeoside A and additional SGAs. The main SGAs in cultivated potato are *α*-chaconine and *α*-solanine. Both cultivated potato and *S. chacoense* share a common pathway up to the biosynthesis of *α*-chaconine and *α*-solanine. These SGAs are hydroxylated to form leptinines (leptinine I and leptinine II) and subsequently leptines (leptine I and leptine II) that are unique to the wild potato, *S. chacoense*. The known SGAs biosynthetic genes are shown in blue color. *GAME31* and *GAME32* genes characterized in this study are marked in green. Dashed and solid arrows suggest multiple or single enzymatic steps in the pathway, respectively. A detailed tomato SGAs biosynthetic pathway is presented in Supplementary Fig. 1. GAME GLYCOALKALOID METABOLISM, SGT STEROL ALKALOID GLYCOSYLTRANSFERASE, Ac Acetoxy, Glu Glucose, Gal Galactose, Xyl Xylose, Rha Rhamnose. *α*-tomatine-derived SGAs are marked in red while dehydrotomatine-derived SGAs are shown in black

Steroidal alkaloids and their glycosylated forms (steroidal glycoalkaloids (SGAs)) are specialized metabolites produced mainly in Solanaceae plant species^1–3^. SGAs play a protective role against plant pathogens and predators^2–6^. Yet, some SGAs are considered as anti-nutritional to humans. Their effects in human’s range from high toxicity (e.g. *α*-solanine/*α*-chaconine in potato) to bitter tasting and unpleasant sensations (e.g. *α*-tomatine in tomato)^2–4,7^. In tomato, *α*-tomatine is the main SGA that accumulates in leaves and green fruits^2,7^. During transition from green to red fruit, *α*-tomatine is converted to esculeoside A, a major non-bitter SGA (Fig. 1, and refer Supplementary Fig. 1 for detailed SGA pathway). This chemical shift during fruit ripening involves several modification steps including hydroxylation, acetylation, and glycosylation^8,9^. Hydroxylation of *α*-tomatine to hydroxytomatine is the first proposed step in hitherto uncharacterized esculeoside A biosynthetic pathway in tomato (Fig. 1, and refer Supplementary Fig. 1 for detailed SGA pathway). Therefore, hydroxylation of *α*-tomatine represents the first modification step to reduce its bitterness during tomato fruit ripening.

In cultivated potato (*Solanum tuberosum*), *α*-chaconine and *α*-solanine are the predominant SGAs (Fig. 1), and their biosynthetic route has been elucidated to a large extent^10^. However, in certain wild potato species, such as *Solanum chacoense*, *α*-chaconine/*α*-solanine are hydroxylated to form leptinines (leptinine I and II) which are further acetylated to form leptines (leptine I and II) (Fig. 1). These modified SGAs are known for their insecticidal activity including against the Colorado potato beetle (CPB), a major pest of potato and other *Solanum* crops comprising pepper, tomato, and eggplant^11–18^. New insecticides are desperately desired for CPB control as it lacks natural enemies and developed resistance against most chemicals. Cultivated potato varieties do not produce leptinines and leptines in any of their tissue parts. While several quantitative trait loci (QTL) regions associated with leptinine and leptine biosynthesis were identified in segregating potato populations (*S. tuberosum* × *S. chacoense*), none of the genes partaking in leptinine/leptine biosynthesis have been discovered to date^19–22^. The formation of leptinine I and leptinine II via hydroxylation of *α*-chaconine and *α*-solanine, respectively, is the crucial intermediate step in the leptine biosynthetic pathway.

Notably, biosynthetic enzymes performing hydroxylation step on SGAs remain to be identified in *Solanum* spp. Here we identify 2-oxoglutarate-dependent dioxygenase (2-ODD) family enzymes that catalyze hydroxylation of specific SGA types in cultivated and wild *Solanum* species. The discovery of SGAs hydroxylating enzymes in important crops such as tomato, potato, and eggplant offers the means to reduce anti-nutritional factors, through genetic engineering or breeding programs, alongside protection against major pests and pathogens.

## Results

### Liquid chromatography-mass spectrometry (LC-MS)-based screening of SGAs in tomato introgression lines (ILs)

**Fig. 2:**
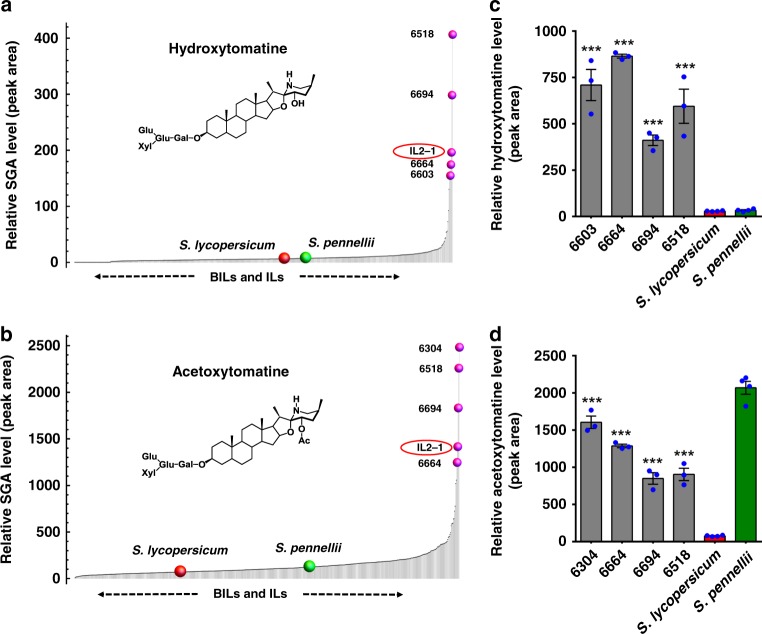
LC-MS-based screening of hydroxytomatine and acetoxytomatine SGAs in the BILs and ILs. **a**, **b** Levels of hydroxytomatine (**a**) and acetoxytomatine (**b**) detected following screening of leaf tissues (*n* = 1, single replicate obtained using the leaf-dipping method) of BIL and IL population (total 671 lines). SGA levels presented in the plots were arranged in ascending order. Top five BILs/ILs showing the highest SGA content are marked by a pink spot. Refer to Supplementary Data 1 for details regarding SGA level across the BIL and IL population. **c**, **d** Validation of hydroxytomatine (**c**) and acetoxytomatine (**d**) content in extracts of ground leaf tissue from selected topmost BILs (from **a**, **b**) along with *S. lycopersicum* (*cv*. M82) and wild species (*S. pennellii*) parents. Values indicate means ± standard error mean (*n* = 4 for parental lines and *n* = 3 for selected BILs and IL2-1 line). Asterisks indicate significant changes compared to *S. lycopersicum* samples as calculated by Student’s *t* test (*P value < 0.05; **P value < 0.01; ***P value < 0.001). LC-MS was used for targeted SGA profiling. The source data of Figs. 2c and 2d are provided as a Source Data file

**Fig. 3:**
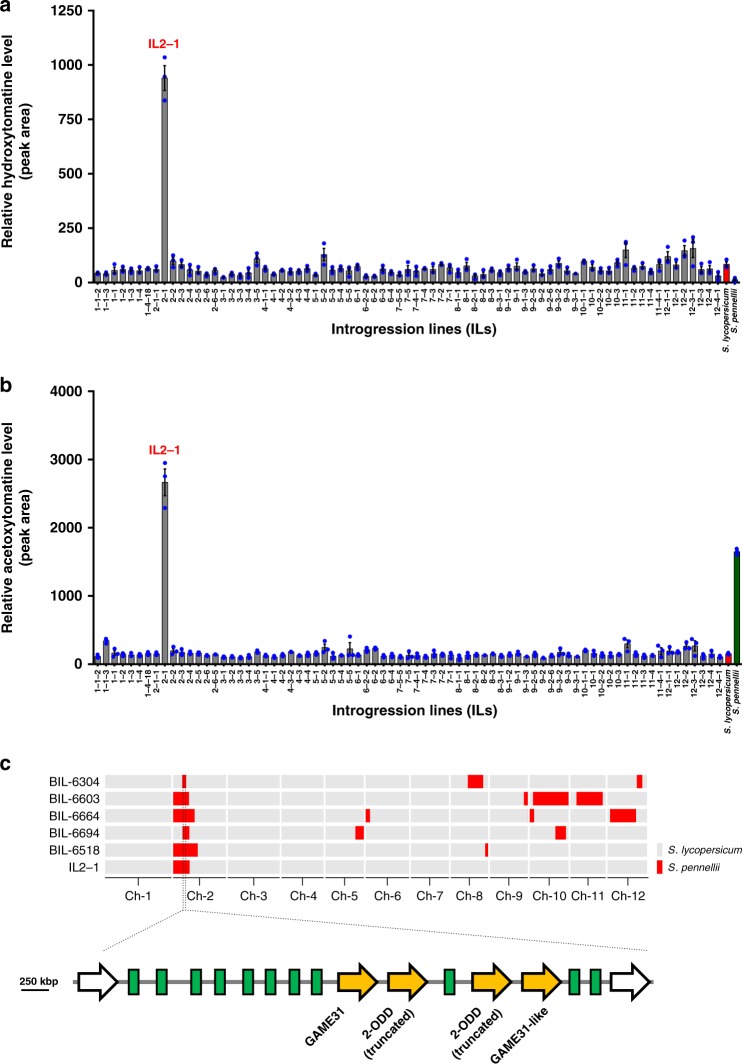
Discovery of the *GAME31* candidate gene in hydroxytomatine-associated QTL region on tomato chromosome 2. **a**, **b** Hydroxytomatine (**a**) and acetoxytomatine (**b**) levels in 77 core IL population including parental lines. SGA content was determined from ground leaf tissue extracts (*n* = 3). The values represent the means of three biological replicates ± standard error mean (*n* = 3). LC-MS was used for targeted SGAs analysis. **c** Schematic representation of chromosomal regions in selected BILs (i.e. #6304, #6603, #6664, #6694, #6518) and the IL2-1 line carrying introgressions from the wild species *S. pennellii*. An overlapping QTL (between selected BILs and IL2-1 line) region located on chromosome 2 affecting the content of hydroxytomatine and acetoxytomatine revealed 17 annotated genes. A putative candidate gene termed *GAME31* belonging to the 2-oxoglutarate dependent dioxygenase (2-ODD) family is depicted with a yellow block arrow along with three other 2-ODDs in the region. The remaining 11 genes are marked in green color, while the first and last genes in the region are shown with a white block arrow. Details regarding these 17 genes are provided in Supplementary Data 2. The source data of Figs. 3a and 3b are provided as a Source Data file

We hypothesized that leptinines formation from *α*-chaconine and *α*-solanine in wild potato *S. chacoense* requires an enzyme similar to the one catalyzing hydroxylation of *α*-tomatine, the major bitter SGA^7^ in the green tissues of related tomato species (Fig. 1). To discover the SGA hydroxylase enzyme, we implemented a QTL high-resolution mapping approach that couples profiling of selected leaf-associated SGAs with information derived from genomic bins in a tomato backcross inbred line (BIL) population^23^. Including the cultivated tomato (*Solanum lycopersicum cv*. M82) and wild species (*Solanum pennellii*) parents, the complete population set used in this study was composed of 671 lines. We analyzed the entire population by employing a leaf-dip method^24^ (*n* = 1) that allows high-throughput extraction of metabolites and a rapid (10 min per sample) LC-MS method to monitor 7 different SGAs: *α*-tomatine, *α*-tomatine isomer, dehydrotomatine, dehydrotomatine isomer, hydroxytomatine, acetoxytomatine, and di-dehydrotomatine (Fig. 2a, b, Supplementary Figs. 2a, 3a, 4a, 5a, and 6a, and Supplementary Data 1). We noted that *α*-tomatine was the most abundant SGA found in the leaves of the ILs and BILs, followed by dehydrotomatine and their respective isomers (Supplementary Figs. 2a, 3a, 4a, 5a, and 6a). On the contrary, hydroxytomatine (Figs. 2a and 3a), acetoxytomatine (Figs. 2b and 3b), and di-dehydrotomatine (Supplementary Fig. 6a) SGA levels were low across the population, and only specific IL and BIL lines accumulated them in relatively higher amounts. Consequently, specific genomic bins associated with differential accumulation of the seven SGAs were detected (Supplementary Fig. 7). We next validated the results by analyzing grounded leaf tissue extracts (*n* = 3) of 77 core ILs, the corresponding parents (Fig. 3a, b, and Supplementary Figs. 2b, 3b, 4b, 5b, and 6b), and selected BILs covering specific SGA-associated bin regions (Fig. 2c, d). While performing the leaf-dip extraction on hundreds of BILs and ILs, we realized that, specifically in *S. pennellii* leaves, extraction using the leaf-dip method was not very efficient due to the waxy nature of *pennellii* plant leaves and thus made them less amenable for extraction. Yet, this was not a problem for all the ILs and the cultivated tomato leaves as well as in the case of ground tissue extracts of *S. pennellii* leaves.

### Identification of the tomato GAME31 by QTL mapping

Five selected BILs (**#**6304, #6603, #6664, #6694, #6518) linked to the accumulation of hydroxytomatine and acetoxytomatine (Fig. 2) contained introgressions overlapping with the IL2-1 line on chromosome 2 (Fig. 3c). Consequently, we focused on a mapping bin region overlapped between the IL2-1 line and the selected BILs, spanning ~250 Kbp located on chromosome 2 (Fig. 3c) that represented a QTL associated with an increase in hydroxytomatine (Figs. 2a, c and 3a) and acetoxytomatine content (Figs. 2b, d and 3b). This bin contained 17 genes, 4 of them represented tandemly located putative *2-OXOGLUTARATE-DEPENDENT DIOXYGENASES* (*2-ODDs*) (Fig. 3c and Supplementary Data 2). The 2-ODD family represents one of the most versatile oxidative enzymes reported previously to catalyze hydroxylation reactions in specialized metabolism^25^. Two of the four 2-ODD genes were full-length sequences and termed *GLYCOALKALOID METABOLISM 31* (*GAME31*) and *GAME31-like* (Fig. 3c). It appeared that *GAME31* is highly expressed in ripening fruit tissues (RNA-seq expression data; available at https://www.ncbi.nlm.nih.gov/bioproject/PRJNA307656)^26^ as compared to leaf, petal, flower bud, and roots (Supplementary Fig. 8a) and hence significantly correlating with hydroxytomatine content during fruit development^9^. Thus fine metabolic QTL mapping indicated that GAME31 is possibly the enzyme carrying out the hydroxylation of *α*-tomatine to form hydroxytomatine in tomato.

### *GAME31* gene is highly expressed in IL2-1 and selected BILs

**Fig. 4:**
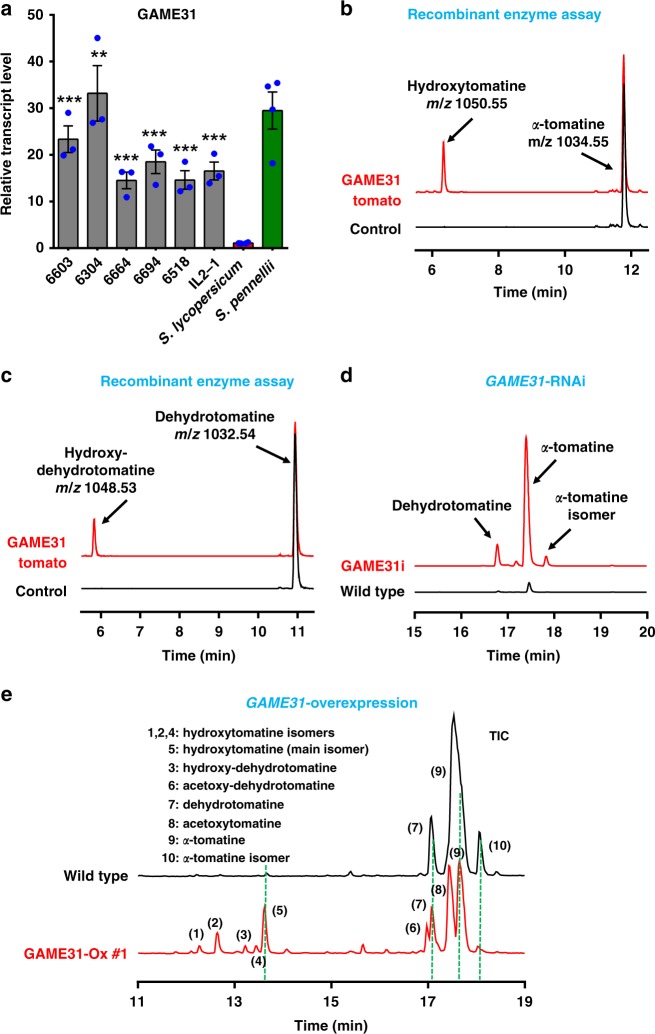
GAME31 catalyzes the hydroxylation of the bitter flavor *α*-tomatine in tomato fruit. **a** *GAME31* expression levels in leaves of selected BILs, IL2-1 line, and parents [*S. lycopersicum* (*cv*. M82) and *S. pennellii*] as determined by quantitative real-time PCR assay. The values indicate means of three biological replicates ± standard error mean (*n* = 3). Asterisks indicate a significant difference from *S. lycopersicum* samples calculated by Student’s *t* test (*P value < 0.05; **P value < 0.01; ***P value < 0.001). **b**, **c** Hydroxylation of *α*-tomatine (**b**) and dehydrotomatine (**c**) by the recombinant tomato GAME31 enzyme produced in *E. coli* cells (marked in red). Extracted ion chromatograms were obtained by LC-MS analysis (short 17-min run). The control reaction (shown in black) was performed with the respective substrate using extracts from *E. coli* cells transformed with an empty pET28 vector. *m*/*z* is shown for each substrate and hydroxylated product. Refer to Supplementary Fig. 11 for more information on the GAME31 enzyme assay with *α*-tomatine as a substrate. Details regarding the identification of enzyme assay products are provided in Supplementary Figs. 23 and 24. The structure of hydroxytomatine observed in the enzyme assay was elucidated using NMR analysis (refer to Supplementary Table 1). *m*/*z* mass to charge, *E. coli* *Escherichia coli*. **d** Red, ripe fruit of *GAME31*-silenced plants (RNAi; *GAME31i*) accumulated high levels of *α*-tomatine and dehydrotomatine as compared to wild-type fruit. Extracted ion chromatograms are shown. See Supplementary Fig. 15 for detailed quantitative analysis of SGAs in *GAME31* RNAi and *GAME31* co-suppression transgenic lines. **e** *GAME31*-overexpressing leaves (*GAME31*-Ox*)* accumulate hydroxytomatine isomers and acetoxytomatine, while these SGAs are present in minor quantities in wild-type leaves. Precursor SGAs, *α*-tomatine and dehydrotomatine, were reduced substantially in *GAME31*-Ox lines compared to wild type. Total ion chromatograms are shown. Lines #1 and #2 are two independent *GAME31*-Ox transgenic lines (#1 is shown here as a representative transgenic line). See Supplementary Figs. 16 and 18 for quantitative SGA analysis in *GAME31*-Ox transgenics with statistical information. The source data of Fig. 4a are provided as a Source Data file

SGA profiling in ILs and BILs showed that hydroxytomatine is a low-abundant SGA in the leaves of cultivated tomato (*S. lycopersicum cv*. M82) and wild species *S. pennellii* (Figs. 2a, c and 3a). Yet, all selected BILs (**#**6304, #6603, #6664, #6694, #6518) carrying *S. pennellii* introgressions mapping to IL2-1 showed very high levels of hydroxytomatine in leaf tissue as compared to the parental lines (Figs. 2c and 3a). It appeared that, out of the 17 bin genes, merely *GAME31* displayed differential expression in leaves of the IL2-1 line across the core IL population (obtained from previously reported leaf transcriptome data of the IL population^27^ ((10.5061/dryad.rm5v5); Supplementary Fig. 8b). We next compared the *GAME31* expression levels in the leaves of IL2-1, selected BILs and parental lines by quantitative real-time PCR (qPCR). In cultivated tomato (*cv*. M82), *GAME31* showed very weak expression in leaf tissue (Fig. 4a), correlating with low levels of hydroxytomatine in this genotype (Figs. 2c and 3a). In the *S. pennellii*, *GAME31* was highly expressed in leaves (Fig. 4a), yet this parent genotype contained very low levels of hydroxytomatine in leaves (Figs. 2c and 3a). Notably, *S. pennellii* leaves accumulate high levels of acetoxytomatine, the SGA derived from hydroxytomatine (Figs. 2d and 3b). Therefore, *pennellii* parent has most likely intense activity of the acyltransferase enzyme that acetylates hydroxytomatine, and the low hydroxytomatine levels we detected in the ground tissue extracted *S. pennellii* leaves in Figs. 2c and 3a are due to the very intense turnover of most hydroxytomatine to acetoxytomatine. In contrast, cultivated tomato (*cv*. M82) produces trace amount of acetoxytomatine in leaves (Figs. 2d and 3b). Thus the strong expression of *GAME31* in *S. pennellii* likely results in higher levels of hydroxytomatine in the leaves, which are immediately being converted to the downstream acetoxytomatine SGA product. Similar to the *pennellii* parent, all selected BILs and IL2-1 lines displayed very high *GAME31* gene expression as compared to the cultivated tomato leaves (Fig. 4a). This correlated with significantly higher hydroxytomatine content in their leaf tissue (Figs. 2c and 3a). Moreover, these lines also accumulate downstream acetoxytomatine in the leaves as the *pennellii* parental line (Figs. 2d and 3b).

To investigate the molecular basis of *GAME31* enhanced expression in the IL2-1 introgression, we compared GAME31 gene and protein sequences in the cultivated tomato (*cv*. M82) and *S. pennellii* genomes. The DNA alignment of both *GAME31* genes shows the presence of three exons and two introns (Supplementary Fig. 9a). The three exons and second intron showed high percentage of similarity, while the first intron was different between both variants, being ~4 times longer in the *S. pennellii* variant (565 base pairs (bp) in *S. lycopersicum* vs 2594 bp in *S. pennellii*) (Supplementary Fig. 9a). The GAME31 variants shared 96% and 95% homology with each other at the nucleic acid and amino acid levels, respectively (Supplementary Fig. 9b, c). Alignment of the 2000 bp upstream genomic region showed high conservation apart from the region ~400 to ~1000 bp upstream of the transcription start site (Supplementary Fig. 10). These differences in promoter region of *GAME31* between two parents might be responsible for driving the *GAME31* differential expression.

### GAME31 catalyzes hydroxylation of spirosolane-type SGAs

We next tested the capacity of *Escherichia coli* expressed recombinant GAME31 protein (from tomato) to hydroxylate SGAs and their corresponding steroidal alkaloid aglycones (SAAs) typically produced by tomato (e.g. tomatidine, *α*-tomatine, and dehydrotomatine), potato (e.g., solanidine, *α*-solanine, and *α*-chaconine), and eggplant (e.g. solasodine and *α*-solamargine). A reaction containing GAME31, *α*-ketoglutarate, ascorbate, Fe^2+^, and *α*-tomatine as a substrate produced hydroxytomatine (Fig. 4b). The hydroxytomatine peak observed here was identical to the one detected in the BIL/IL screening (Supplementary Fig. 11). Moreover, an additional two small peaks of hydroxytomatine isomers were also detected in the GAME31 enzyme assay as the commercial standard of *α*-tomatine apparently contained small amount of *α*-tomatine isomers as impurity (Supplementary Fig. 11). GAME31 could also catalyze the reactions forming hydroxy-dehydrotomatine, hydroxy-solasodine, and hydroxy-solamargine from dehydrotomatine, solasodine, and *α*-solamargine, respectively (Fig. 4c and Supplementary Fig. 12a, b). On the other hand, it was not active with any of the characteristic potato alkaloids, i.e. the solanidane type; *α*-solanine, *α*-chaconine, and solanidine. We next identified the putative homolog of tomato GAME31 in cultivated eggplant (*Solanum melongena*) that typically produces *α*-solasonine, *α*-solamargine, and minor amounts of hydroxylated products (Supplementary Fig. 13). The recombinant eggplant GAME31 enzyme showed similar substrate preference as the tomato enzyme (toward the spirosolane-type tomato and eggplant SGAs) and was likewise inactive with the solanidane-type potato alkaloids (Supplementary Fig. 14a–d). Hydroxylated metabolites (enzyme assay products) were putatively identified by comparing their retention times, elemental composition, and fragmentation pattern with those described in the literature^8,10^. Moreover, an additional MS-MS analysis was performed to identify the structures of the enzymatic reaction products (for detailed MS-MS analysis, see Supplementary Figs. 23–26).

### Functional characterization of *GAME31* in tomato

The in vivo function of GAME31 was examined by altering its expression by silencing and overexpression in tomato. Silencing *GAME31* (*GAME31i*) in tomato by RNA interference (RNAi) and co-suppression detected in some lines constitutively expressing the tomato *GAME31* (*GAME31* CoSup) resulted in dramatic accumulation of *α*-tomatine and dehydrotomatine. (Fig. 4d and Supplementary Fig. 15a). These SGAs, typically found in minor quantities in ripe fruit^8^, showed up to 22-fold increase in red ripe fruit of the silenced lines as compared to wild-type ones. Inversely, SGAs produced downstream of the *α*-tomatine hydroxylation reaction step toward esculeoside A (e.g. hydroxytomatine and acetoxytomatine) were reduced substantially in the *GAME31i* and *GAME31* CoSup ripe fruit (Supplementary Fig. 15b, see Supplementary Fig. 1 for detailed SGA pathway steps and intermediates). We observed similar trend of alterations in SGA accumulation earlier in *GAME31i* fruit development (Supplementary Fig. 15c, d). Leaves of *GAME31*-overexpressing (*GAME31*-Ox) tomato plants displayed ~4-fold reduction in *α*-tomatine and dehydrotomatine levels and accumulated 10–45 times higher hydroxytomatine (main isomer) and its isomers, as well as acetoxytomatine, that are typically present in minor levels in this tissue (Fig. 4e, Supplementary Fig. 16a, 16b and Supplementary Fig. 1 for detailed SGA pathway). The major hydroxytomatine peak we observed here is identical to the one detected in the recombinant enzyme assays and BIL/IL screening (Supplementary Fig. 17). Moreover, hydroxytomatine isomers detected in *GAME31*-Ox lines were the ones observed in the selected BILs and IL2-1 (Supplementary Fig. 17). *GAME31*-Ox green fruit displayed reduction in *α*-tomatine, *α*-tomatine isomer, and dehydrotomatine levels, whereas major accumulation of hydroxytomatine and its downstream SGAs (e.g. acetoxytomatine, acetoxy-hydroxytomatine, acetoxy-dehydrotomatine) was observed as compared to wild-type green fruit (Supplementary Fig. 18a, b). In red ripe fruit of the *GAME31*-Ox lines, we detected increased accumulation of esculeoside A, at the expense of *α*-tomatine (Supplementary Fig. 18c). Furthermore, we detected a major increase in hydroxytomatine and its downstream SGAs in comparison to wild-type red ripe fruit (Supplementary Fig. 18c). Altering *GAME31* expression (i.e. *GAME31*-Ox and *GAME31i*) in tomato did not affect normal growth and development of transgenic plants compared to wild-type tomato plants. The structure of major hydroxytomatine peak observed in the BILs, ILs, recombinant enzyme assays, and stable transgenic plants was unambiguously determined using nuclear magnetic resonance (NMR) analysis (Supplementary Table 1, Supplementary Figs. 33–40). Altogether, these results demonstrated that GAME31 catalyzes the hydroxylation of *α*-tomatine to hydroxytomatine, the first committed step toward esculeoside A and related SGA formation in ripe tomato fruit.

### GAME32 hydroxylates the potato SGAs to produce leptinines

Following our finding that GAME31, a 2-ODD family enzyme, was able to hydroxylate tomato and eggplant alkaloids, we continued our search for 2-ODD-type enzymes that could hydroxylate potato SGAs to form leptinines in two different wild potato *S. chacoense* accessions (i.e. 8380-1 and M6). Both leptinines and leptines are foliar-specific SGAs and therefore accumulate predominantly in leaves, stem and green floral organs as compared to stolons, roots, and tuber skin tissues of *S. chacoense* (*Sc*)^28^. Through sequence similarity search, we identified *GAME31* homologs in the *S. chacoense* M6 genome (on unknown chromosome) and the 8380-1 accession (termed *ScGAME31-M6* and *ScGAME31-8380*-1, respectively). These proteins share 87% and 80% homology with the tomato and eggplant GAME31 proteins, respectively. As in the case of the tomato and eggplant GAME31 proteins, both *S. chacoense* GAME31 recombinant enzymes exhibited hydroxylase activity explicitly on spirosolane-type tomato and eggplant substrates (e.g. *α*-tomatine, dehydrotomatine, tomatidine, *α*-solamargine, etc.) but not with the solanidane-type substrates characteristic for potato (e.g. *α*-chaconine, *α*-solanine, etc.) (Supplementary Figs. 19 and 20 and see Supplementary Figs. 23–27 for MS-MS analysis).

**Fig. 5:**
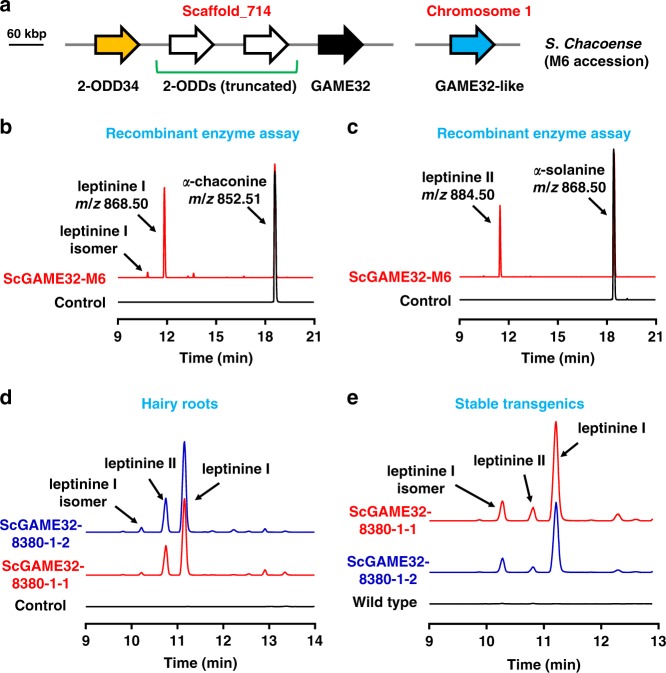
GAME32 hydroxylates the main potato SGAs to form CPB resistance-associated leptinines. **a** Schematic presentation of the 2-ODD family members *GAME32* (black), *GAME32-like* (blue), and *2-ODD34* (yellow) genes in the *S. chacoense* (*Sc*) genome (accession M6). Truncated 2-ODDs are shown in white block arrows. **b**, **c** The recombinant ScGAME32-M6 enzyme converts *α*-chaconine (**b**) and α-solanine (**c**) to leptinine I (plus isomer) and leptinine II, respectively. Red: reaction assay with the respective substrate and the recombinant *S. chacoense*-M6 GAME32 protein (produced in *E. coli*); black: control reaction with the respective substrate and the protein extracts of empty vector-transformed *E. coli*. Mass to charge (*m*/*z*) is shown for all substrates and assay products. MS-MS analysis and identification of leptinines metabolites are provided in Supplementary Figs. 29–32. **d** De novo production of leptinines in cultivated potato hairy roots overexpressing *ScGAME32-8380-1-1* (in red) or *ScGAME32-8380-1-*2 (in blue) as compared to control (in black) hairy roots (generated with an empty vector). **e** SGA profiles in leaves of wild type (non-transformed) and *ScGAME32-8380-1-1* or *ScGAME32-8380-1-*2 overexpressing stably transformed cultivated potato plants determined by LC-MS. As observed for hairy roots, accumulation of leptinines was detected in the leaves of stably transformed cultivated potato plants. Extracted ion chromatograms are presented in all cases. Refer to Supplementary Fig. 22c, d for quantitative data on SGA analysis

As sequence similarity was not sufficient to identify 2-ODD gene candidates associated with potato leptinine biosynthesis, we mined the publicly available *S. chacoense* M6 accession transcriptome data^29^ and detected two putative *2-ODD* homologs showing the expression profiles correlating with the previously reported leptinine accumulation pattern in different tissues^28^. The corresponding proteins annotated as *ScGAME32-M6* and *ScGAME32-like-M6* shared 94% homology. While *ScGAME32-like-M6* is located on chromosome 1, *ScGAME32-M6* is assigned to scaffold_714 (~60 Kbp long) of an unknown chromosome along with three other 2-ODD genes (Fig. 5a). Two of the four genes in scaffold_714 appeared truncated and displayed very low expression in the transcriptome data. The full-length fourth gene (*Sc2-ODD34-M6*) showed comparable expression pattern to *ScGAME32-M6* and *ScGAME32-like-M6* genes and shared 82% homology with these two proteins. Next, we expressed ScGAME32-M6, ScGAME32-like-M6, and Sc2-ODD34-M6 proteins separately in *E. coli* and tested their activity with different SAA/SGA substrates. Incubation of the ScGAME32-M6 recombinant enzyme with *α*-chaconine and *α*-solanine as substrates resulted in the formation of hydroxylated products; i.e. leptinine I and leptinine II, respectively (Fig. 5b, c). An additional leptinine I isomer was also observed in the enzyme assay as *α*-chaconine analytical standard contained isomer impurities (Fig. 5b). The ScGAME32-M6 enzyme was also able to produce hydroxysolanidine in vitro when incubated with solanidine, the potato steroidal alkaloid aglycone (Supplementary Fig. 21a; for MS-MS analysis, see Supplementary Fig. 28). Yet, it did not show activity with tomato and eggplant SAA/SGA substrates. Interestingly, ScGAME32-like-M6 and Sc2-ODD34-M6 did not exhibit hydroxylase activity with either *α*-chaconine or *α*-solanine substrates and neither with one of the tomato nor eggplant SAA/SGA substrates. Leptinines (leptinine I and II) were putatively identified by comparing their retention times, elemental composition, and fragmentation pattern with those described in the literature^10,28^. Moreover, an additional MS-MS analysis was performed to identify the leptinine structures (see Supplementary Figs. 29–32).

From the high leptine-producing *S. chacoense* 8380-1 accession, we isolated two isoforms of GAME32 (termed ScGAME32-8380-1-1 and ScGAME32-8380-1-2) and examined the corresponding recombinant enzyme activity for leptinine production. Incubation of ScGAME32-8380-1-1 or ScGAME32-8380-1-2 with *α*-chaconine and *α*-solanine resulted in the formation of leptinine I along with its isomer and leptinine II, respectively (Supplementary Fig. 21b–e). The *S. chacoense* 8380-1 accession enzymes did not show any activity with the tomato and eggplant SAA/SGA as substrates. Thus we found that ScGAME32 enzymes from the M6 and 8380-1 accessions carry out hydroxylation specifically on SGAs of the solanidane type but not on spirosolane-type substrates.

To test the in vivo activity of ScGAME32-8380-1-1 and ScGAME32-8380-1-2, we generated hairy root lines in the cultivated potato background (using *Agrobacterium rhizogenes*-mediated transformation) (Supplementary Fig. 22a) and stable transgenic plants overexpressing them (Supplementary Fig. 22b). In transformed 5–7-week-old hairy roots overexpressing either *ScGAME32-8380-1-1* or *ScGAME32-8380-1-2*, we detected de novo production of leptinine I isomers and leptinine II SGAs (Fig. 5d and Supplementary Fig. 22c). This was accompanied by a significant reduction in *α*-chaconine and *α*-solanine levels (Supplementary Fig. 22c). Consistent with the hairy root results, we also observed the accumulation of leptinine I isomers and leptinine II at the expense of main SGAs in the leaves of the stable transgenic potato plants overexpressing *ScGAME32-8380-1-1* or *ScGAME32-8380-1-*2 (Fig. 5e and Supplementary Fig. 22d). Moreover, transgenic potato plants overexpressing *ScGAME32-8380-1-1* or *ScGAME32-8380-1-*2 showed normal growth and developmental phenotype compared to wild-type (non-transformed) cultivated potato.

### Absence of GAME32 prevents leptinines in cultivated potato

**Fig. 6:**
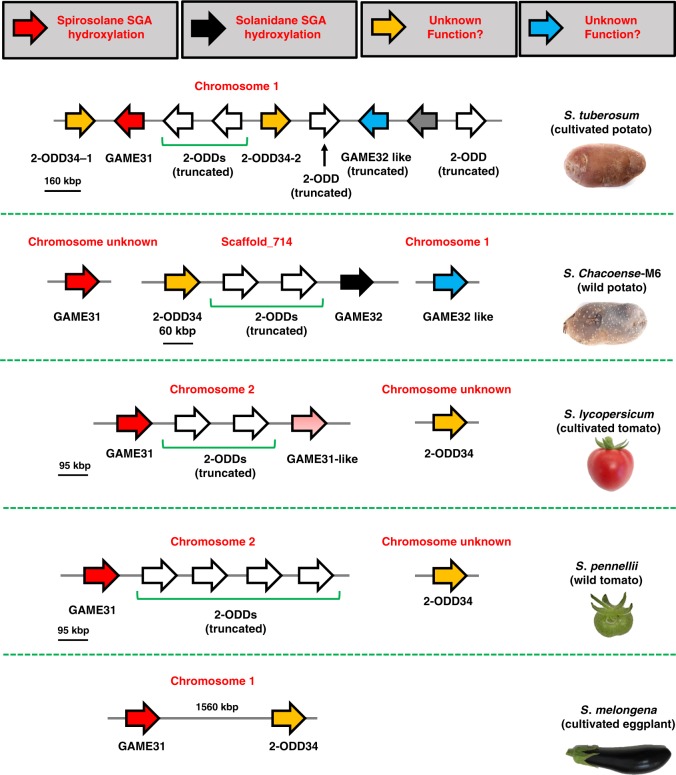
Genomic organization of 2-ODD family genes in selected *Solanum* species associated with SGA metabolism. Four 2-ODD genes, namely, *GAME31* (red block arrow), *GAME32* (black block arrow), *GAME32-like* (blue block arrow), and *2-ODD34* (yellow block arrow), were selected for functional characterization in this study. We proposed a function for *GAME31* and *GAME32* genes based on in vivo and/or in vitro experiments. While *GAME31* and *2-ODD34* homologs are present in all *Solanum* species examined, the leptinine-producing *GAME32* gene appears to be unique to *S. chacoense*, wild potato species that possess genetic CPB pest resistance. Cultivated potato lacks the *GAME32* gene, resulting in absence of leptinines and leptine SGAs, and is therefore susceptible to CPB. Truncated 2-ODDs are shown in white block arrows. The arrowheads represent direction of transcription

The next question we asked was what explains the lack of leptinines/leptines in cultivated potato and their unique accumulation in the wild potato *S. chacoense*. We detected eight clustered *2-ODD* genes in the cultivated potato chromosome 1 spanning a ~160-Kbp region that includes a single *GAME31*, one truncated *GAME32-like*, and two *2-ODD34* homologs (Fig. 6). Four of the eight gene sequences in the cluster appeared partial. A clear homolog of the leptinines producing *GAME32* gene of *S. chacoense* species is not present in cultivated potato (Fig. 6). Individual recombinant assay of the cultivated potato GAME31, GAME32-like, and the pair of 2-ODD34 enzymes did not show hydroxylase activity with *α*-chaconine, *α*-solanine, or with other tomato and eggplant SAA/SGA substrates. The genomic organization of GAME31, GAME32, GAME32-like, and 2-ODD34 genes from selected *Solanum* species is presented in Fig. 6. The absence of the *GAME32* gene in cultivated potato therefore explains the lack of leptinines and downstream leptine SGAs (Fig. 6).

**Fig. 7:**
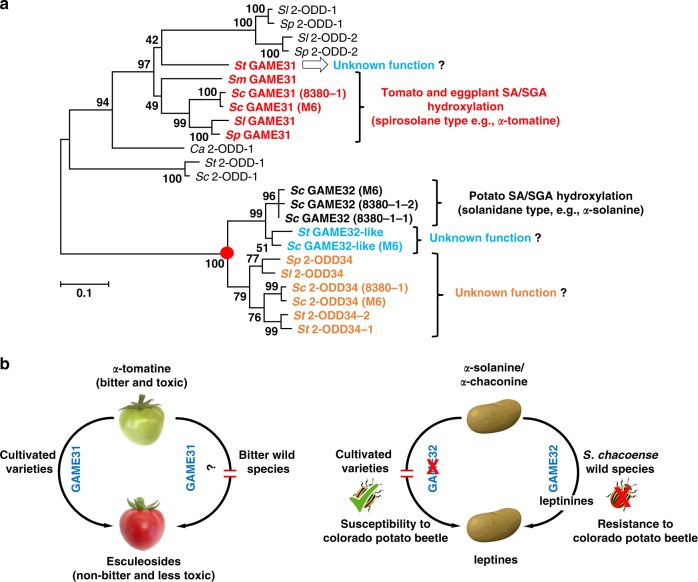
Pathways to unique defense metabolites and for evading fruit bitterness evolved through 2-ODD activity. **a** GAME proteins (GAME31 and GAME32) carrying out typical hydroxylation reactions in *Solanum* SGA metabolism and other putative candidates (GAME32-like and 2-ODD34) form distinct clades in the 2-oxoglutarate-dependent dioxygenase (2-ODD) family. Sequences from the following species were represented: cultivated tomato [*S. lycopersicum*, (*Sl*)], cultivated potato [*S. tuberosum*, (*St*)], wild tomato [*S. pennellii*, (*Sp*)], *Capsicum annuum* (*Ca*), cultivated eggplant [*S. melongena*, (*Sm*)], and wild potato [*S. chacoense*, (*Sc*)]. GAME31, GAME32, GAME32-like, and 2-ODD34 proteins forming separate clades are depicted in red, black, blue, and brown colors, respectively. Amino acid sequences used in the phylogenetic analysis are provided in Supplementary Data 3. Red dot denotes common ancestor shared between GAME32 and 2-ODD34 clade proteins. **b** A model presenting the impact of GAME31 and GAME32 proteins activity on tomato fruit flavor (bitter/sweet) and fruit toxicity as well as resistance of potato plants to CPB insect pest. Activity of GAME31 is required to modulate bitterness and toxicity in ripe tomato fruit. Green tomato fruit accumulates a bitter and toxic *α*-tomatine SGA. As the fruit matures reaching the red stage, the entire *α*-tomatine pool is converted to non-bitter and less toxic esculeosides. In cultivated tomato varieties, GAME31 performs the first reaction step (i.e. hydroxylation) that ensures degradation of *α*-tomatine and results in non-bitter and harmless ripe fruit. On the contrary, red ripe fruit of certain wild tomato species (e.g. *S. lycopersicum* var. *cerasiforme*) are bitter in flavor due to high accumulation of *α*-tomatine^7^. These wild species are apparently altered in the process of *α*-tomatine catabolism. GAME32 activity in *S. chacoense* wild potato is vital for its genetic resistance against Colorado potato beetle (CPB). Through the action of the GAME32 enzyme, *α*-chaconine and *α*-solanine are first converted to leptinines and further to downstream leptines, respectively, in *S. chacoense*, which makes this wild species naturally resistant to CPB. The leptinine-producing *GAME32* gene is absent in the cultivated potato genome, thus preventing formation of further leptines and this likely makes cultivated potato CPB susceptible

The sequence similarity observed in the phylogenetic analysis reflects the activity of the various 2-ODD GAME proteins reported in this study (Fig. 7a). Tomato, eggplant, and *S. chacoense* GAME31 enzymes that act on spirosolane-type SAAs/SGAs form a separate clade. 2-ODD34 enzymes that do not partake in SGA hydroxylation form a large separated clade from the rest of the GAME31, GAME32, and GAME32-like proteins, suggesting possibly a unique divergent function for these enzymes in SGA metabolism rather than typical hydroxylation (Fig. 7a). *S. chacoense* GAME32 proteins that hydroxylate solanidane-type SGAs forming leptinines localize to a distinct clade and yet share a common ancestor with 2-ODD34 proteins present in all *Solanum* species we examined (Fig. 7a). The exclusive presence of GAME32 proteins in *S. chacoense* suggest its late independent evolution compared to 2-ODDs present in other *Solanum* species (e.g. GAME31 or 2-ODD34).

## Discussion

SGAs including *α*-tomatine are renowned defense metabolites possessing toxicity to a broad range of pathogens and predators, including bacteria, fungi, insects, oomycytes, viruses, and animals^1–4^. However, *α*-tomatine also impacts tomato fruit flavor. Early work by Charles Rick and co-workers demonstrated a substantial association between the presence of high *α*-tomatine content and bitter flavor in ripe fruit of wild cherry tomato (*S. lycopersicum* var. *cerasiforme*) accessions found in the Río Mayo valley, Peru^7^. The authors suggested that a recessive, likely monogenic mutation in the enzyme catalyzing the typical degradation of *α*-tomatine during fruit ripening resulted in *α*-tomatine accumulation and bitterness. With similarity to tomato, metabolism of the core SGA produced in young fruit (e.g. solasodine) during ripening has been reported in other *Solanum* species including *S. nigrum*, *S. sysimbrifollium*, and *S. mammosum*^30–32^. The presence of high *α*-tomatine (or other toxic SGAs in different *Solanum* species) in early stage of development protects fruit from pathogens and herbivores. In ripe tomato fruit, the entire pool of *α*-tomatine is converted to non-bitter and less toxic esculeosides (Fig. 7b). This reduction in toxicity and bitterness, along with development of flavor traits (i.e. aroma, sugar, and acid content) in ripening, facilitates feeding by frugivors and consequently promotes seed dispersal. The retention of toxic and bitter alkaloids in mature ripe fruit of the bitter *cerasiforme* accessions was not an adaptive trait as humans have been selecting fruit with increased sweetness and reduced toxic SGA content. We therefore propose that tomato GAME31 performs the first enzymatic step in a catabolic pathway that evolved and later conserved through domestication and breeding to assure a less bitter and harmless ripe fruit as consumed at present.

CPB known as an international super-pest has become the most devastating herbivore pest of cultivated *Solanum* species. Despite the presence of high levels of *α*-chaconine and *α*-solanine, cultivated potato varieties are highly susceptible to CPB (Fig. 7b). However, the wild potato species *S. chacoense* possesses genetic resistance against CPB likely due to the presence of foliar leptinines and leptines (Fig. 7b). Long-term breeding programs in the past 30 years using high leptine *S. chacoense* genotypes failed to develop CPB-resistant cultivated potato varieties. Recently, CPB-resistant transplastomic potato plants were generated by employing an RNAi construct that targeted an essential CPB gene^33^. Identification of the *S. chacoense GAME32* genes in this study may pave the way for alternative strategies to obtain CPB resistance in cultivated potato and other *Solanum* crops through breeding and metabolic engineering of leptinine/leptine insecticides.

## Methods

### Plant material

The tomato IL and BIL population were derived from crosses of the *S. lycopersicum cv*. M82 and *S. pennelli*^23,34^. The entire population set consisted of 671 lines, including the two parental lines M82 and *S. pennellii*, F1 hybrid, 132 ILs, and 536 BILs. IL and BIL population plants, tomato (*S. lycopersicum cv*. Micro Tom), cultivated potato (*Solanum tuberosum cv*. Desiree), and *S chacoense* (accession M6 and 8380-1) plants were grown in a climate-controlled greenhouse at 24 °C during the day and 18 °C during night, with natural light. The genotyping of IL and BIL population were carried out using an Illumina 10 K SNP-chip^23^. The genotyping analysis was performed by the Trait Genetics GmbH genotyping service, according to the manufacturer’s instructions. Furthermore, selected SGA profiles (in total 7 SGAs) and genotypic data of BILs and ILs were used to map the QTL regions on tomato chromosomes^23^.

### Generation of *GAME31* transgenic tomato plants

The *GAME31*-RNAi construct was created by introducing a 298-bp fragment (Forward oligonucleotide: GCGGCCGCATGGATTGATGTGATTATTTCACC, Reverse oligonucleotide: GGCGCGCCCTTGAAAAGATCACTTGGAGGAG) to pENTR/D-TOPO (Invitrogen) (by NotI and AscI) and further transfer of the resulting cloned fragment to the pK7GWIWG2 (II) binary vector^35^ using the Gateway LR Clonase II enzyme mix (Invitrogen). The *GAME31*-Ox (overexpression) construct was generated using Goldenbraid cloning^36^. Tomato *GAME31* coding sequence was initially cloned into the pUPD vector and further moved into the respective 3α2 and 3Ω1 vectors, which are based on the pCAMBIA backbone. Constructs were transformed into tomato (*cv*. Micro Tom) using *Agrobacterium tumefaciens* (strain GV3101) mediated transformation. Briefly, cotyledon explants were excised from the 7-day-old in vitro grown tomato seedling and cut near the petiole and tip, placed on a plate containing appropriate co-cultivation media, and preincubated for 24 h at room temperature (RT) under dark conditions. Co-cultivation of excised explants with agrobacterium (OD_600_ = 0.3) was carried out for 48 h under dark conditions. After co-cultivation period, explants were transferred to shoot induction medium containing zeatin (2 µg ml^−1^), indole-3-acetic acid (IAA; 0.2 µg ml^−1^), Kanamycin (50 µg ml^−1^), and Ticarcillin (250 µg ml^−1^) for 3–4 weeks and then transferred again on shoot elongation medium containing zeatin (1 µg ml^−1^), zeatin riboside (1 µg ml^−1^), IAA (0.2 µg ml^−1^), Kanamycin (50 µg ml^−1^), and Ticarcillin (100 µg ml^−1^). Subsequently, well-developed shoots were excised and transferred to rooting medium containing indole-3-butyric acid (IBA; 2 µg ml^−1^), Kanamycin (50 µg ml^−1^), and Ticarcillin (100 µg ml^−1^). After 3–4 weeks, plantlets with roots were transferred to greenhouse for further analysis. Positive transgenic lines were selected by qPCR and further used for LC-MS-based metabolite analysis. Oligonucleotides used in this study are provided in Supplementary Data 4.

### Generation of transgenic potato plants

*ScGAME32(8380-1-1)* and *GAME32(8380-1-2)* coding sequences from *S. chacoense* accession 8380-1 were cloned separately into 3Ω1 vector using Goldenbraid cloning^36^. Constructs were transformed into cultivated potato (*cv*. Desiree) using *A. tumefaciens* (strain GV3101) mediated transformation and young leaves as explants. After co-cultivation period, explants were transferred to shoot induction medium containing naphthaleneacetic acid (NAA; 1 µg ml^−1^), 6-benzyl aminopurine (1 µg ml^−1^), Kanamycin (50 µg ml^−1^), and Ticarcillin (200 µg ml^−1^) for 3–4 weeks and then transferred again on shoot elongation medium containing NAA (1 µg ml^−1^), zeatin riboside (1 µg ml^−1^), Kanamycin (50 µg ml^−1^), and Ticarcillin (100 µg ml^−1^). Subsequently, well-developed shoots were excised and transferred to rooting medium containing IBA (2 µg ml^−1^), Kanamycin (50 µg ml^−1^), and Ticarcillin (100 µg ml^−1^). After 3–4 weeks, plantlets with roots were transferred to greenhouse for further analysis. Positive transgenic lines were selected based on qPCR and further used for metabolite analysis.

### Generation of transgenic potato hairy roots

Hairy roots were generated from leaves of in vitro grown cultivated potato (*S. tuberosum cv*. Desiree) plants infected with the *A. rhizogenes* strain ATCC 15834, harboring the respective gene constructs (here, *ScGAME32(8380-1-1)* or *GAME32(8380-1-2)*). From glycerol stock, the *Agrobacterium* cells were inoculated on agar solidified nutrient broth (NB) medium supplemented with biotin (0.22 µg ml^−1^) and incubated at 28 °C. After 48 h of incubation, a single colony was inoculated into 2 ml of NB (nutrient broth) liquid medium supplemented with appropriate amount of antibiotics and biotin and cultured for 24 h at 200 rpm, 28 °C. From the overnight culture, 200 μl was inoculated into 10 ml NB supplemented with biotin and specific antibiotics. The culture was grown to OD_600_ = 0.6–0.8, pelleted at 2500 × *g* for 10 min at RT, and suspended to OD_600_ = 0.5 with MS3S (Murashige and Skoog medium + 3% sucrose) supplemented with 50 μM acetosyringone and cultured for additional 1.5–2 h on a horizontal shaker at 200 rpm. The proximal ends of young potato leaves were removed a few mm above the petiole while immersed in MS3S liquid (with agrobacterium) medium, pH 5.8, in 90 mm Petri dishes, incubated for 20 min with occasional shaking, and blotted on sterile filter papers. About 10–15 explants were placed (the adaxial side upward) on semisolid MS3S medium in 90 mm Petri plates, and the plates were sealed with saran wrap and co-cultivated in the dark at 28 °C for 2 days. After 2 days of co-cultivation, 6–8 leaves were transferred to 90 mm Petri plates with semisolid MS3S medium supplemented with Kanamycin (50 µg ml^−1^), Cefotaxime (500 µg ml^−1^), and the ethylene action inhibitor, silver thiosulfate (STS, 0.2 mM), and incubated in light (150 μmol m^−2^ s^−1^, 16 h) at 25 °C. The leaves were transferred to fresh medium every 15 days and individual roots (2–3-cm long) that appeared after 20–25 days of incubation were excised and transferred to 1/2MS+1.5% sucrose medium supplemented with Kanamycin (50 µg ml^−1^) and Cefotaxime (300 µg ml^−1^) for selection. Roots that grew on the selection medium were transferred to 20 ml 1/2MS+1.5% sucrose liquid medium, with antibiotics, in 125-ml Erlenmeyer flasks and incubated in the dark on a horizontal shaker (90 rpm). After 1–2 weeks in liquid medium, hairy roots were harvested, washed with water, subsequently frozen, and ground in liquid N_2_. Positive hairy root transgenic lines (*n* = 3, 3 independent transgenic hairy root lines for each gene construct) were selected by qPCR. Furthermore, 100 mg tissue from positive lines (*n* = 3) was used for LC-MS-based SGA measurements as described below.

### Analytical standards

Analytical standards including tomatidine (Sigma-Aldrich, contains dehydrotomatidine as impurity), solanidine (ChemFaces), solasodine (ChemFaces), *α*-tomatine (Carbosynth USA, contains dehydrotomatine as impurity), *α*-solanine (ChemFaces), *α*-chaconine (ChemFaces), and *α*-solamargine (ChemFaces) were dissolved in methanol at a concentration of 1 mg ml^−1^.

### Screening of SGAs in the tomato IL and BIL populations

To screen for SGAs, we developed a rapid extraction method and a short 10-min run in the ultra-performance liquid chromatography (UPLC)-quad time of flight (qTOF)-MS (Xevo, Waters), based on Schilmiller et al.^24^. For extraction, we selected the leaflet next to the youngest leaf (about ~1-month-old plants). The leaflet was dipped in 1 ml of isopropanol:acetonitrile:water (3:3:2 v/v) containing 0.1% formic acid (in a 2-ml Eppendorf tube) and gently rocked for 1 min. The solvent mix was then transferred to a vial for injection to the LC-MS. The leaflets were dried in an oven to obtain their weight (mg). Data from UPLC-qTOF-MS analyses was manually inspected in order to determine the major SGA metabolites present in the population. *α*-tomatine and dehydrotomatine SGAs were identified using authentic standard compounds (see the “Analytical standards” section above) by comparison of their retention times and mass fragments. For other SGAs (*α*-tomatine isomer, dehydrotomatine isomer, di-dehydrotomatine, acetoxytomatine, and hydroxytomatine), elemental composition and MS-MS fragmentation patterns were compared with those described in literature for their putative identification^8^. Peak area quantification was performed using the program TargetLynx (Waters). The resulting values were normalized to dry leaf weight and considered to be the amount of metabolite present in each sample. For ground tissue, preparation of plant extracts and metabolite analysis by UPLC-qTOF-MS was carried out as follows: Briefly, tomato plant tissues were frozen in liquid nitrogen and ground to a fine powder using an analytical mill or mortar/pestle. Then frozen tissue (100 mg) was extracted with 80% methanol containing 0.1% formic acid [the solid:liquid ratio was kept at 1:3 (w/v)]. The mixture was vortexed for 30 s, sonicated for 30 min at RT, vortexed again for 30 s, centrifuged (13,000 × *g*, 10 min), and filtered through a 0.22-µm polytetrafluoroethylene membrane filter. Targeted metabolite analysis was performed using the TargetLynx program (Waters).

### Plant extract preparation and targeted profiling of SGAs

Preparation of extracts and the profiling of SGAs in various tomato tissues (leaves, green, and red fruit), potato leaves, and potato hairy roots were performed with the same methods described in literature^6,8^. Three biological replicates (*n* ≥ 3) from each genotype were used for metabolite analysis (e.g. #1, #2, and #3 are three independent *GAME31*-CoSup transgenic lines, and each transgenic line represents three biological samples collected from three different plants). SGA metabolites were identified by comparing the retention time and mass spectra of authentic standards analyzed on the same instrument (see the “Analytical standards” section above). When the corresponding standards were not available, SGA metabolites were putatively identified by comparing their retention times, elemental composition, and fragmentation pattern with those described in the literature^8,10,28,37^. For leptinine identification, two additional MS-MS analysis with a collision energy ramp from 20 to 80 eV and 120 eV (without ramp) was performed and the fragments were assigned by applying the fragmentation rules for steroid-based compounds^28,38^. Relative quantification of the steroidal glycoalkaloid metabolites was carried out using the TargetLynx (Waters) program.

### Quantitative real-time PCR

Gene expression analysis was performed with three biological replicates (*n* ≥ 3) for each genotype. RNA isolation was performed by the Trizol method (Sigma-Aldrich). DNase I (Sigma-Aldrich)-treated RNA was reverse transcribed using a High-capacity cDNA Reverse Transcription Kit (Applied Biosystems). Gene-specific oligonucleotides were designed with the Primer Express 2 software (Applied Biosystems). The *TIP41* and *NAC* gene^8,26,39^ were used as reference genes for tomato and potato respectively, in qPCR analysis.

### GAME31 expression in *E. coli* and protein purification

*GAME31* genes from tomato and eggplant were cloned separately into the pET28 vector by restriction free (RF) cloning using the same methods described in literature^40^. The oligonucleotides used for RF cloning are provided in Supplementary Data 4. The resulting plasmids were verified by sequencing and transformed to *E. coli* BL21 (DE3). For isolation of the GAME31-His-tagged protein, fresh overnight cultures were diluted 1:100 in 1000 ml of LB medium with 50 µg ml^−1^ kanamycin and incubated at 37 °C and 250 rpm until an OD_600nm_ of 0.5 was reached. Subsequently, for induction of expression of the recombinant proteins, isopropyl β-d-1-thiogalactopyranoside (IPTG) was added to a final concentration of 0.2 µM and the incubation was continued overnight at 15 °C and 250 rpm. Cells were harvested by centrifugation at 6000 × *g* and bacteria were lysed by sonication in a buffer (pH 7.4) containing 0.02 M NaH_2_PO_4_, 0.5 M NaCl, SIGMAFAST protease inhibitor tablets (Sigma-Aldrich), and benzonase nuclease (Sigma-Aldrich) according to the manufacturer’s instructions. Lysed cultures were centrifuged and the soluble fraction was purified by nickel affinity chromatography, which was operated with an AKTA liquid chromatography system (AKTA avant, GE Healthcare) according to the manufacturer’s instructions. Protein concentration was measured using the Bio-Rad protein assay by comparison to a BSA standard, and protein aliquots were stored at −80 °C until further analysis.

### Recombinant GAME31 enzyme assays

The steroidal alkaloid aglycones tomatidine, dehydrotomatidine, solanidine, and solasodine, as well as their glycosylated forms (i.e. SGAs) *α*-tomatine, dehydrotomatine, *α*-solanine, *α*-chaconine, and *α*-solamargine, were used as substrates in recombinant GAME31 enzyme assays. The recombinant GAME31 enzyme activity assay was performed according to Kawai et al.^41^ with minor modifications. Briefly, the standard full reaction (100 µl) consisted of 10 mM L-ascorbic acid, 10 mM *α*-ketoglutaric acid, 500 µM FeSO_4_, 5 µg substrate, 50 mM potassium phosphate buffer (pH 7.5), and purified enzyme. All the reaction components, except the enzyme, were preincubated for 10 min at 30 °C, after which the reaction was started by addition of the enzyme. After incubation at 30 °C for 3 h, the reaction was stopped by freezing in liquid nitrogen. Finally, the reaction was mixed with 250 µl methanol, extracted, and analyzed by UPLC-qTOF-MS using shorter (17 min) linear gradient with conditions: For steroidal aglycone substrates (e.g. tomatidine and solasodine), from 80% to 66% phase A over 13 min, from 66% to 0% phase A over 0.5 min, then held at 100% phase B for 1.5 min, and then returned to the initial conditions (80% phase A) within 0.5 min and conditioning at 80% phase A for 1.5 min; for steroidal glycoalkaloid substrates (e.g. *α*-tomatine and *α*-solamargine), from 90% to 77% phase A over 13 min, from 77% to 0% phase A over 0.5 min, then held at 100% phase B for 1.5 min, and then returned to the initial conditions (90% phase A) within 0.5 min and conditioning at 90% phase A for 1.5 min. The mobile phase consisted of 0.1% formic acid in acetonitrile:water (5:95, v/v; phase A) and 0.1% formic acid in acetonitrile (phase B). The flow rate was 0.3 ml min^−1^, and the column temperature was kept at 35 °C. Enzyme assay products (hydroxylated compounds) were identified based on accurate mass-derived elemental composition and MS-MS fragmentation pattern with those described in the literature^8,37,38^. The assignment of hydroxyl-group at C-23 position in hydroxytomatine was based on NMR of isolated hydroxytomatine compound from *GAME31*-Ox lines as described below (refer “Hydroxytomatine purification for NMR analysis” and “NMR methods” section). Protein extracts obtained from empty pET28 vector-transformed *E. coli* BL21 (DE3) cells were used in control reactions.

### *E. coli* expression and in vitro assays for GAME enzymes

*ScGAME31-M6*, *ScGAME31-8380-1*, *ScGAME32-M6*, *ScGAME32-8380-1-1*, *ScGAME32-8380-1-2*, and *ScGAME32-like* genes from the respective *S. chacoense* accession (M6 and 8380-1) and GAME31 and *GAME32-like* gene from cultivated potato (*cv*. Desiree) were separately cloned into the pET28b vector and expressed in *E. coli* BL21 (DE3) cells. Bacterial cells were grown in LB medium at 37 °C. When cultures reached *A*_600_ = 0.6, protein expression was induced with 200 μM of IPTG at 15 °C, for 24 h. Bacterial cells were lysed by sonication in 50 mM Tris-HCl pH 7.5, 500 mM NaCl, 10% glycerol, and protease inhibitor cocktail (Calbiochem). Soluble proteins were purified on Ni-NTA agarose beads (Adar Biotech) and eluted with 500 mM imidazole in buffer containing 50 mM NaH_2_PO_4_ (pH 7.5) and 300 mM NaCl. The whole-cell extract and the eluted fractions were analyzed by sodium dodecyl sulfate-polyacrylamide gel electrophoresis stained with InstatBlue. Assays with the recombinant GAME31, GAME32, and GAME32-like enzymes purified from *E. coli* cells using SAA/SGA substrates were performed under standard assay conditions, as described above. Enzyme assay products were analyzed by UPLC-qTOF-MS using standard (long gradient, 40 min) conditions as follows: from 100 to 72% phase A over 22 min, from 72 to 0% phase A over 14 min, then held at 100% phase B for 2 min; and then returned to the initial conditions (100% phase A) within 0.5 min and conditioning at 100% phase A for 1.5 min, unless stated otherwise. Other LC-MS parameters are same as described above. Enzyme assay products were identified based on accurate mass-derived elemental composition and MS-MS fragmentation pattern with those described earlier^8,10,28,38,42^. The control reaction was performed using protein extracts from *E. coli* cells transformed with empty pET28 vector.

### Recombinant 2-ODD34 protein expression and in vitro assays

Cloning, *E. coli* expression, and purification of Sc2-ODD34 (from M6 accession) and two 2-ODD34 from cultivated potato (*cv*. Desiree) were performed as described above for other GAME genes. In vitro recombinant 2-ODD34 enzyme (5 µg) assay with SAA/SGA substrates along with control reaction were carried out under standard assay conditions, as described above.

### Phylogenetic analysis

GAME31, GAME32, GAME32-like, 2-ODD34, and its homologous sequences from *Solanum* plants were identified using the BLASTP program (https://blast.ncbi.nlm.nih.gov/Blast.cgi). Sequence alignments were performed using ClustalOmega^43^. The Maximum Likelihood tree was inferred in MEGA6 using 1000 bootstrap replications^44^. Evolutionary distances are in units of number of amino acid substitutions per site. All positions containing gaps and missing data were eliminated. Amino acid sequences used in the phylogenetic analysis are provided in Supplementary Data 3.

### Hydroxytomatine purification for NMR analysis

Methanolic extracts from ~10 g *GAME31*-Ox transgenic plant leaves (#1, #2 are two independent *GAME31*-Ox transgenic lines, and from each transgenic line, ~5 g leaves were collected) were evaporated to dryness and re-dissolved in 1000 μl 80% methanol. The system consisted of an Agilent 1290 Infinity II UPLC system equipped with a quaternary pump, auto sampler, diode array detector, a Bruker/Spark Prospekt II LC-SPE system (Spark), and Impact HD UHR-QqTOF mass spectrometer (Bruker) connected via a Bruker NMR MS Interface (BNMI-HP)^45^. MS spectra, in positive mode, were acquired between *m*/*z* 50 and 1700. The calibration was done with a 10-mM sodium trifluoroacetate (Sigma-Aldrich) automatically, which is introduced at the beginning and the end of each chromatographic run. Separation was done on a XBridge LC column (BEH C18, 5 μm particle size, and 250 mm × 4.6 mm; Waters). The chromatographic conditions were as follows: a flow rate of 0.9 ml min^−1^ starting with a solvent composition of 87% A (5% acetonitrile (ACN) + 0.1% formic acid (FA)) and 13% B (100% ACN + 0.1% FA) with a linear gradient to 85% A at 30 min, followed by another linear gradient to 100% B at 32 min, 100% B held for 6 min followed by linear gradient to 87% A at 38.5, and held for 1.5 min for equilibration. Hydroxytomatine was collected on solid-phase extraction (SPE) cartridges in pre-set time windows and trapping was triggered by intensity threshold (hydroxytomatine (*m*/*z* 594.4—main fragment, threshold 510,000, time window 19–24 min). For this trapping process, a makeup flow of 2.5 ml min^−1^ water was added to the eluent before it passed through the SPE cartridges in order to increase the retention of analytes on the cartridges. For the trapping, 10 mm × 2 mm SPE cartridges filled with GP resin were used. Each cartridge was loaded five times with the same compound (i.e. hydroxytomatine), and 5–10 cartridges were used for trapping hydroxytomatine. Prior to NMR measurements, SPE cartridges were dried with a stream of nitrogen, and the fraction from each cartridge was eluted with a total of 150 μl deuterated methanol. The sample was eluted into 96-well plate. Eluents containing same compound were pooled, dried under stream of nitrogen, freeze-dried, resuspended in 200 μl of D_2_O, and freeze-dried again to remove traces of H_2_O. Dry compounds were dissolved in 60 μl 90% MeOD-d4 10% D_2_O with 0.01% addition of 3-propionic-2,2,3,3-d4 acid sodium salt (that was used as an internal chemical shift reference for ^1^H and ^13^C spectra) and transferred to a 1.7-mm NMR test tube.

### NMR methods

NMR spectra were recorded on a Bruker AVANCE NEO-600 NMR spectrometer equipped with a 5-mm TCI-xyz CryoProbe equipped with shielded gradient coils. All spectra were acquired at 293 K. NMR data of hydroxytomatine were recorded on 5 mm CryoProbe (using a special NMR spinner turbine to hold the 1.7 mm test tube in a 5-mm probe). ^1^H and ^13^C chemical shift assignment was based on different 1D and 2D NMR techniques (double quantum filtered correlation spectroscopy (DQF-COSY), total correlated spectroscopy (TOCSY), rotating-frame Overhauser spectroscopy, heteronuclear single quantum coherence (HSQC), heteronuclear multiple bond correlation (HMBC), and HSQC-TOCSY) all using pulsed field gradient selection, combining HMBC-derived information (Supplementary Table 1) with DQF-COSY and TOCSY correlations. No nuclear Overhauser effect interaction was observed for H-23 (using rotating Overhauser effect mixing times 100–250 ms), therefore it was difficult to determine the configuration of H-25. NMR spectra for hydroxytomatine is provided in Supplementary Figs. 33–40.

### Reporting summary

Further information on research design is available in the Nature Research Reporting Summary linked to this article.

## Supplementary information

Supplementary Information

Reporting Summary

Description of Additional Supplementary Files

Supplementary Data 1–4

Source Data

## Data Availability

Data supporting the findings of this work are available within the paper and its Supplementary Information files. A reporting summary for this article is available as a Supplementary Information file. The datasets generated and analyzed during the current study are available from the corresponding author upon request. The source data underlying Figs. 2c, 2d, 3a, 3b, and 4a, and Supplementary Figs 2b, 3b, 4b, 5b, 6b, 15, 16, 18, and 22 are provided as a Source Data File.
