# Supplementary material for: Pathways to defense metabolites and evading fruit bitterness in genus Solanum evolved through 2-oxoglutarate-dependent dioxygenases
Source: Nat Commun. 2019 Nov 14;10:5169. doi: 10.1038/s41467-019-13211-4 (PMC6856131; doi:10.1038/s41467-019-13211-4)
Supplement: Supplementary file 1 — Supplementary Information [file 41467_2019_13211_MOESM1_ESM.pdf]

Pathways to defense metabolites and evading fruit bitterness in genus *Solanum* evolved through 2-oxoglutarate-dependent dioxygenases

Cárdenas *et al.*

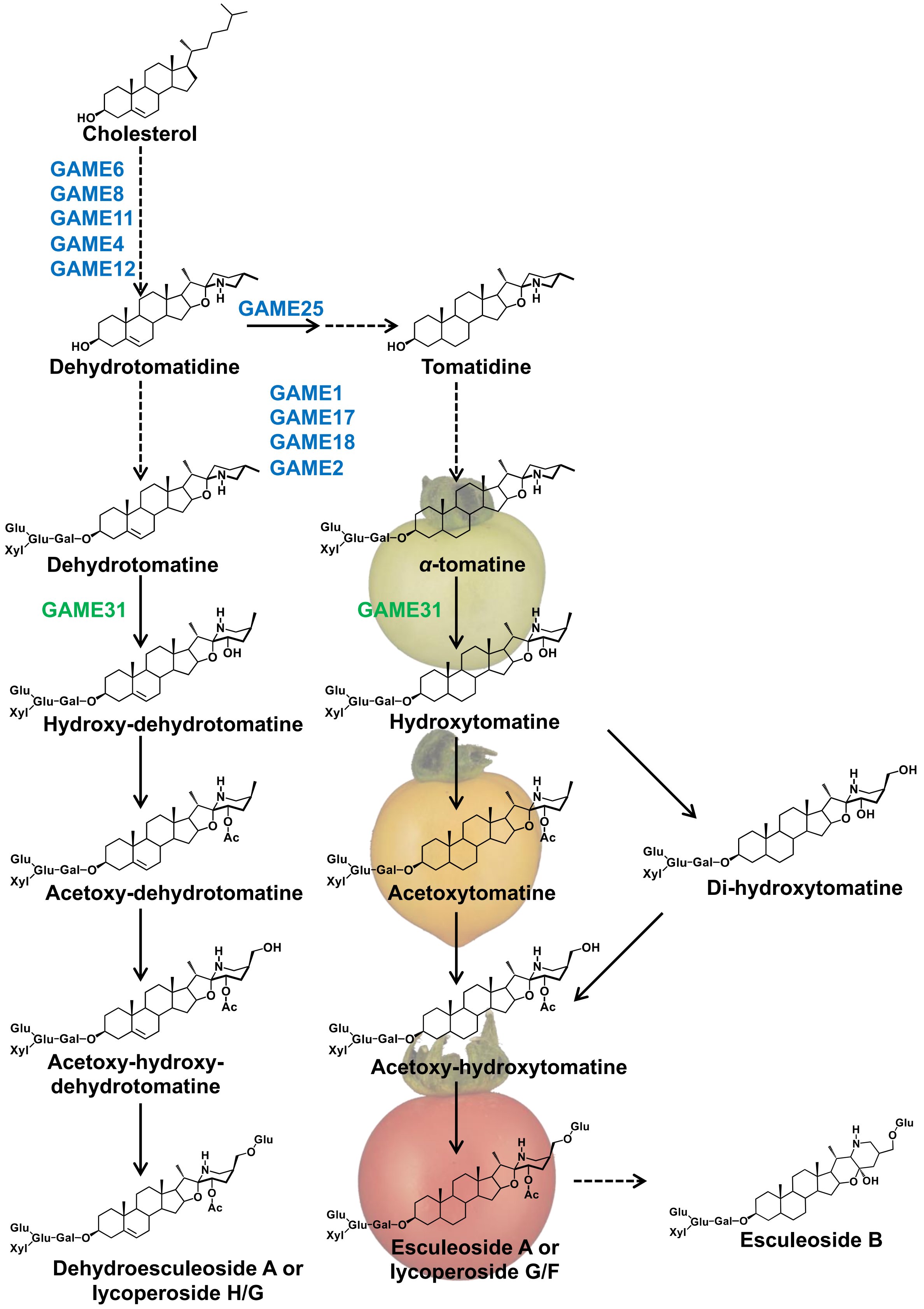

Cholesterol
GAME6
GAME8
GAME11
GAME4
GAME12
Dehydrotomatidine
GAME25
Tomatidine
GAME1
GAME17
GAME18
GAME2
Dehydrotomatine
α-tomatine
GAME31
GAME31
Hydroxy-dehydrotomatine
Hydroxytomatine
Acetoxy-dehydrotomatine
Acetoxytomatine
Di-hydroxytomatine
Acetoxy-hydroxy-dehydrotomatine
Acetoxy-hydroxytomatine
Dehydroesculeoside A or lycoperoside H/G
Esculeoside A or lycoperoside G/F
Esculeoside B

Supplementary Figure 1. Steroidal glycoalkaloids metabolism during tomato fruit development and ripening. Cholesterol serves as a precursor for steroidal glycoalkaloid (SGA) biosynthesis. In tomato, cholesterol is further modified by GLYCOALKALOID METABOLISM (GAME) enzymes (in blue) through hydroxylation, oxidation, transamination and glycosylation to generate core SGAs. *α*-tomatine and dehydrotomatine are the main SGAs accumulating in the green fruit. Subsequently, hydroxy- and/or acetoxy-derivatives of these SGAs accumulate at the breaker fruit stage. In the red ripe tomato fruit, esculeoside A is the most predominant SGA. This ripening associated chemical shift from *α*-tomatine (green fruit) towards esculeoside A (red fruit) involves several modification steps including hydroxylation, acetylation and glycosylation. The enzymatic conversion responsible for any of these steps remains elusive. Following this study, we show that GAME31 (in green) catalyzes hydroxylation of *α*-tomatine, the first committed step in esculeoside A biosynthesis. Dashed arrows represent multiple biosynthetic reactions whereas solid arrows represent a single step. Ac: Acetoxy; Glu: Glucose; Gal: Galactose; Xyl: Xylose and Rha: Rhamnose.

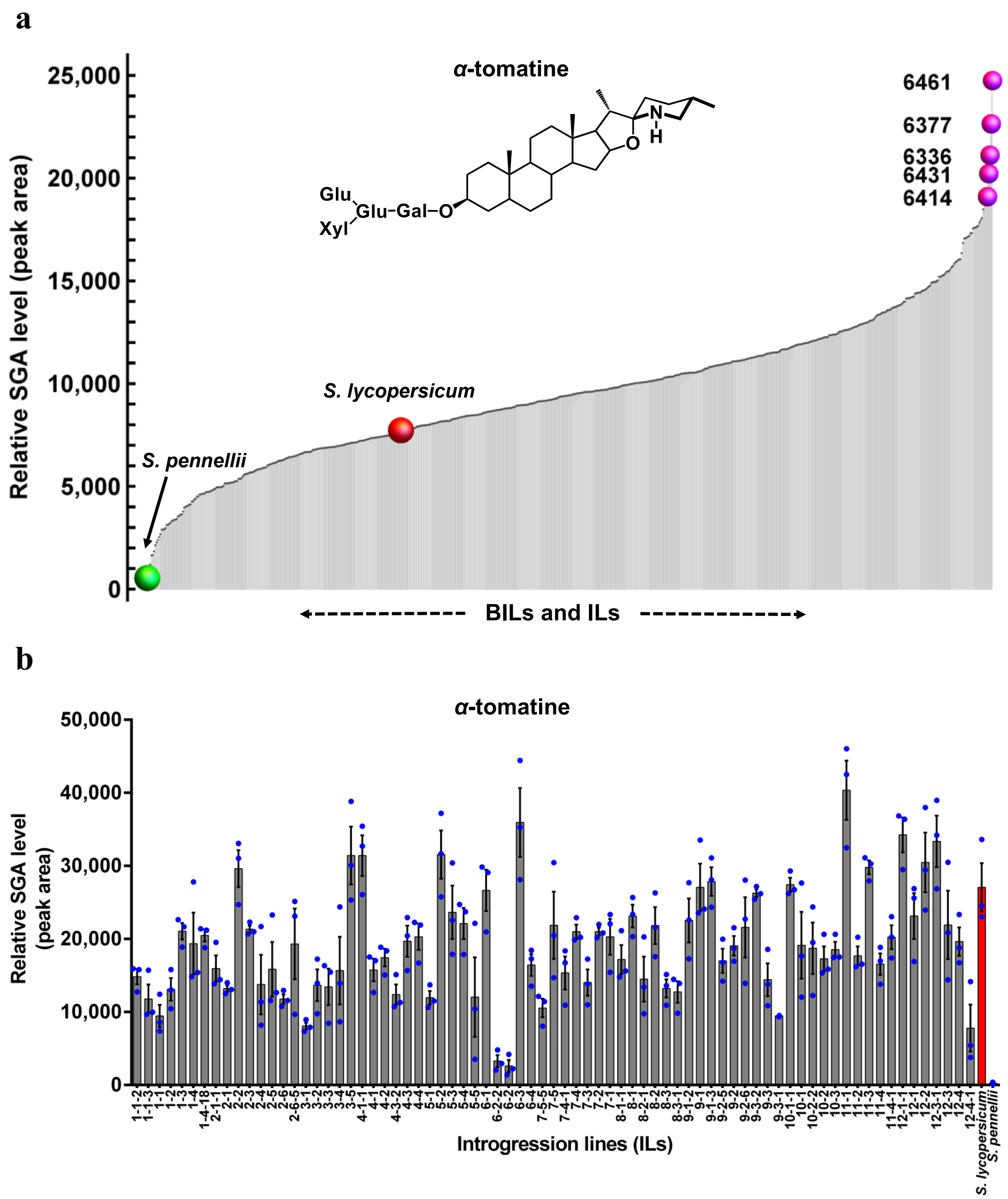

Supplementary Figure 2. LC-MS based screening of *α*-tomatine in backcross inbred lines and introgression lines for QTL mapping analysis. (a) Levels of *α*-tomatine in leaf tissue. Respective SGA content was determined by the leaf-dipping method (n=1) of Backcross inbred lines (BILs) and Introgression lines (ILs) population (total 671 lines). SGA level presented in the plot was arranged in ascending order. Top five BILs/ILs showing highest *α*-tomatine content are marked by a pink spot along with the parental lines (*S. lycopersicum* and *S. pennellii* in red

and green spots, respectively). Details of SGAs levels across the BILs and ILs population are provided in Supplementary Data 1. **(b)** *α*-tomatine level in 77 core ILs population including parental lines. SGA content was determined from ground leaf tissue extracts (n=3). The peak areas were determined using the TargetLynx software. The values in panel b represent the means of three biological replicates ± standard error mean (per IL line). The source data of Supplementary Figure 2b are provided as a Source Data file.

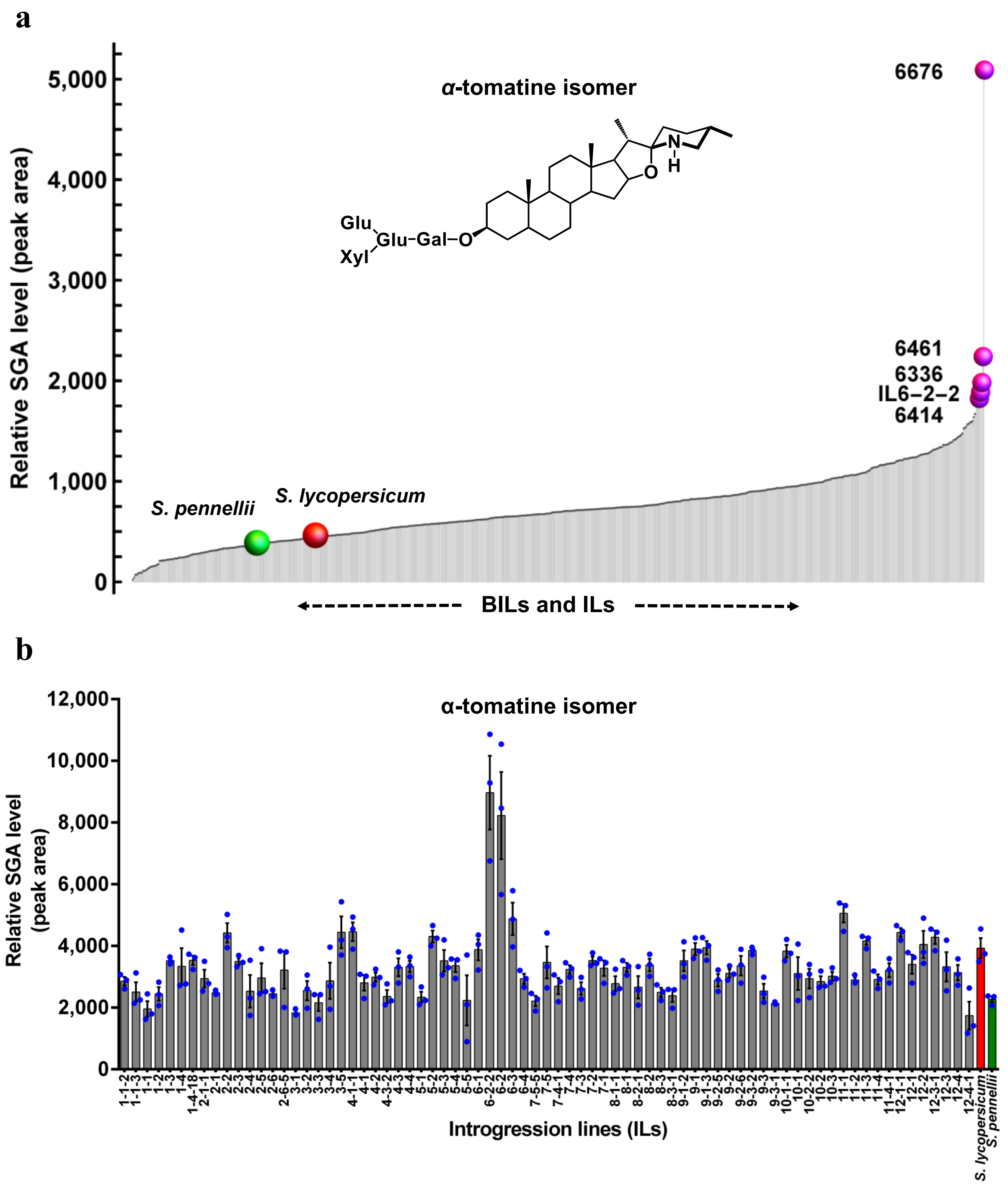

Supplementary Figure 3. LC-MS based screening of *α*-tomatine isomer in backcross inbred lines and introgression lines for QTL mapping analysis. (a) Levels of *α*-tomatine isomer in leaf tissue. Respective SGA content was determined by the leaf-dipping method (n=1) of Backcross inbred lines (BILs) and Introgression lines (ILs) population (total 671 lines). SGA level presented in the plot was arranged in ascending order. Top five BILs/ILs showing highest *α*-tomatine isomer content are marked by a pink spot along with the parental lines (*S. lycopersicum* and *S. pennellii*

in red and green spots, respectively). Details of SGAs levels across the BILs and ILs population are provided in Supplementary Data 1. **(b)** *α*-tomatine isomer level in 77 core ILs population including parental lines. SGA content was determined from ground leaf tissue extracts (n=3). The peak areas were determined using the TargetLynx program. The values in panel b represent the means of three biological replicates ± standard error mean (per IL line). The source data of Supplementary Figure 3b are provided as a Source Data file.

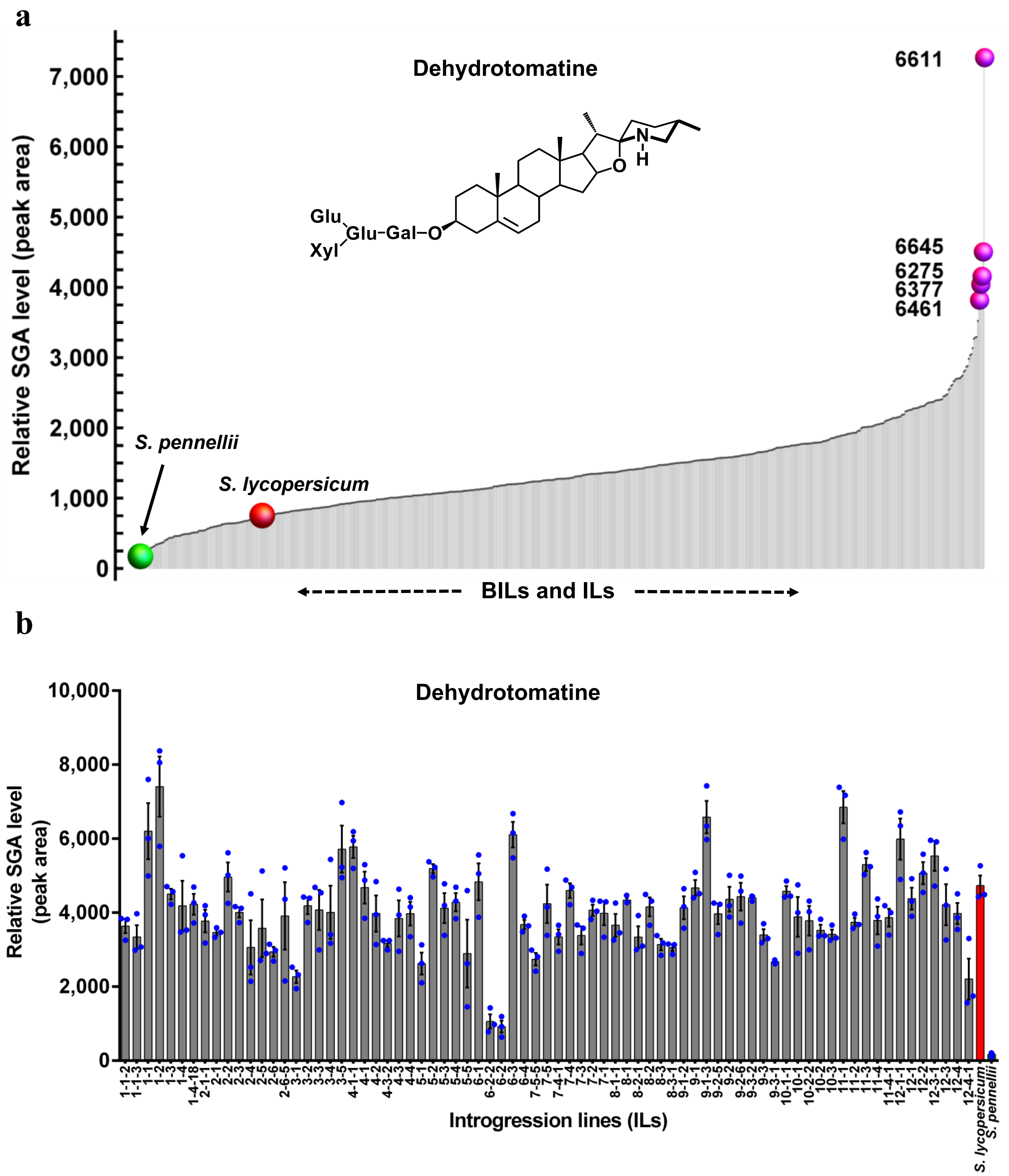

Supplementary Figure 4. LC-MS based screening of dehydrotomatine in backcross inbred lines and introgression lines for QTL mapping analysis. (a) Levels of dehydrotomatine in leaf tissue. Respective SGA content was determined by the leaf-dipping method (n=1) of Backcross inbred lines (BILs) and Introgression lines (ILs) population (total 671 lines). SGA level presented in the plot was arranged in ascending order. Top five BILs/ILs showing highest dehydrotomatine content are marked by a pink spot along with the parental lines (*S. lycopersicum* and *S. pennellii*

in red and green spots, respectively). Details of SGAs levels across the BILs and ILs population are provided in Supplementary Data 1. **(b)** Dehydrotomatine level in 77 core ILs population including parental lines. SGA content was determined from ground leaf tissue extracts (n=3). The peak areas were determined using the TargetLynx software. The values in panel b represent the means of three biological replicates ± standard error mean (per IL line). The source data of Supplementary Figure 4b are provided as a Source Data file.

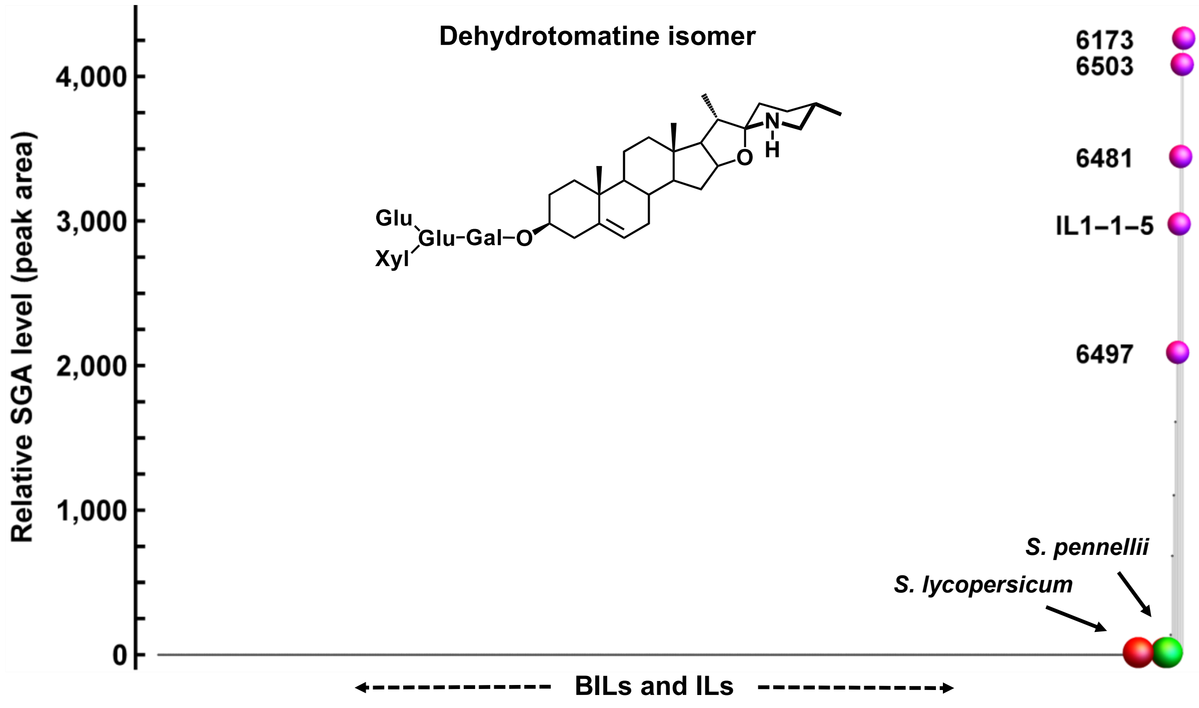

b

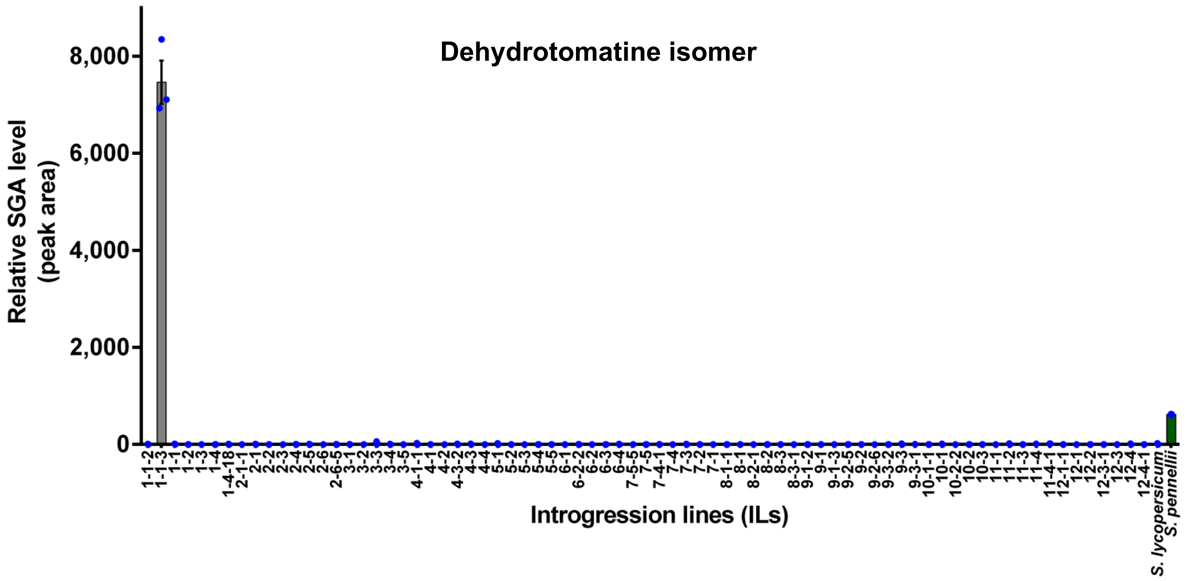

Supplementary Figure 5. LC-MS based screening of dehydrotomatine isomer in backcross inbred lines and introgression lines for QTL mapping analysis. (a) Levels of dehydrotomatine isomer in leaf tissue. Respective SGA content was determined by the leaf-dipping method (n=1) of Backcross inbred lines (BILs) and Introgression lines (ILs) population (total 671 lines). SGA level presented in the plot was arranged in ascending order. Top five BILs/ILs showing highest dehydrotomatine isomer content are marked by a pink spot along with the parental lines (*S.*

lycopersicum and *S. pennellii* in red and green spots, respectively). Details of SGAs levels across the BILs and ILs population are provided in Supplementary Data 1. **(b)** Dehydrotomatine isomer level in 77 core ILs population including parental lines. SGA content was determined from ground leaf tissue extracts (n=3). The peak areas were determined using the TargetLynx software. The values in panel b represent the means of three biological replicates ± standard error mean (per IL line). The source data of Supplementary Figure 5b are provided as a Source Data file.

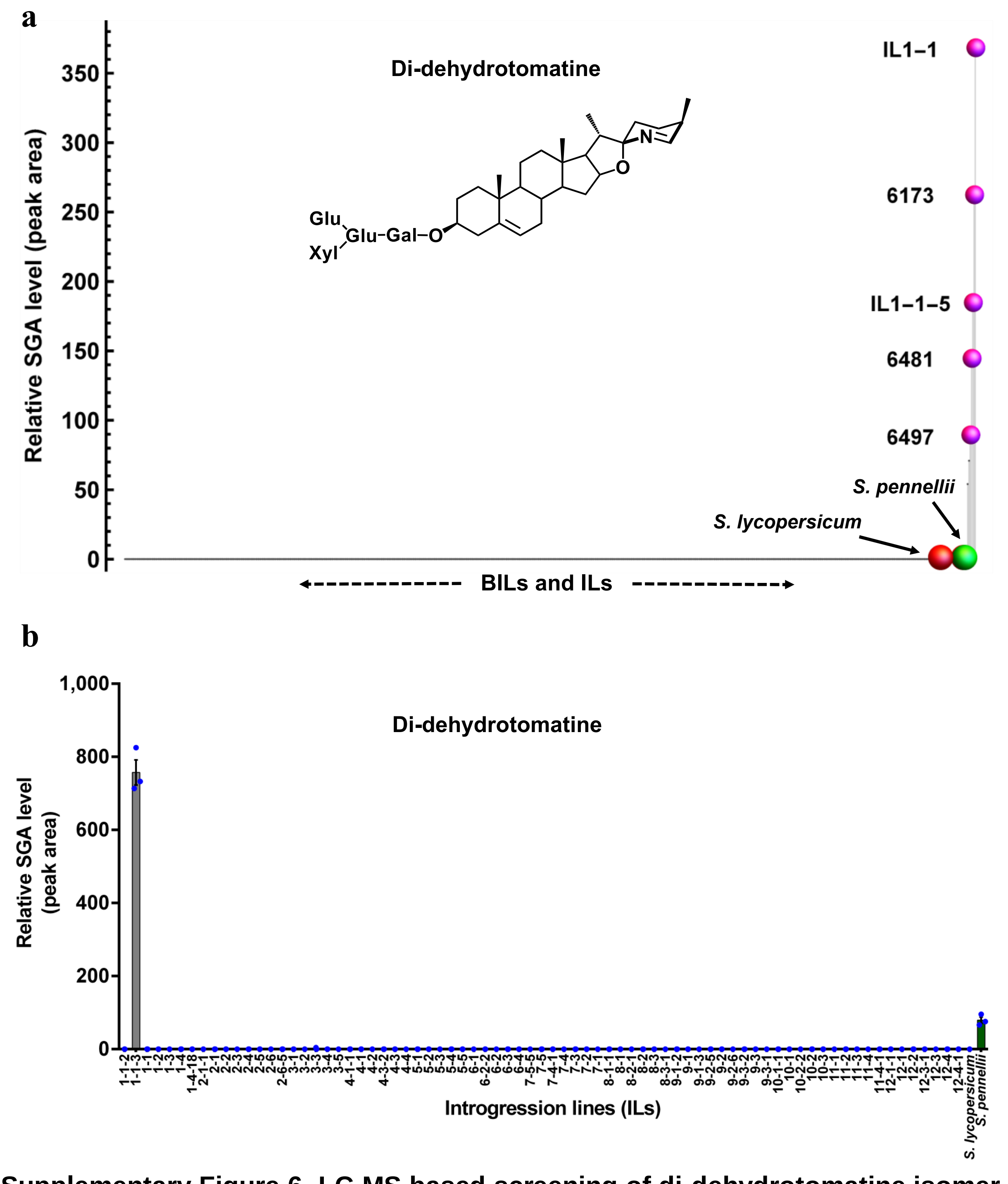

Supplementary Figure 6. LC-MS based screening of di-dehydrotomatine isomer in backcross inbred lines and introgression lines for QTL mapping analysis. (a) Levels of di-dehydrotomatine isomer in leaf tissue. Respective SGA content was determined by the leaf-dipping method (n=1) of Backcross inbred lines (BILs) and Introgression lines (ILs) population (total 671 lines). SGA level presented in the plot was arranged in ascending order. Top five BILs/ILs showing highest di-dehydrotomatine isomer content are marked by a pink spot along with the parental

lines (*S. lycopersicum* and *S. pennellii* in red and green spots, respectively). Details of SGAs levels across the BILs and ILs population are provided in Supplementary Data 1. **(b)** Di-dehydrotomatine isomer level in 77 core ILs population including parental lines. SGA content was determined from ground leaf tissue extracts (n=3). The peak areas were determined using the TargetLynx software. The values in panel b represent the means of three biological replicates ± standard error mean (per IL line). The source data of Supplementary Figure 6b are provided as a Source Data file.

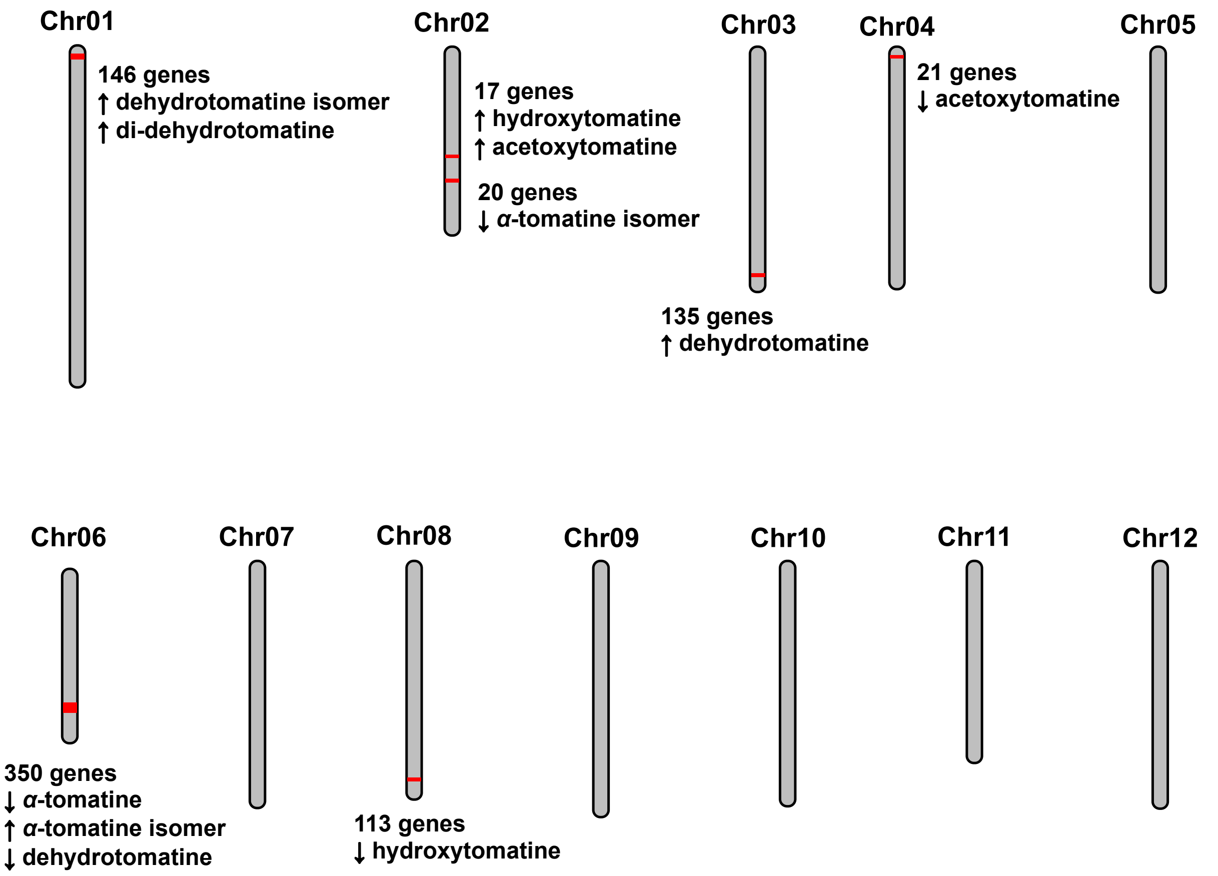

Supplementary Figure 7. Schematic representation of chromosomal regions in the tomato genome displaying mapping bins associated with QTLs linked to SGAs content variation in the BILs and ILs population. To identify candidate genes associated with SGAs modifications, we exploited the ILs (Introgression lines) and BILs (Backcross inbred lines) tomato population to map the QTLs (Quantitative Trait Loci) influencing specific SGAs composition. In our initial approach, we measured levels of seven different SGA in a tomato ILs and BILs population (671 lines). Using the SGAs content information from the screened lines, we identified the chromosomal regions (marked in red) in tomato lines that are linked to the variation of each SGA. In this study we focused our analysis on chromosome 2 region QTL containing 17 genes that was associated with increase in hydroxytomatine and acetoxytomatine SGAs. Chromosomes 5, 7, 9, 10, 11 and 12 did not show any QTL region related to SGA metabolism

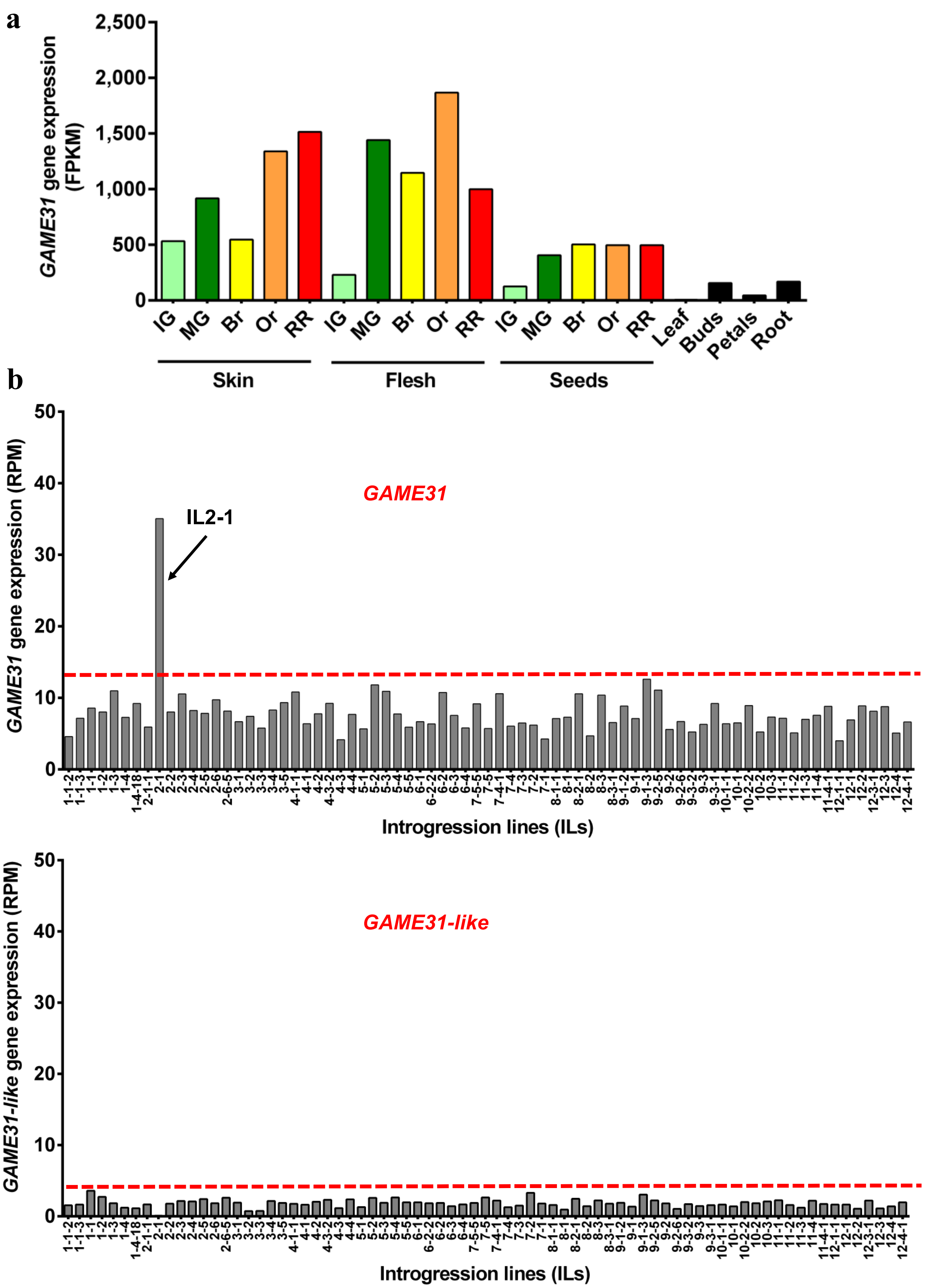

Supplementary Figure 8. *GAME31* and *GAME31-like* candidate gene expression. (a) Normalized *GAME31* (Solyc02g062460) expression level in tomato (*cv.* Micro Tom) tissue types and developmental stages (RNA-seq expression

data)[26]. Expression of the *GAME31-like* gene (Solyc02g062500) was not detected in the same transcriptome dataset. Fruit developmental stages- IG: immature green; MG: mature green; Br: breaker; Or: orange; RR: red ripe. FPKM: Fragments Per Kilobase of transcript per Million mapped reads. **(b)** Normalized expression profile of *GAME31* and *GAME31-like* genes in the vegetative apex of the Introgression Lines (ILs) (RNA-Seq data)[27]. *GAME31* gene showed differential expression in the IL2-1 line across the entire ILs population. *GAME31-like* gene expression did not change across the population. RPM: Reads Per Million. Red dotted line shows maximum upper cut-off expression value for candidate gene across ILs population except IL2-1 line.

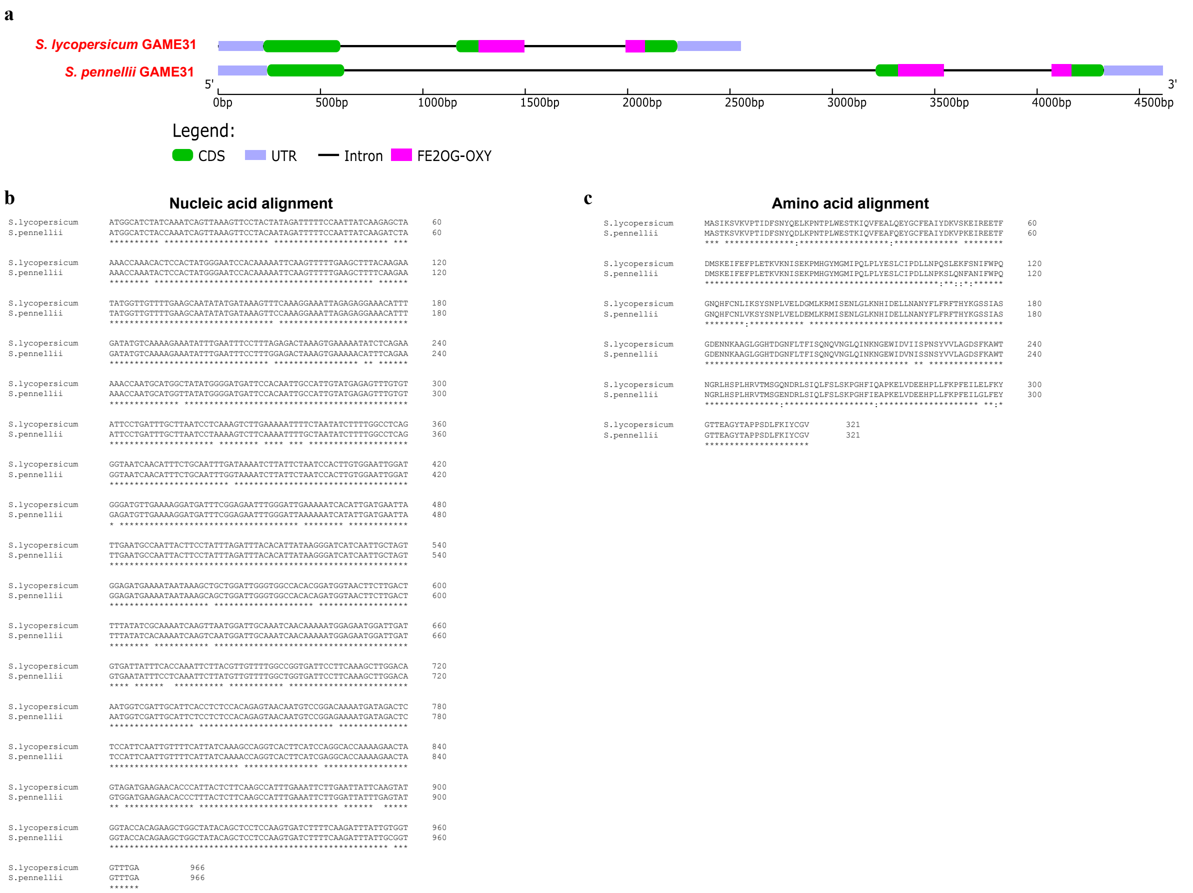

Supplementary Figure 9. Comparison of GAME31 between *S. lycopersicum* and *S. pennellii*. (a) *GAME31* gene structure between *S. lycopersicum* and *S. pennellii*. **(b, c)** Nucleic acid (b), amino acid (c) sequences alignment between *S. lycopersicum GAME31* and *S. pennellii GAME31* variants.

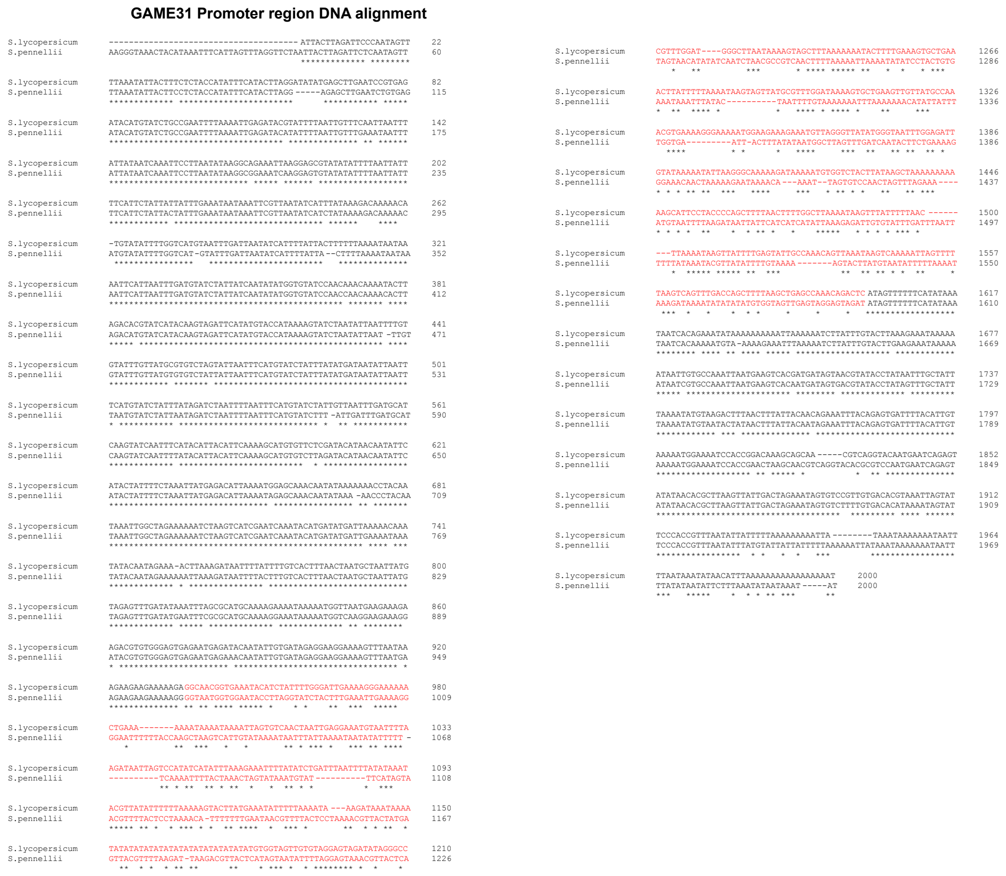

Supplementary Figure 10. Promoter region sequences alignment between *S. lycopersicum GAME31* and *S. pennellii GAME31* variants. About 2000 base pairs upstream DNA region was used for alignment. Significant differences in *GAME31* promoter sequences between two variants is shown in red.

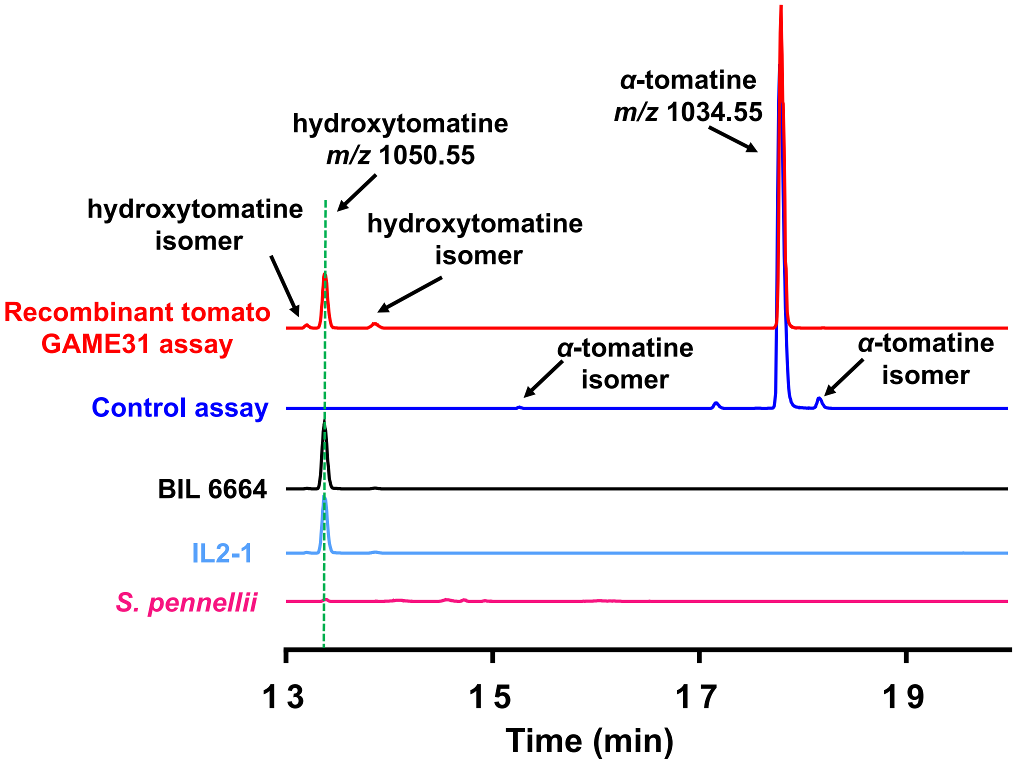

Supplementary Figure 11. Aligned extracted ion chromatograms for hydroxytomatine SGA comparison. Selected BIL 6664 (leaves), IL2-1 line (leaves), *S. pennellii* (leaves), recombinant tomato GAME31 enzyme assay reaction and control assay reaction samples were used for alignment. Hydroxytomatine is a minor SGA in *S. pennellii* leaves. However, leaves of selected BILs (e.g. 6664) and ILs (e.g. IL2-1) accumulate hydroxytomatine in higher quantities. Recombinant GAME31 enzyme produces same hydroxytomatine peak as found in leaves of BIL 6664 and IL2-1 line. LC-MS (long run 40 min) was used for metabolite analysis. Control assay was performed using protein extracts of *E. coli* cells transformed with empty vector. *m/z*: Mass to charge

a

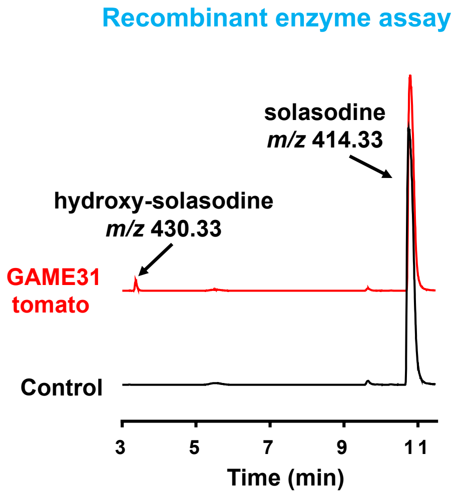

b

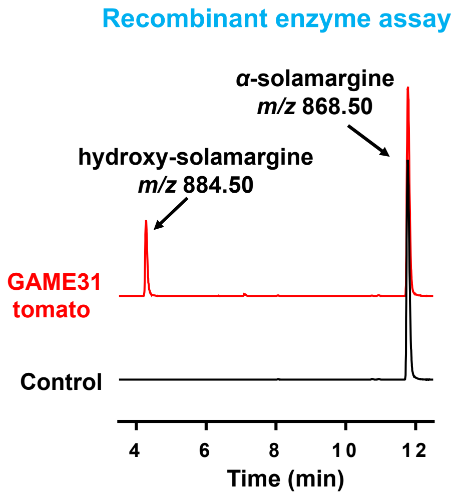

Supplementary Figure 12. LC-MS analysis of recombinant tomato GAME31 enzyme assay performed with eggplant substrates. (a, b) The tomato recombinant GAME31 enzyme produced in *E. coli* cells catalyzes hydroxylation of solasodine (a) and *α*-solamargine (b) to form hydroxy-solasodine and hydroxy-solamargine, respectively (shown in red). Control reaction with the respective substrate (shown in black) was carried out using the protein extracts from *E. coli* cells transformed with empty vector. Mass to charge (*m/z*) is shown for substrates and assay products. MS-MS analysis and identification of hydroxy-solasodine and hydroxy-solamargine is provided in Supplementary Fig. 25 and 26.

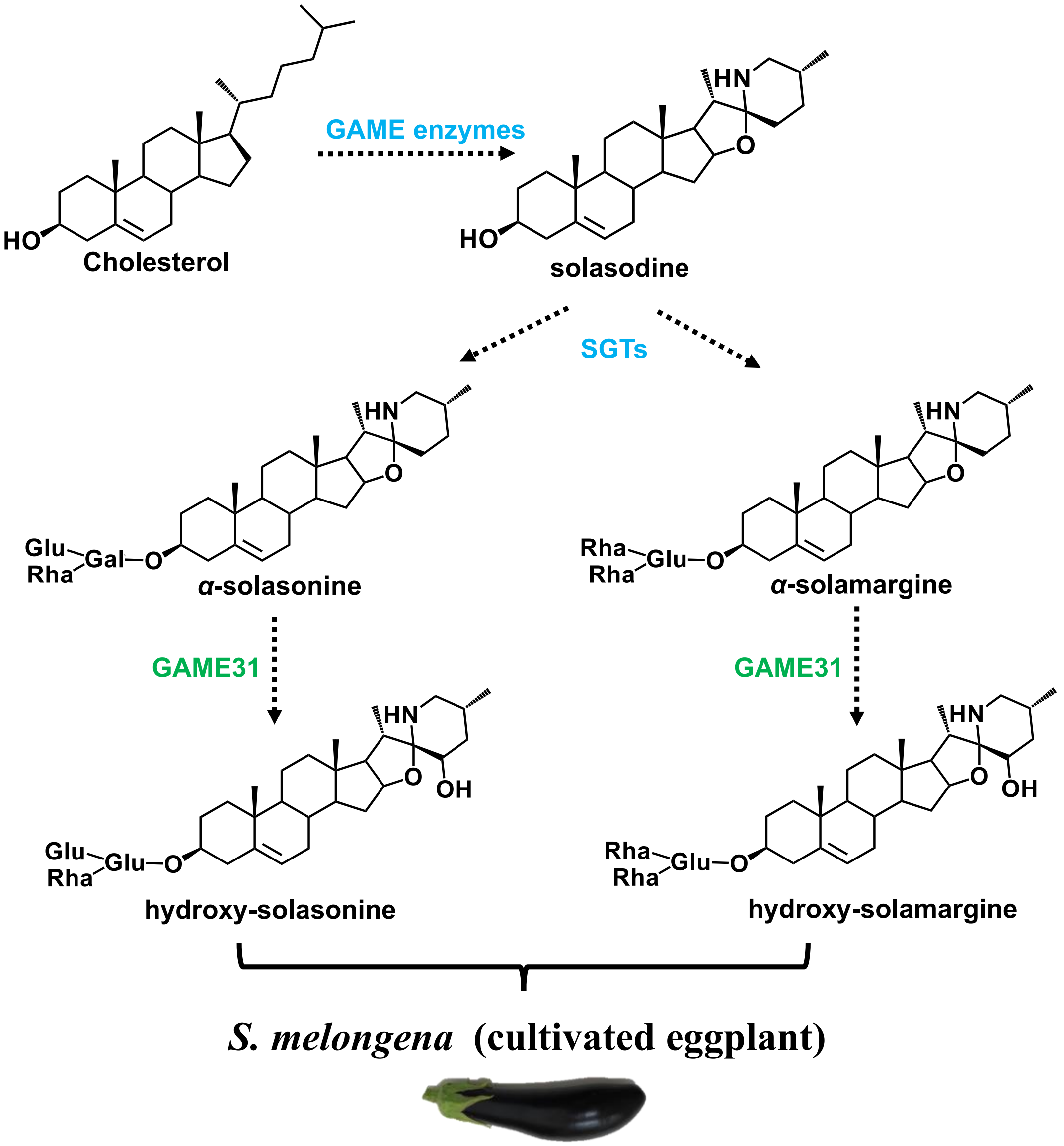

Supplementary Figure 13. Biosynthetic pathway for SGAs in cultivated eggplant. In cultivated eggplant, *α*-solasonine and *α*-solamargine are the main SGAs formed through the action of GLYCOALKALOID METABOLISM (GAME) and STEROL ALKALOID GLYCOSYLTRANSFERASE (SGT) enzymes (blue). The cultivated eggplant GAME31 enzyme characterized in this study is marked in green. Dashed and solid arrows indicate multiple or single enzymatic steps in the pathway, respectively. Glu: Glucose; Rha: Rhamnose.

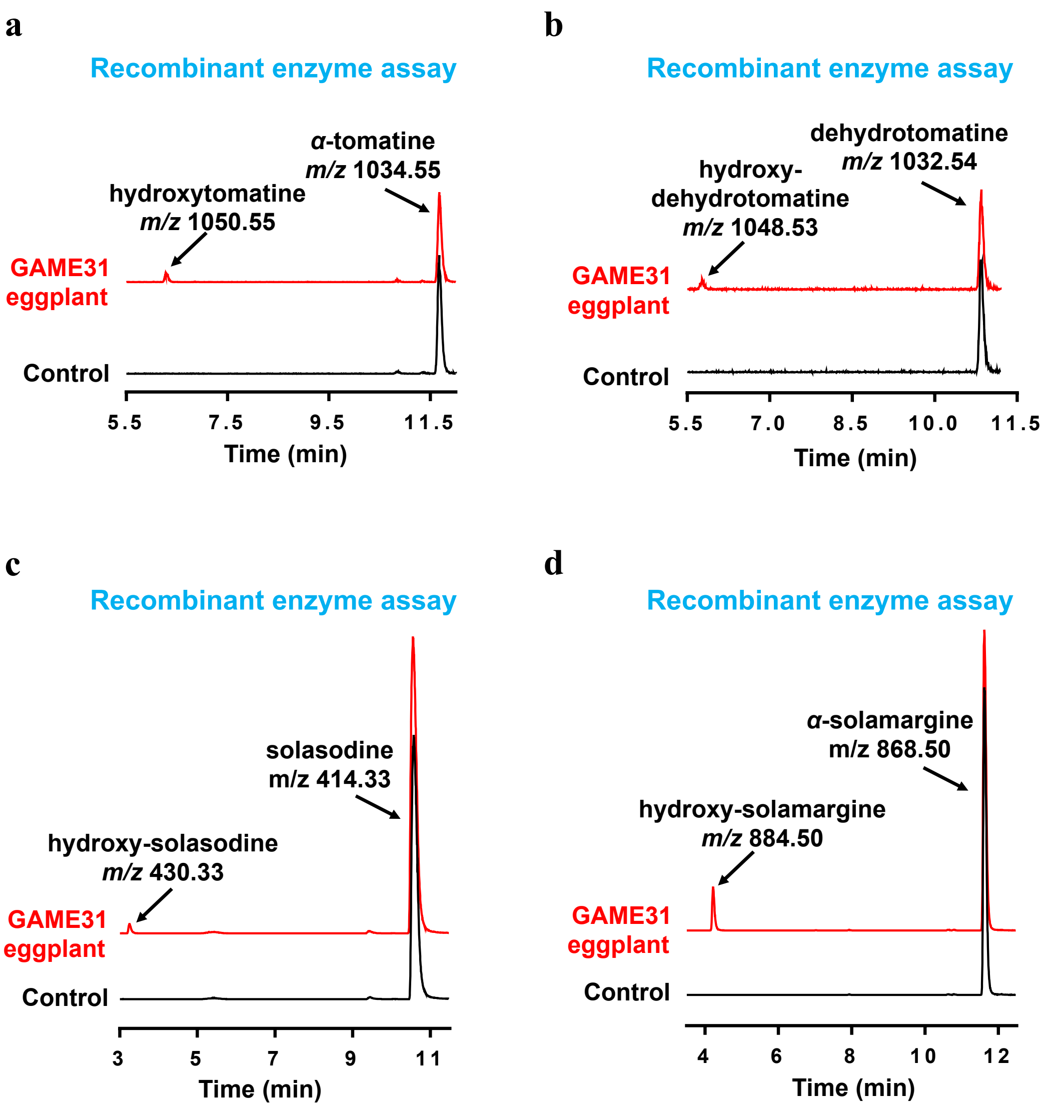

Supplementary Figure 14. Characterization of the recombinant eggplant GAME31 by *in vitro* enzyme assays. (a-d) Overlay of extracted ion chromatograms of hydroxylated products and substrates in eggplant GAME31 assay and control reaction respectively. Red: Recombinant GAME31 enzyme reaction with respective substrates; *α*-tomatine (a), dehydrotomatine (b), solasodine (c) and *α*-solamargine (d). Black: control reaction with respective substrates (names as above) using empty vector-transformed *E. coli* protein extracts. Mass to charge (*m/z*) is shown for substrates and assay products. The recombinant eggplant GAME31 enzyme was produced in *E. coli* cells. LC-MS was used for enzyme assay products analysis. MS-MS analysis and putative identification of hydroxylated compounds (GAME31 assay products) are provided in Supplementary Figs. 23-26.

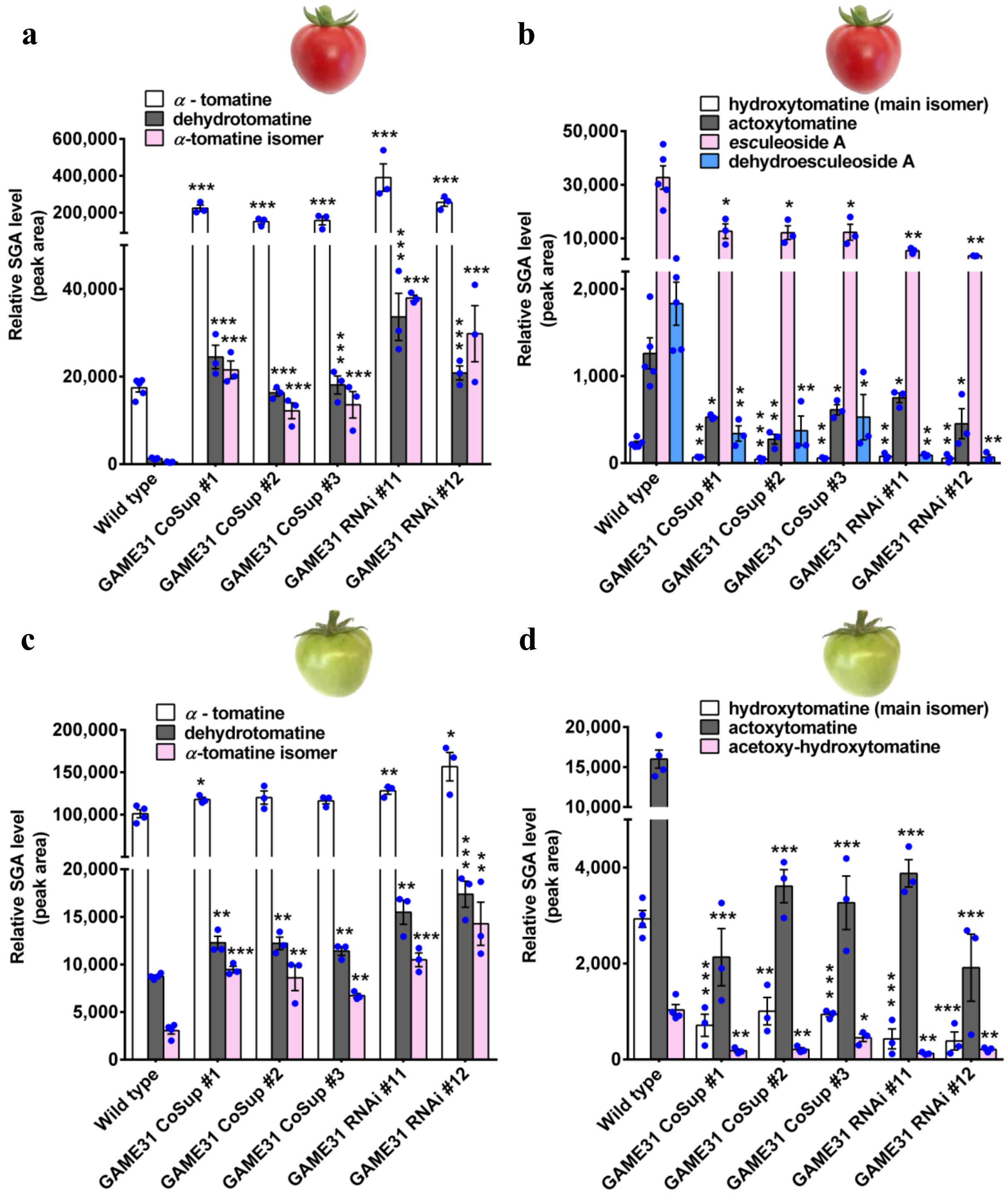

Supplementary Figure 15. Silencing of *GAME31* has a major impact on SGAs composition in developing tomato fruit. (a, b) Levels of SGAs produced upstream of the GAME31 reaction step (a), and downstream of the GAME31 reaction step (b) in red fruit of *GAME31*-silenced tomato lines [by RNAi interference (RNAi) and co-suppression (CoSup)]. **(c, d)** Levels of upstream (c) and downstream (d) SGAs in green stage tomato fruit following *GAME31*-silencing as compared to wild type (non-transformed) green fruit. Silencing of *GAME31* resulted in significant accumulation of

precursor SGAs (e.g. *α*-tomatine isomers and dehydrotomatine), with concomitant reduction in downstream SGAs (e.g. hydroxytomatine, acetoxytomatine, esculeoside A) in tomato fruit tissues. See Supplementary Fig. 1 for a detailed tomato SGA pathway. Lines #11 and #12 are two independent *GAME31*-RNAi transgenic lines, while lines #1, #2, #3 are three independent *GAME31*-Co-supression transgenic lines. Values indicate means ± standard error mean (n=4 for wild type and n=3 for individual transgenic lines genotype). Asterisks indicate significant changes compared to wild type samples as calculated by a Student's t-test (*P-value < 0.05; **P-value < 0.01; ***P-value < 0.001). LC-MS was used for targeted SGAs analysis. Source data are provided as a Source Data file.

a

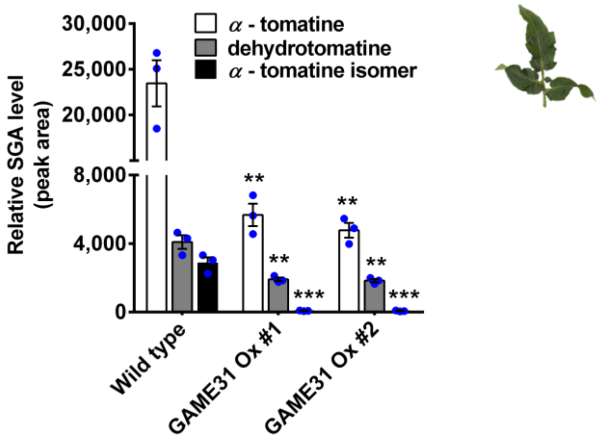

b

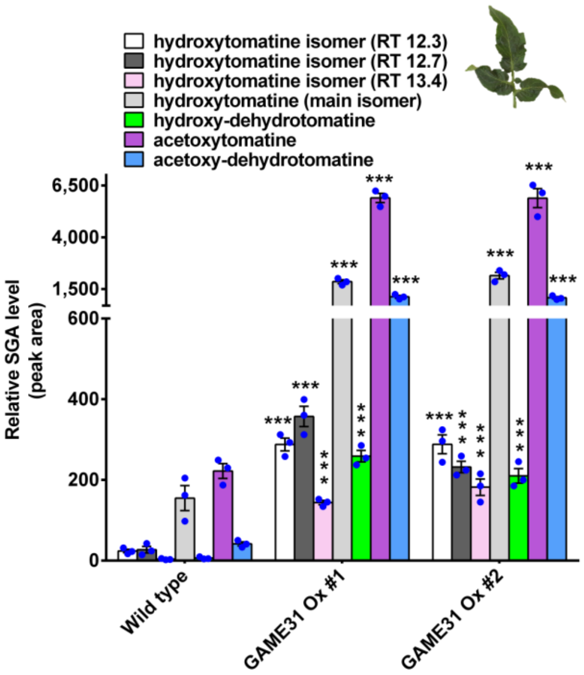

Supplementary Figure 16. Overexpression of the tomato *GAME31* in tomato leads to accumulation of downstream hydroxy- and acetoxy- SGA derivatives and reduces its precursor SGAs in leaf tissues. (a) Levels of *α*-tomatine isomers and dehydrotomatine SGAs in leaves of *GAME31*-overexpression (*GAME31*-Ox) tomato lines as compared to wild type ones. **(b)** Levels of hydroxy- and acetoxy-SGAs (downstream SGAs derived from *α*-tomatine and dehydrotomatine) in leaves of *GAME31*-Ox lines compared to wild type. See Supplementary Fig. 1 for SGA pathway steps and intermediates. Lines #1 and #2 are two independent *GAME31*-Ox transgenic lines. The values indicate means of biological replicates ± standard error mean from wild-type or an individual transgenic line (n=3). Asterisks indicate significant changes compared to wild type (non-transformed) samples as calculated by a Student's t-test (*P-value < 0.05; **P-value < 0.01; ***P-value < 0.001). LC-MS was used for targeted SGA profiling. Source data are provided as a Source Data file.

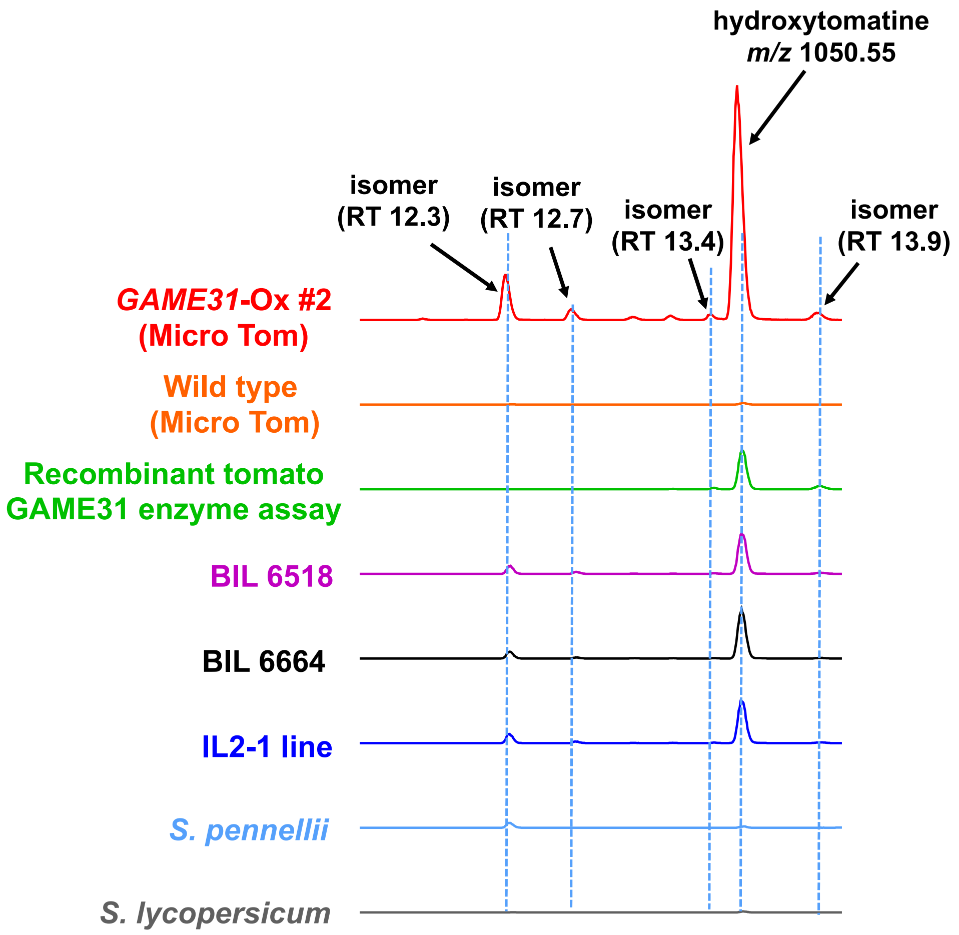

Supplementary Figure 17. Aligned extracted ion chromatograms showing hydroxytomatine and its isomer levels. Selected BILs, IL2-1 line, parents (*S. lycopersicum cv.* M82 and *S. pennellii*), tomato GAME31 enzyme assay and *GAME31-Ox* lines (#2 is shown here as representative transgenic line) were used for alignment. Hydroxytomatine is minor SGA in cultivated tomato (*S. lycopersicum cv.* M82 and *cv.* Micro Tom) and *S. pennellii* leaves. However, leaves of selected BILs (e.g. 6518 or 6664) and IL2-1 line show higher accumulation of hydroxytomatine (major peak) and its isomers (minor peaks). Recombinant tomato GAME31 enzyme assay and leaves of *GAME31*-Ox lines produce the same hydroxytomatine and its isomers that are detected in leaves of selected BILs and IL2-1 line. Mass to charge (*m/z*) is shown for hydroxytomatine compound.

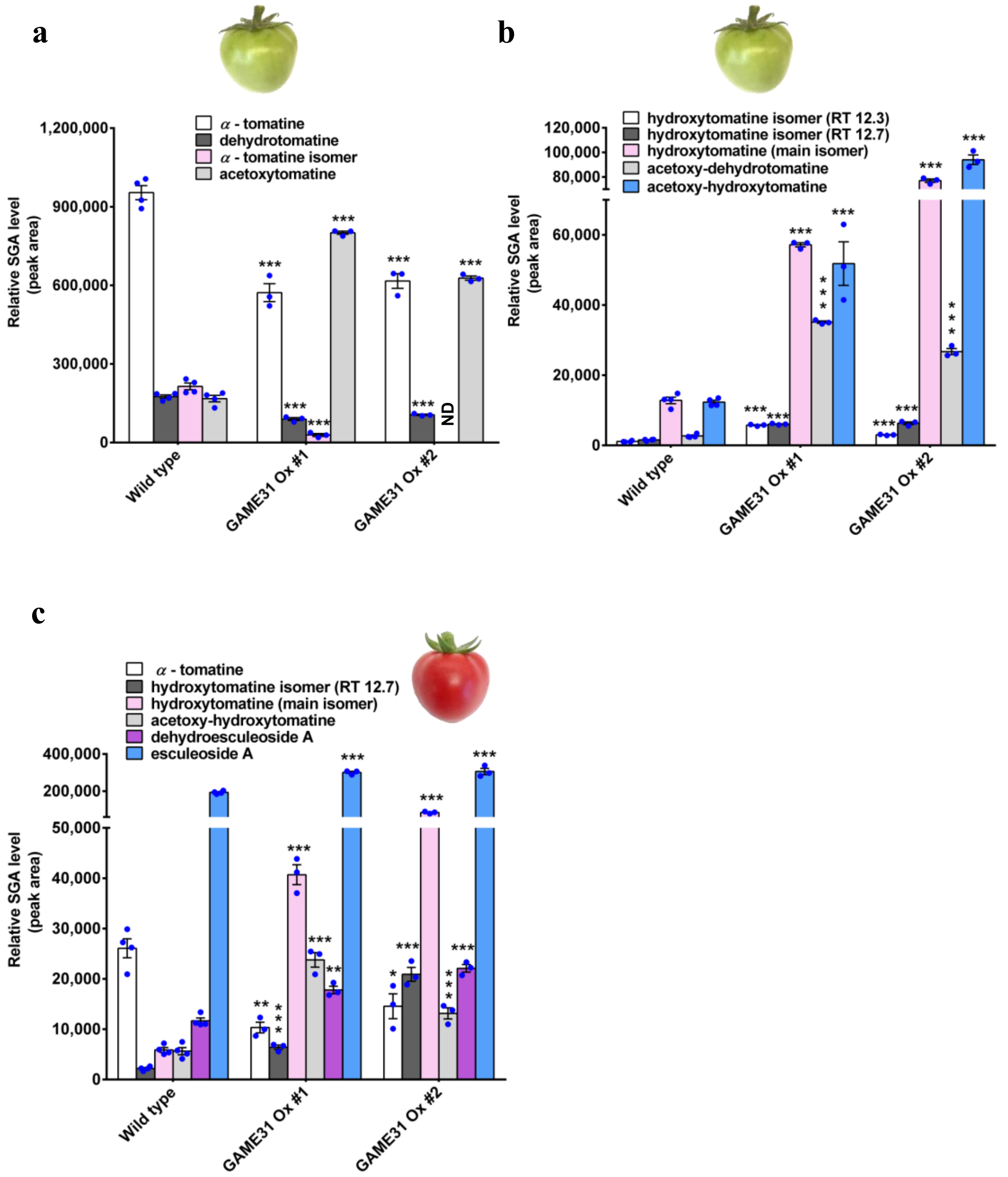

Supplementary Figure 18. Levels of *α*-tomatine and downstream SGAs detected in green and red fruit of *GAME31*-Ox lines as compared to wild-type fruit. (a, b) green fruit and **(c)** red fruit SGAs in *GAME31*-Ox lines. See Supplementary Fig. 1 for SGA pathway steps and intermediates. Lines #1 and #2 are two independent *GAME31*-Ox transgenic lines. The values indicate means of biological replicates ± standard error mean from wild-type (n≥3) or an individual transgenic line (n=3). Asterisks indicate significant changes compared to wild type (non-transformed) samples as calculated by a Student's t-test (*P-value < 0.05; **P-value < 0.01; ***P-value < 0.001). LC-MS was used for targeted SGA profiling. ND: Not Detected. Source data are provided as a Source Data file.

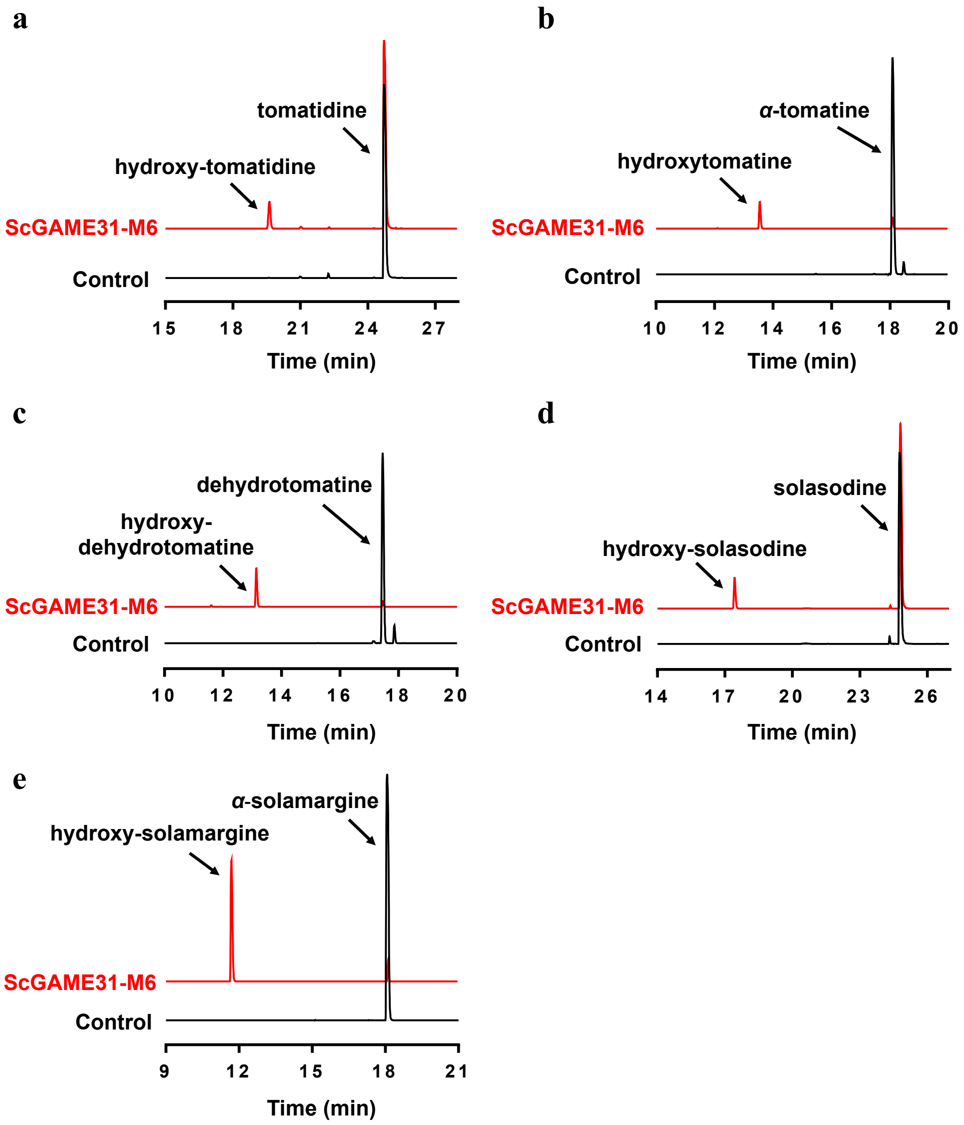

Supplementary Figure 19. Activity of GAME31 recombinant enzyme from *S. chacoense* accession M6. The *GAME31* gene was isolated from the M6 accession of *S. chacoense* (*Sc*) (termed as *ScGAME31-M6*). Furthermore, *ScGAME31-M6* was cloned and expressed in *E. coli* cells. **(a-e)** Aligned chromatograms (extracted ion) of hydroxylated products and substrates detected in the ScGAME31-M6 assay and control reaction. Red: Recombinant ScGAME31-M6 (a-e) enzyme reaction with respective substrates; tomatidine (a) *α*-tomatine (b), dehydrotomatine (c), solasodine (d) and *α*-solamargine (e). Black: control reactions using protein extracts from empty vector transformed *E. coli* cells with the respective substrates. Enzyme assays analysis was carried out by LC-MS. MS-MS analysis and putative identification of hydroxylated products are provided in Supplementary Figs. 23-27. Refer Supplementary Table 1 for NMR based assignment of hydroxytomatine compound.

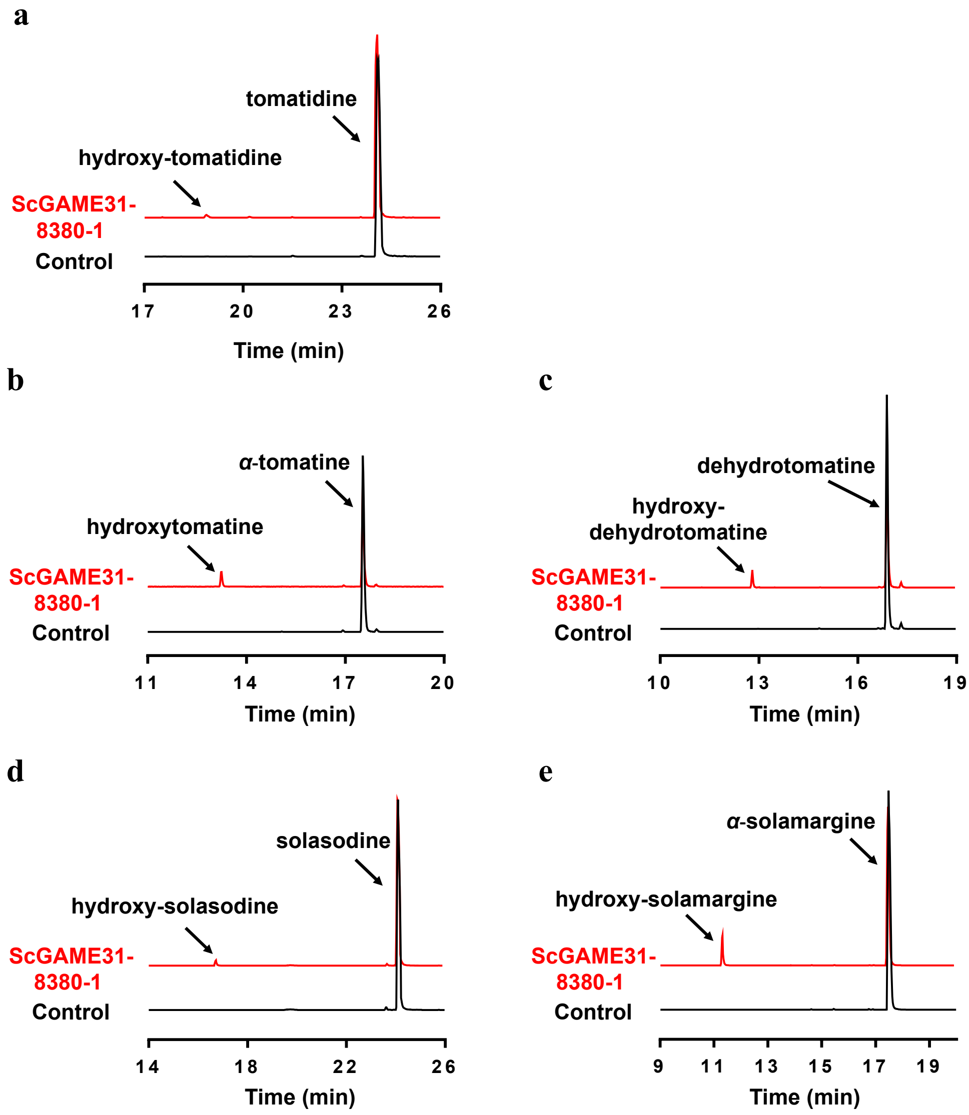

Supplementary Figure 20. Activity of GAME31 recombinant enzyme from *S. chacoense* accession 8380-1. The *GAME31* gene was isolated from 8380-1 accession of *S. chacoense* (*Sc*) (annotated as *ScGAME31-8380-1*) and was cloned, expressed in *E. coli* cells. **(a-e)** Aligned chromatograms (extracted ion) of hydroxylated products and substrates in the ScGAME31-8380-1 enzyme assay and control reaction. Red: Recombinant ScGAME31-8380-1 (a-e) enzyme reaction with respective substrates; tomatidine (a) *α*-tomatine (b), dehydrotomatine (c), solasodine (d) and *α*-solamargine (e). Black: control reactions using protein extracts from empty vector transformed *E. coli* cells with the respective substrates. Enzyme assays analysis was carried out by LC-MS. MS-MS analysis and putative identification of hydroxylated products are provided in Supplementary Figs. 23-27. Refer Supplementary Table 1 for NMR based assignment of hydroxytomatine compound.

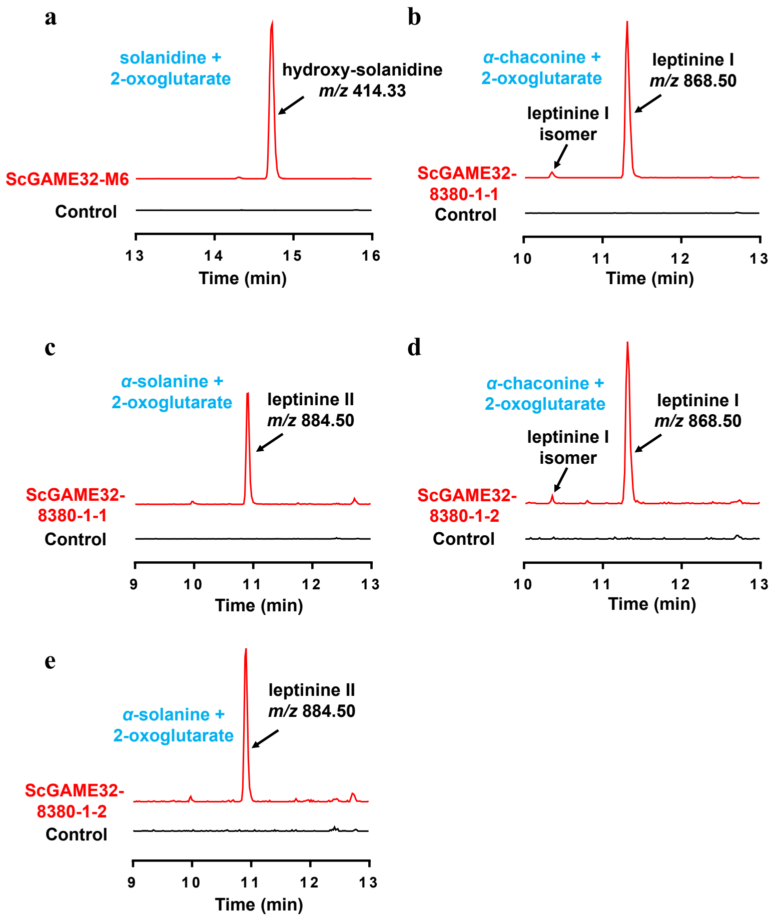

Supplementary Figure 21. Three recombinant GAME32 enzymes from *S. chacoense* act explicitly on core potato steroidal alkaloids to form CPB resistance associated leptinines metabolites. Three recombinant GAME32 enzymes, namely, ScGAME32-M6 (from *S. chacoense* accession M6), ScGAME32-8380-1-1 and ScGAME32-8380-1-2 (*S. chacoense* accession 8380-1) were produced in *E. coli* cells and assayed for their hydroxylase activity. **(a)** Overlay of extracted ion chromatograms of hydroxy-solanidine (*m/z* 414.33, ScGAME32-M6 enzyme assay product, marked in red) and control assay (shown in black) obtained with solanidine as a substrate. **(b, c)** Recombinant ScGAME32-8380-1-1 enzyme assay (in red) with *α*-chaconine and *α*-solanine substrates respectively. **(d, e)** Recombinant ScGAME32-8380-1-2 enzyme assay (in red) with *α*-chaconine and *α*-solanine substrates respectively. For assays shown in panels (b-e), control reactions (in black) with respective substrates (names as above) were carried out with protein extracts derived from empty vector-transformed *E. coli* cells. Mass to charge (*m/z*) is shown for assay products. LC-MS was used for analyzing the products of enzyme assay. MS-MS analysis and identification of hydroxylated compounds (enzyme assay products) are provided in Supplementary Figs. 28-32.

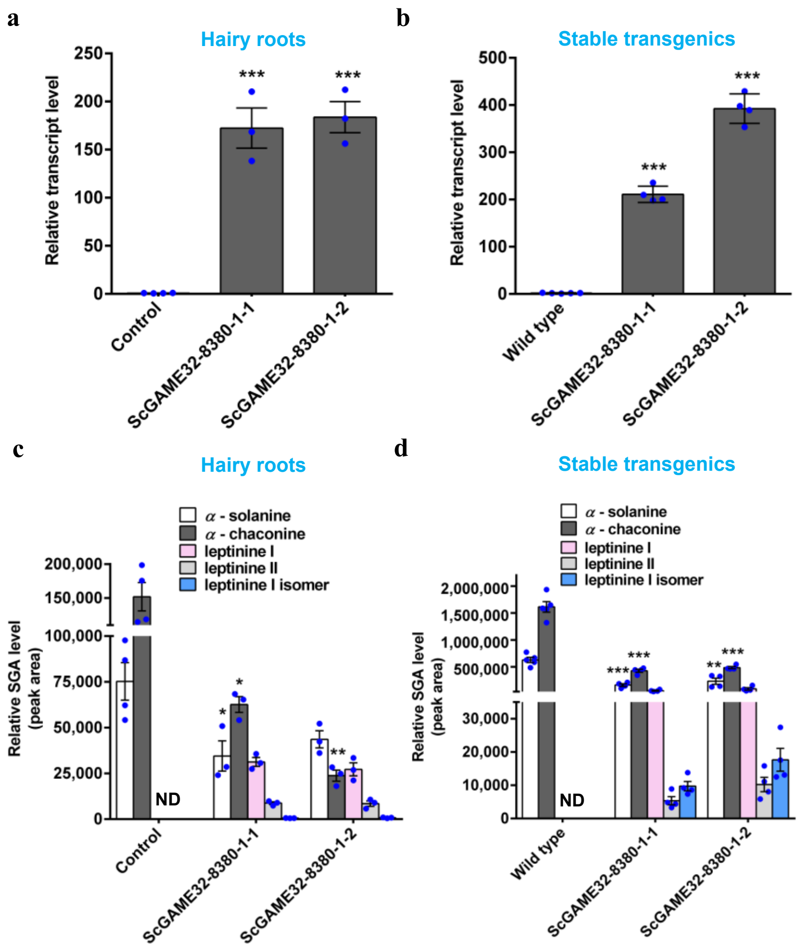

Supplementary Figure 22. Analysis of hairy roots and leaves of stably transformed cultivated potato plants overexpressing wild potato *GAME32* genes. Wild potato *S. chacoense* (*Sc*) genes, *ScGAME32-8380-1-1* and *ScGAME32-8380-1-2* were overexpressed in cultivated potato to generate hairy roots and stable transgenic lines. **(a)** Relative expression level of *ScGAME32-8380-1-1* and *ScGAME32-8380-1-2* genes in potato hairy roots compared to control hairy roots (generated with an empty vector). **(b)** Expression of *ScGAME32-8380-1-1* and *ScGAME32-8380-1-2* in leaves of stable transgenic potato plants compared to wild type (non-transformed) leaves. Gene expression level (panel a and b) was determined by a quantitative Real-Time PCR (qRT-PCR) assay. Values (panel a and b) represent mean ± standard error mean (n ≥ 3 for each genotype). **(c)** Levels of leptinines, *α*-chaconine and *α*-solanine in *ScGAME32-8380-1-1* or *ScGAME32-8380-1-2* overexpressing hairy roots compared to control roots. Values indicate means of biological replicates ± standard error mean (n ≥ 3 for each genotype) either from control or transgenic hairy root line. **(d)** Levels of leptinines and precursor SGAs (i.e. *α*-chaconine and *α*-solanine) in leaves of the *ScGAME32-8380-1-1* or *ScGAME32-8380-1-*2 overexpressing transgenic potato plants compared to wild type leaves. Leptinines were not detected in control (panel c) or wild type (panel d) samples. Values indicate means of biological replicates ± standard error mean from wild type or transgenic line (n ≥ 3 for each genotype). Asterisks (panel a-d) indicate significant changes from control/wild type samples according to experimental genotypes as calculated by a Student's t-test (*P-value < 0.05; **P-value < 0.01; ***P-value < 0.001). Metabolites analysis was done using LC-MS. ND: Not Detected. Source data are provided as a Source Data file.

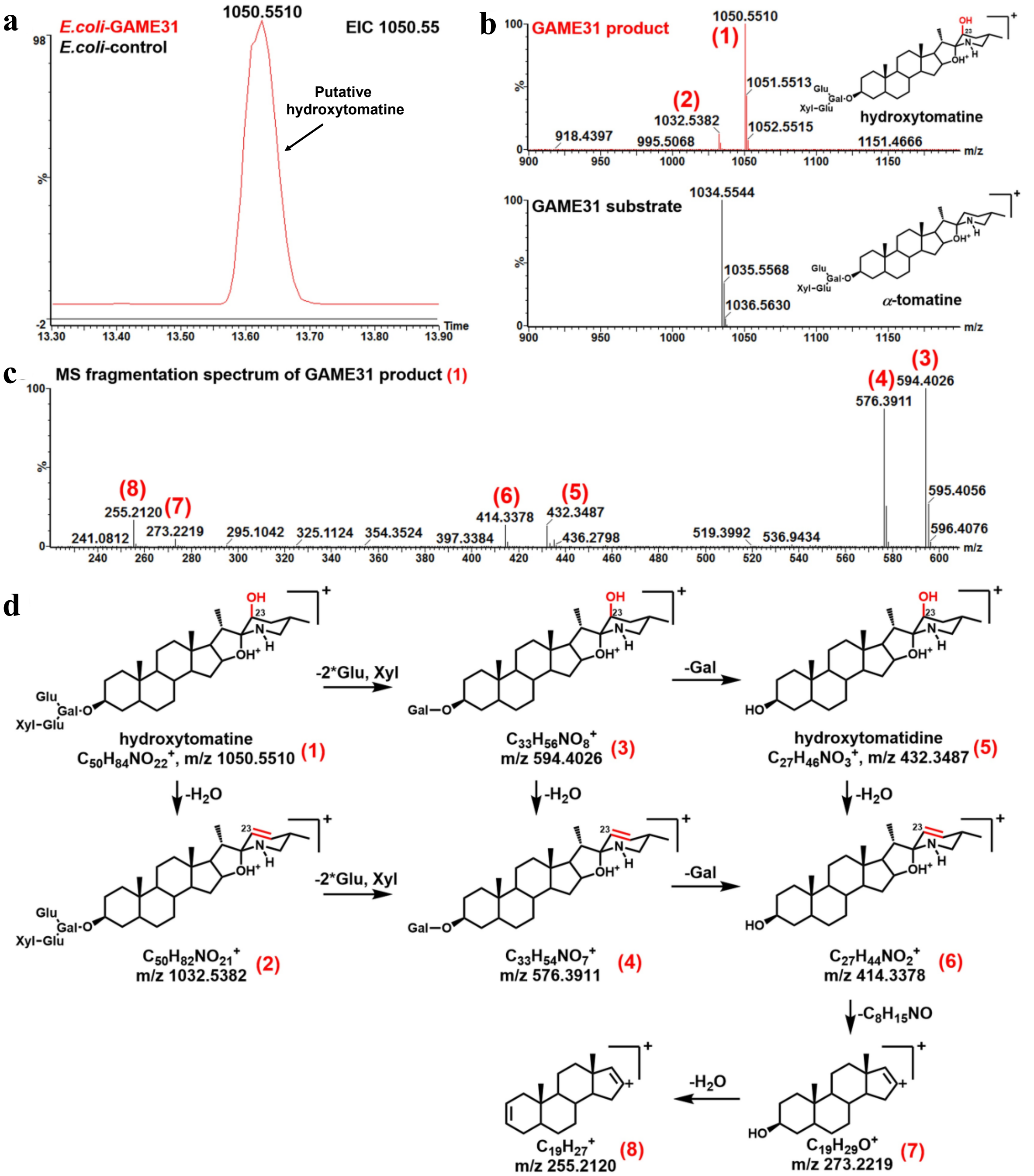

Supplementary Figure 23. LC-MS characterization of recombinant GAME31 enzyme activity assay. GAME31 (either from tomato or eggplant or *S. chacoense*-M6 or *S. chacoense*-8380-1) was expressed in *E. coli* cells. Here, representative GAME31 (from *S. chacoense*-M6) enzyme assay is presented. **(a)** Overlay of extracted ion chromatograms of *m/z* 1050.55 Da $[M+H]^+$, mass of product of GAME31 enzyme reaction and control reaction (using extracts from empty vector transformed *E. coli* cells) using *α*-tomatine as a substrate. **(b)** Mass spectra and structures of detected product (upper panel) and substrate (lower panel) of GAME31 enzymatic reaction with *α*-tomatine as substrate. Hydroxyl-group added by GAME31 is located at C-23 (on F-ring), as confirmed by NMR of isolated hydroxytomatine compound from leaves of *GAME31*-Ox lines in this study (Supplementary Table 1). **(c)** MS-MS fragmentation spectrum of *m/z* 1050.55 Da $[M+H]^+$, a putative hydroxytomatine, fragments are marked with numbers in accordance to panel D. **(d)**

Fragmentation analysis of hydroxytomatine (**1**) (positive ion mode): the enzyme reaction product can undergo two fragmentation pathways. First, either the sugar moieties on C-3 are lost successively forming hydroxy-tomatidine *m/z* 432.34 Da (**5**), via the intermediated *m/z* 594.40 Da $[M+H-2*Glu-Xyl]^+$ (**3**). Further hydroxy-tomatidine *m/z* 432.34 Da (**5**) is converted to *m/z* 414.33 Da (**6**) by loss of a water molecule. Alternatively *m/z* 1050.55 Da (**1**) loses a molecule of water to form *m/z* 1032.53 Da (**2**) which loses the sugar residues at C-3 successively to form *m/z* 414.33 Da (**6**), via the intermediate *m/z* 576.39 Da $[M+H-H_2O-2*Glu-Xyl]^+$ (**4**). Ion *m/z* 414.33 Da (**6**) undergoes further fragmentation to form the characteristic aglycone fragmentation products for *α*-tomatine/tomatidine *m/z* 273.22 Da (**7**) and 255.21 Da (**8**). Detection of these fragments also confirms the localization of the newly incorporated hydroxyl-group at the E/F ring part of the molecule and not on the rings A-D of the cholesterol backbone[38]. *m/z*: mass to charge; EIC: extracted ion chromatogram; Xyl: xylose; Glu: glucose; Gal: galactose.

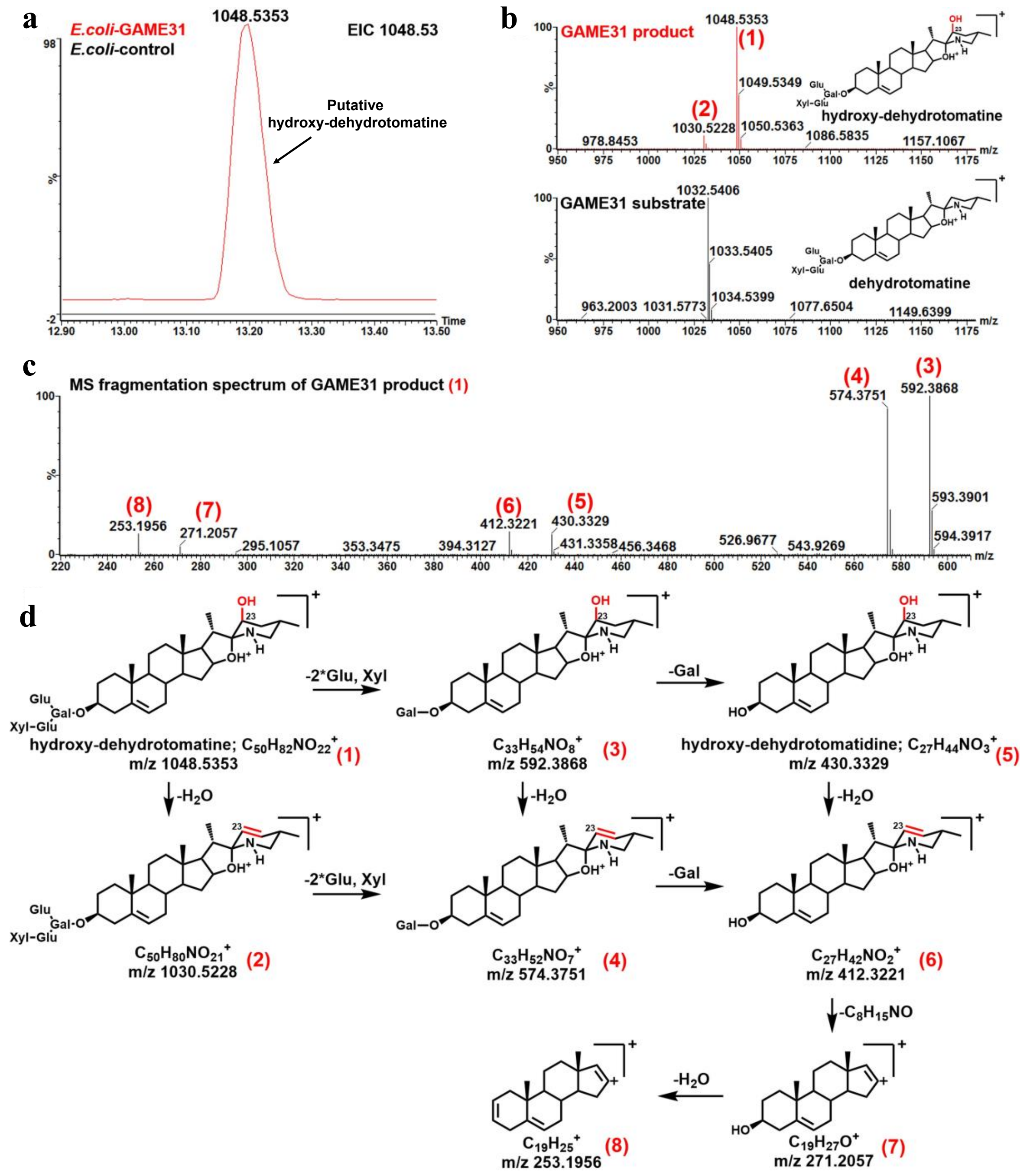

Supplementary Figure 24. Characterization of GAME31 by *in vitro* enzyme assay. GAME31 (either from tomato or eggplant or *S. chacoense*-M6 or *S. chacoense*-8380-1) was expressed in *E. coli* cells. Here, representative GAME31 (from *S. chacoense*-M6) enzyme assay is presented. **(a)** Overlay of extracted ion chromatograms of *m/z* 1048.53 Da, $[M+H]^+$, mass of product of GAME31 enzyme reaction and control reaction (performed using extracts from *E. coli* cells transformed with empty vector) using dehydrotomatine as a substrate. **(b)** Mass spectra and structures of detected product (upper panel) and substrate (lower panel) of GAME31 enzymatic reaction with dehydrotomatine as substrate. **(c)** MS-MS fragmentation spectrum of *m/z* 1048.53 Da $[M+H]^+$, a putative hydroxy-dehydrotomatine, fragments are marked with numbers in accordance to panel D. **(d)** Fragmentation analysis of hydroxy-dehydrotomatine (**1**) (positive ion mode): the enzyme reaction product can undergo two fragmentation pathways. First, either the sugar moieties on C-3 are lost

successively forming hydroxy-dehydrotomatidine *m/z* 430.33 Da (**5**), via the intermediated *m/z* 592.38 Da $[M+H-2*Glu-Xyl]^+$ (**3**). Further (**5**) is converted to *m/z* 412.33 Da (**6**) by a loss of a water molecule. Alternatively (**1**) loses a molecule of water to form *m/z* 1030.52 Da (**2**) which loses the sugar residues at C-3 successively to form (**6**), via the intermediate *m/z* 574.37 Da $[M+H-H_2O-2*Glu-Xyl]^+$ (**4**). Ion *m/z* 412.33 Da (**6**) undergoes further fragmentation to form the characteristic aglycone fragmentation products for dehydrotomatine/dehydrotomatidine *m/z* 271.20 Da (**7**) and 251.19 Da (**8**). Detection of these fragments also confirms the localization of the newly incorporated hydroxyl-group at the E/F ring part of the molecule and not on the rings A-D of the cholesterol backbone[38]. Based on NMR assignment of hydroxytomatine in this study (Supplementary Table 1), we suggested the C-23 (on F-ring) position for hydroxyl group in hydroxy-dehydrotomatine. Abbreviations: *m/z*: mass to charge; EIC: extracted ion chromatogram; Xyl: xylose; Glu: glucose; Gal: galactose.

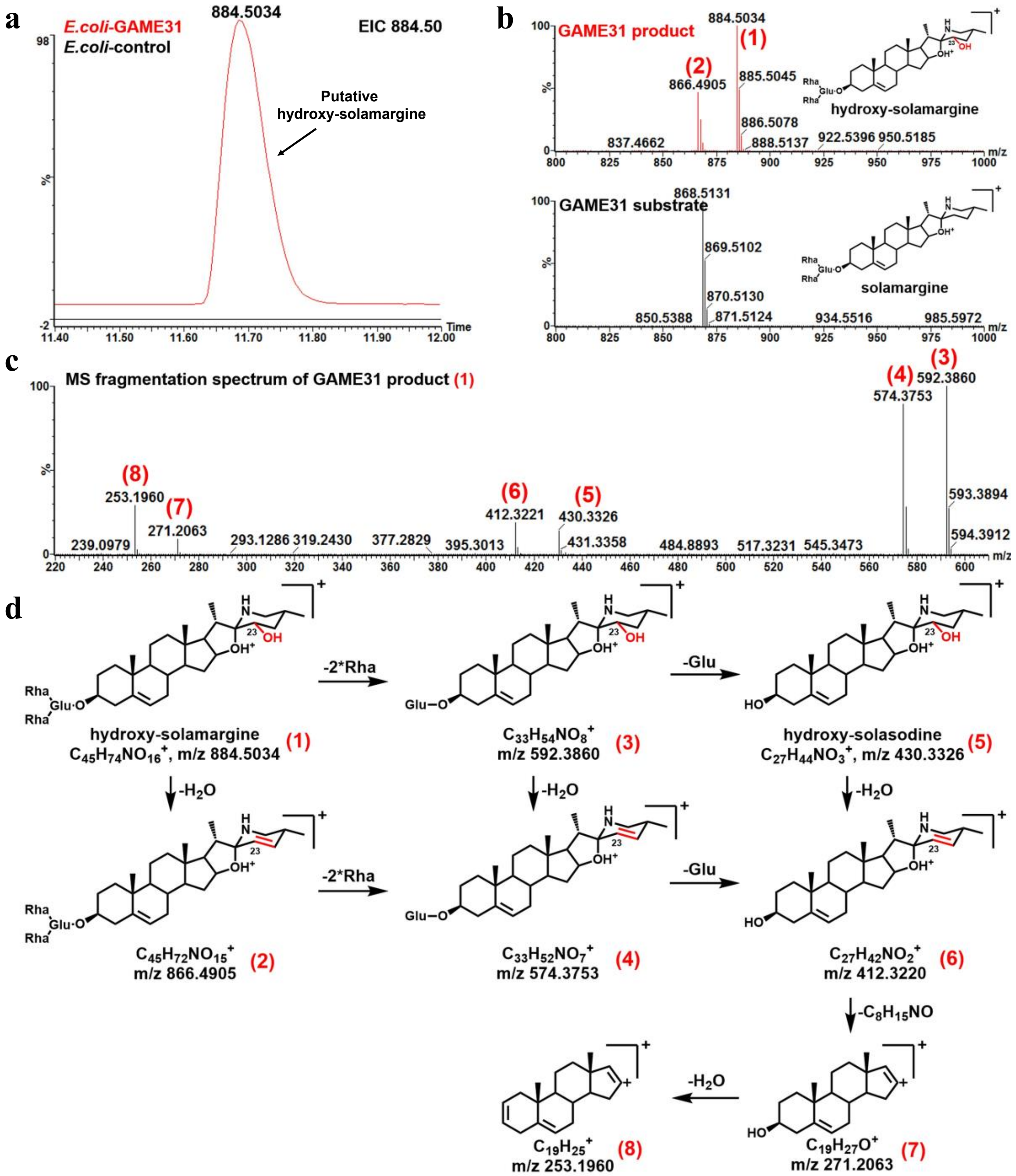

Supplementary Figure 25. Characterization of GAME31 by *in vitro* enzyme activity assay. GAME31 (either from tomato or eggplant or *S. chacoense*-M6 or *S. chacoense*-8380-1) was expressed in *E. coli* cells. Here, representative GAME31 (from *S. chacoense*-M6) enzyme assay is presented. **(a)** Overlay of extracted ion chromatograms of *m/z* 884.50 Da $[M+H]^+$, mass of product of GAME31 enzyme reaction and control reaction (carried out using extracts from *E. coli* transformed with empty vector) using α-solamargine as a substrate. **(b)** Mass spectra and structures of detected product (upper panel) and substrate (lower panel) of GAME31 enzymatic reaction with α-solamargine as substrate. **(c)** MS-MS fragmentation spectrum of *m/z*

884.50 Da $[M+H]^+$, a putative hydroxy-solamargine, fragments are marked with numbers in accordance to panel D. **(d)** Mass fragmentation analysis of hydroxy-solamargine (**1**) (positive ion mode): the enzyme reaction product can undergo two fragmentation pathways. In the first route, either the sugar moieties on C-3 are lost successively forming hydroxy-solasodine *m/z* 430.33 Da (**5**), via the intermediated *m/z* 592.38 Da $[M+H-2*Rha]^+$ (**3**). Hydroxy-solasodine (**5**) is further converted to *m/z* 412.32 Da (**6**) by loss of a water molecule. Alternatively (**1**) loses a molecule of water to form *m/z* 866.49 Da (**2**) which loses the sugar residues at C-3 successively to form (**6**), via the intermediate *m/z* 574.37 Da $[M+H-H_2O-2*Rha]^+$ (**4**). Ion *m/z* 412.32 Da (**6**) undergoes further fragmentation to form the characteristic aglycone fragmentation products for *α*-solamargine/solasodine *m/z* 271.20 Da (**7**) and 253.19 Da (**8**). Detection of these fragments also confirms the localization of the newly incorporated hydroxyl-group at the E/F ring part of the molecule and not on the rings A-D of the cholesterol backbone[38]. Based on NMR assignment of hydroxytomatine in this study (Supplementary Table 1), we suggested the C-23 (on F-ring) position for hydroxyl group in hydroxy-solamargine. LC-MS was used for analysis of enzyme assay products. Abbreviations: *m/z*: mass to charge; EIC: extracted ion chromatogram; Rha: rhamnose; Glu: glucose.

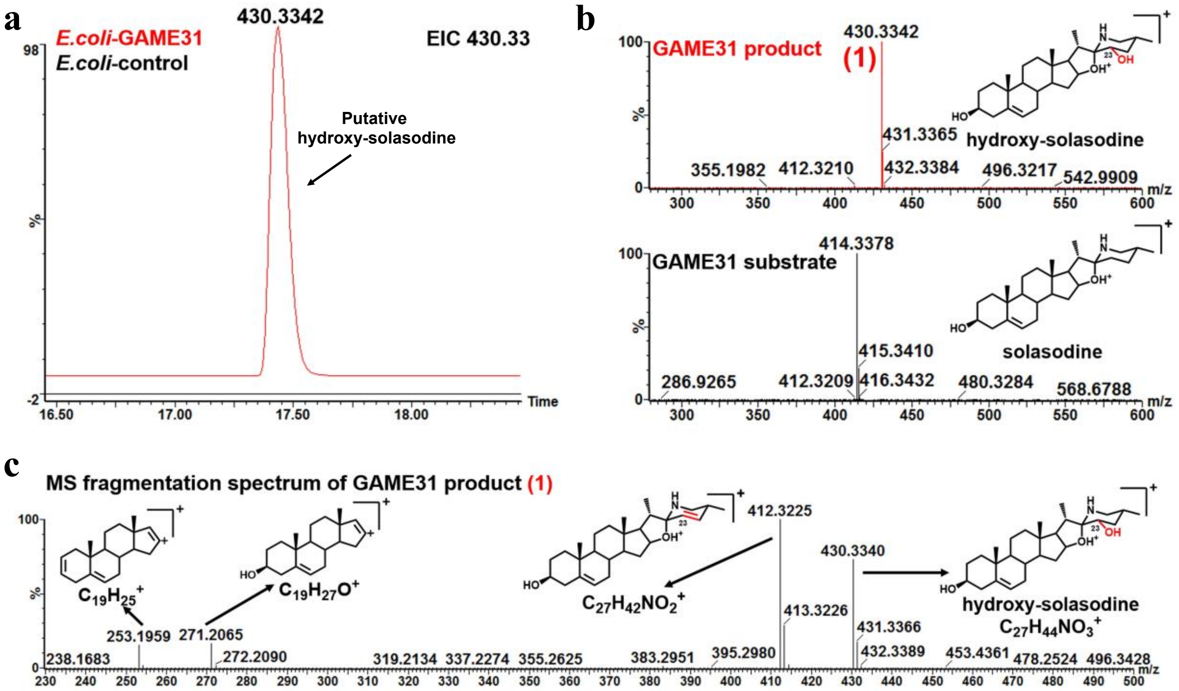

Supplementary Figure 26. Characterization of GAME31 by *in vitro* enzyme activity assay. GAME31 (either from tomato or eggplant or *S. chacoense*-M6 or *S. chacoense*-8380-1) was expressed in *E. coli* cells. Here, representative GAME31 (from *S. chacoense*-M6) enzyme assay is presented. **(a)** Overlay of extracted ion chromatograms of *m/z* 430.33 Da $[M+H]^+$, mass of product of GAME31 enzyme reaction and control reaction (empty vector transformed *E. coli* cell extracts) using solasodine as a substrate. **(b)** Mass spectra and structures of detected product (upper panel) and substrate (lower panel) of GAME31 enzymatic reaction with solasodine as substrate. **(c)** MS-MS fragmentation spectrum of *m/z* 430.33, a putative hydroxy-solasodine. Hydroxy-solasodine first loses a water molecule resulting in ion *m/z* 412.3215, which further fragments into the characteristic aglycone fragments of saturated glycoalkaloids *m/z* 271.2065 and 253.1959. Structures and molecular formulas of the fragment ions are shown on the spectrum. Based on NMR assignment of hydroxytomatine in this study (Supplementary Table 1), we suggested the C-23 (on F-ring) position for hydroxyl group in hydroxy-solasodine. Abbreviations: *m/z*: mass to charge; EIC: extracted ion chromatogram.

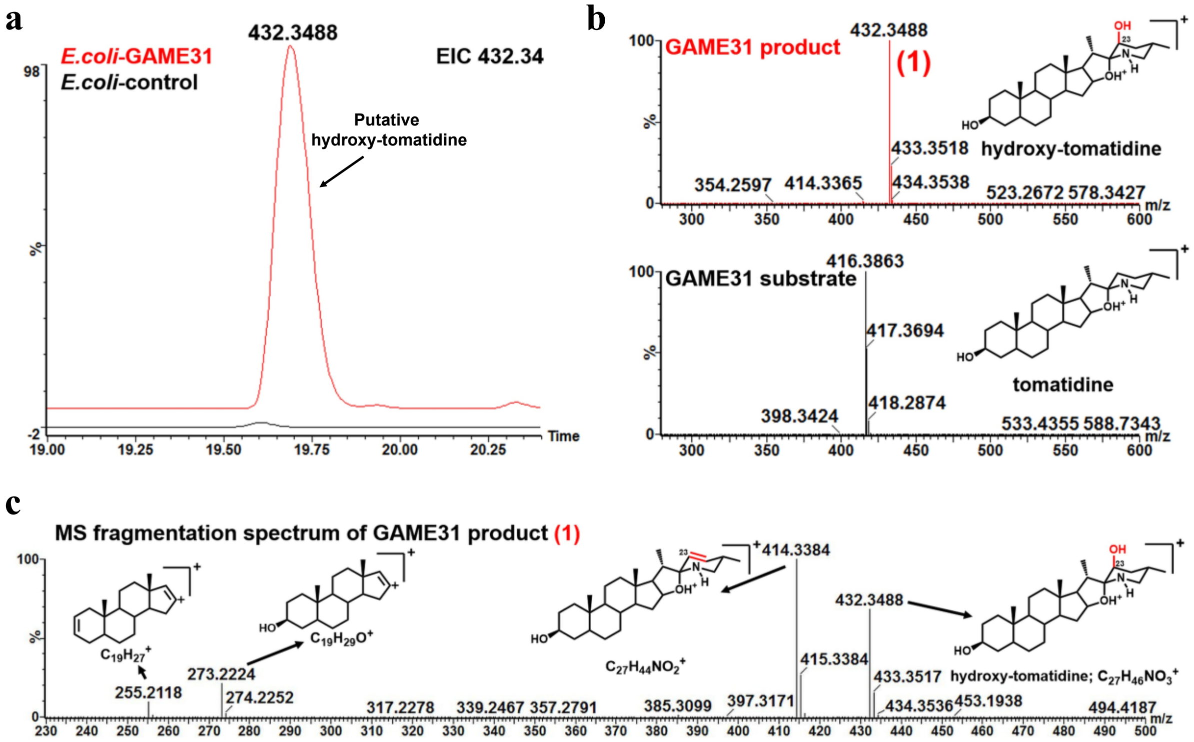

Supplementary Figure 27. Characterization of GAME31 by *in vitro* enzyme assay. The *GAME31* gene was isolated from the M6 and 8380-1 accessions of *S. chacoense* (*Sc*) and expressed in *E. coli* cells. Here, representative GAME31 (from *S. chacoense*-M6) enzyme assay is presented. **(a)** Overlay of extracted ion chromatograms of *m/z* 432.34 Da $[M+H]^+$, mass of product of GAME31 reaction and control reaction (using extracts from *E. coli* cells transformed with empty vector) using tomatidine as a substrate. **(b)** Mass spectra and structures of detected product (upper panel) and substrate (lower panel) of GAME31 enzymatic reaction with tomatidine as substrate. **(c)** MS-MS fragmentation spectrum of *m/z* 432.34, a putative hydroxy-tomatidine. Hydroxy-tomatidine first loses a water molecule resulting in ion *m/z* 414.3384, which further fragments into the characteristic aglycone fragments of saturated glycoalkaloids *m/z* 273.2224 and 255.2118. Structures and molecular formulas of the fragment ions are shown on the spectrum. Hydroxyl-group added by GAME31 is located at C-23 (on F-ring), as confirmed by NMR of isolated hydroxytomatine compound from leaves of *GAME31*-Ox lines in this study (Supplementary Table 1). Enzyme assay products were analyzed by LC-MS. Abbreviations: *m/z*: mass to charge; EIC: extracted ion chromatogram.

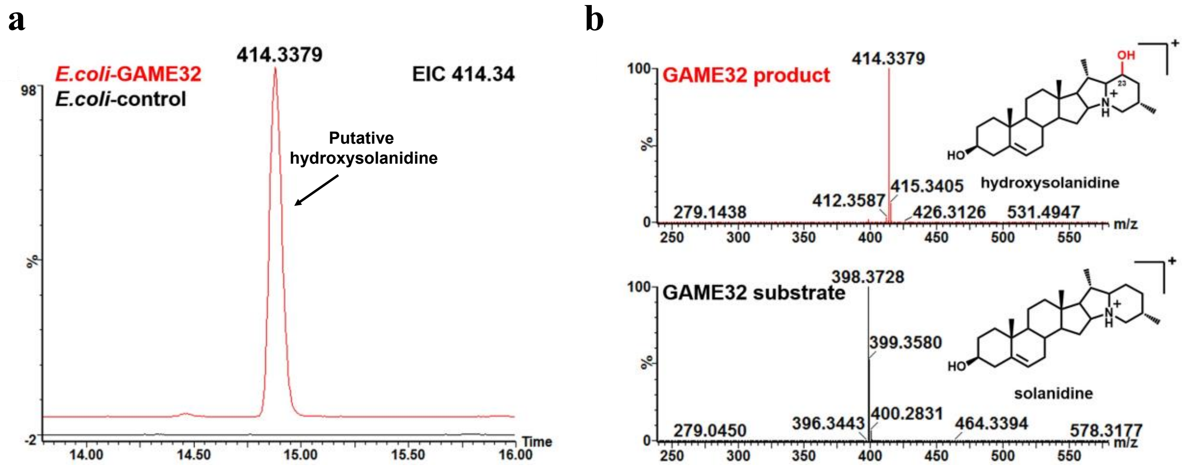

Supplementary Figure 28. LC-MS analysis of the recombinant GAME32 enzyme assay with solanidine. GAME32 (either from *S. chacoense*-M6 or *S. chacoense*-8380-1) was expressed in *E. coli* cells. Here, representative GAME32 (from *S. chacoense*-M6) enzyme assay is presented. **(a)** Overlay of extracted ion chromatograms of *m/z* 414.34 Da, [M+H^{+}], mass of product of GAME32 enzyme reaction and control reaction using solanidine as a substrate. **(b)** Mass spectra and structures of detected product, a putative hydroxy-solanidine (upper panel) and substrate (lower panel) of GAME32 enzymatic reaction with solanidine as substrate. Abbreviations: *m/z*: mass to charge; EIC: extracted ion chromatogram.

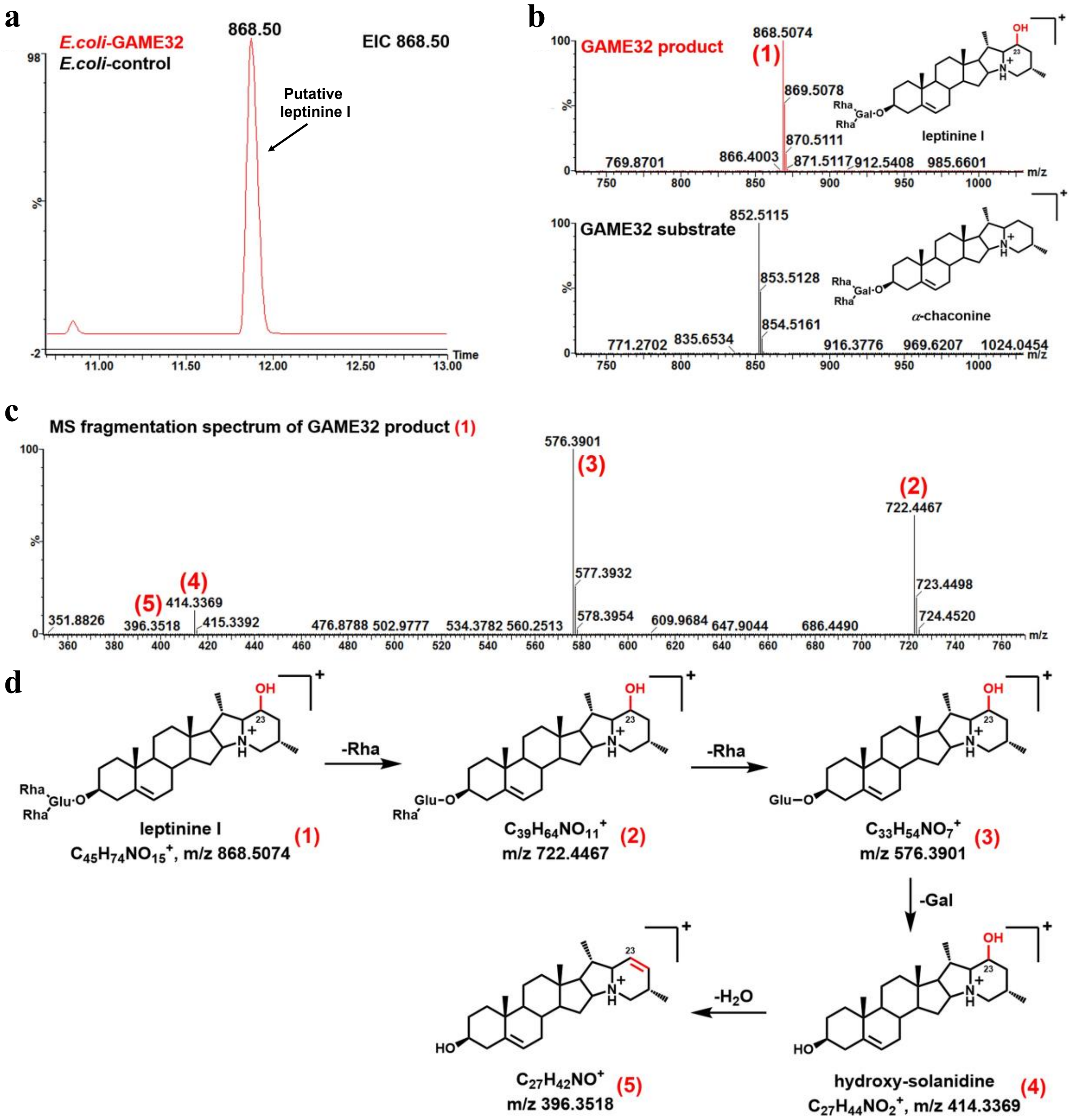

Supplementary Figure 29. Characterization of GAME32 by *in vitro* enzyme activity assay. GAME32 (either from *S. chacoense*-M6 or *S. chacoense*-8380-1) was expressed in *E. coli* cells. Here, representative GAME32 (from *S. chacoense*-M6) enzyme assay is presented. **(a)** Overlay of extracted ion chromatograms of *m/z* 868.50 Da $[M+H]^+$, mass of product of GAME32 of enzyme reaction and control reaction using *α*-chaconine as a substrate. **(b)** Mass spectra and structures of detected product (upper panel) and substrate (lower panel) of GAME32 enzymatic reaction with *α*-chaconine as substrate. **(c)** MS-MS fragmentation spectrum of *m/z* 868.50 $[M+H]^+$, a putative leptinine I, fragments are marked with numbers in

accordance to panel D. **(d)** fragmentation analysis of leptinine I (**1**) (positive ion mode): the enzyme reaction product loses first the sugars attached to the C-3 hydroxyl-group, 2*Rha [(*m/z* 722.44 Da, (**2**)), (*m/z* 576.39 Da, (**3**))] and Gal leading to the hydroxy-solanidine ion, *m/z* 412.33 Da $[M+H]^+$ (**4**), which undergoes dehydration resulting in the formation of *m/z* 396.35 (**5**). Control reaction was carried out with the respective substrate and the protein extracts of empty vector-transformed *E. coli*. Abbreviations: *m/z*: mass to charge; EIC: extracted ion chromatogram; Rha: rhamnose; Gal: galactose. Refer Supplementary Fig. 31 for high energy (120 eV) MS-MS analysis for putative identification of leptinine I. Based on characteristic fragments observed there, we confirmed the localization of the newly incorporated hydroxyl-group at the E/F ring part of the leptinine I and not on the rings A-D of the cholesterol backbone·

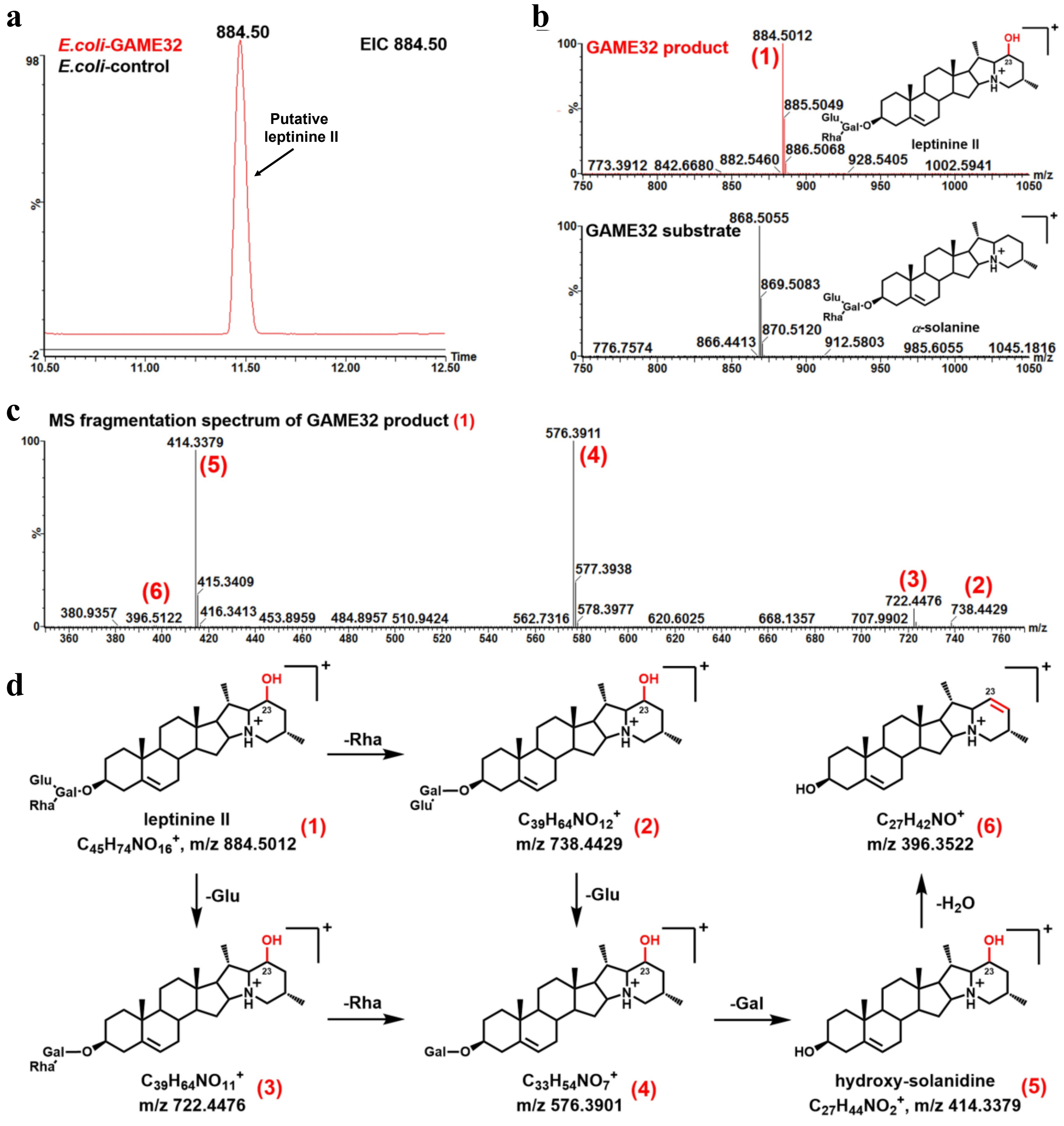

Supplementary Figure 30. Characterization of GAME32 by *in vitro* enzyme activity assay. GAME32 (either from *S. chacoense*-M6 or *S. chacoense*-8380-1) was expressed in *E. coli* cells. Here, representative GAME32 (from *S. chacoense*-M6) enzyme assay is presented. **(a)** Overlay of extracted ion chromatograms of *m/z* 884.50 Da, $[M+H]^+$, mass of product of GAME32 enzyme reaction and control reaction using *α*-solanine as a substrate. **(b)** Mass spectra and structures of detected product (upper panel) and substrate (lower panel) of GAME32 enzymatic reaction with *α*-solanine as substrate. **(c)** MS-MS fragmentation spectrum of *m/z* 884.50 Da $[M+H]^+$, a putative leptinine II, fragments are marked with numbers in accordance to

panel D. **(d)** Fragmentation analysis of leptinine II (**1**) (positive ion mode): the enzyme reaction product loses first the sugars attached to the C-3 hydroxyl-group, either first the rhamnosyl moiety resulting in the ion *m/z* 738.44 Da (**2**) or the glucosyl moiety leading to *m/z* 722.44 Da (**3**). Both (**2**) & (**3**) get deglycosylated resulting in formation of m*/z* 576.39 Da (**4**). Loss of the galactosyl residue of (**4**) leads to the hydroxy-solanidine ion *m/z* 414.33 Da (**5**) which undergoes water-loss to form *m/z* 396.33 Da (**6**). Control reaction was performed using respective substrate and protein extracts from *E. coli* cells transformed with empty pET28 vector. Abbreviations: *m/z*: mass to charge; EIC: extracted ion chromatogram; Rha: rhamnose; Glu: glucose; Gal: galactose. Refer Supplementary Fig. 32 for high energy (120 eV) MS-MS analysis for putative identification of leptinine II. Based on characteristic fragments observed there, we confirmed the localization of the newly incorporated hydroxyl-group at the E/F ring part of the leptinine II and not on the rings A-D of the cholesterol backbone.

a

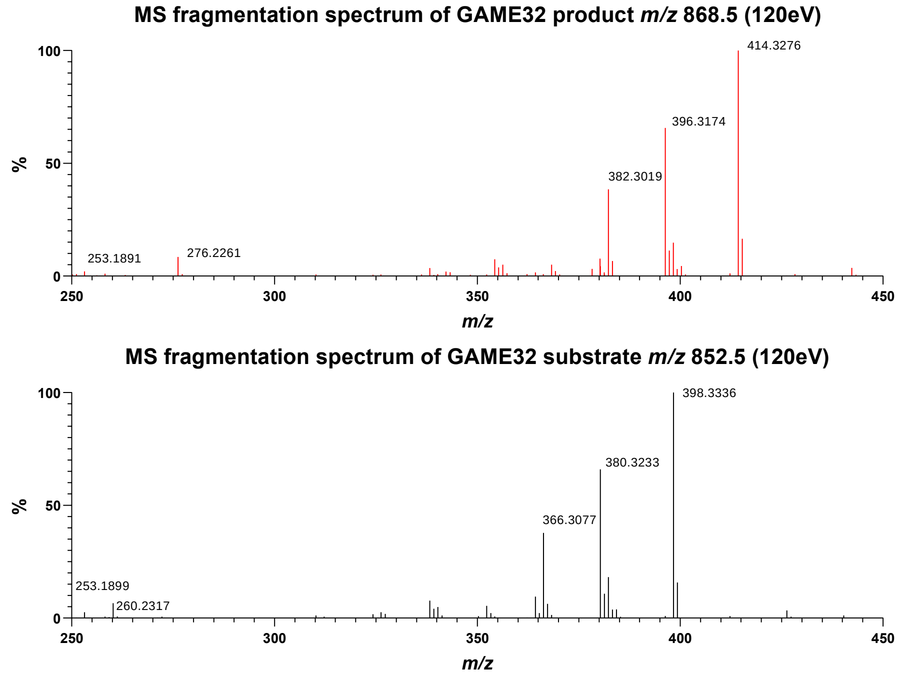

MS fragmentation spectrum of GAME32 product m/z 868.5 (120eV)
100
50
0
%
414.3276
396.3174
382.3019
253.1891
276.2261
250
300
350
400
450
m/z
MS fragmentation spectrum of GAME32 substrate m/z 852.5 (120eV)
100
50
0
%
398.3336
380.3233
366.3077
253.1899
260.2317
250
300
350
400
450
m/z

b

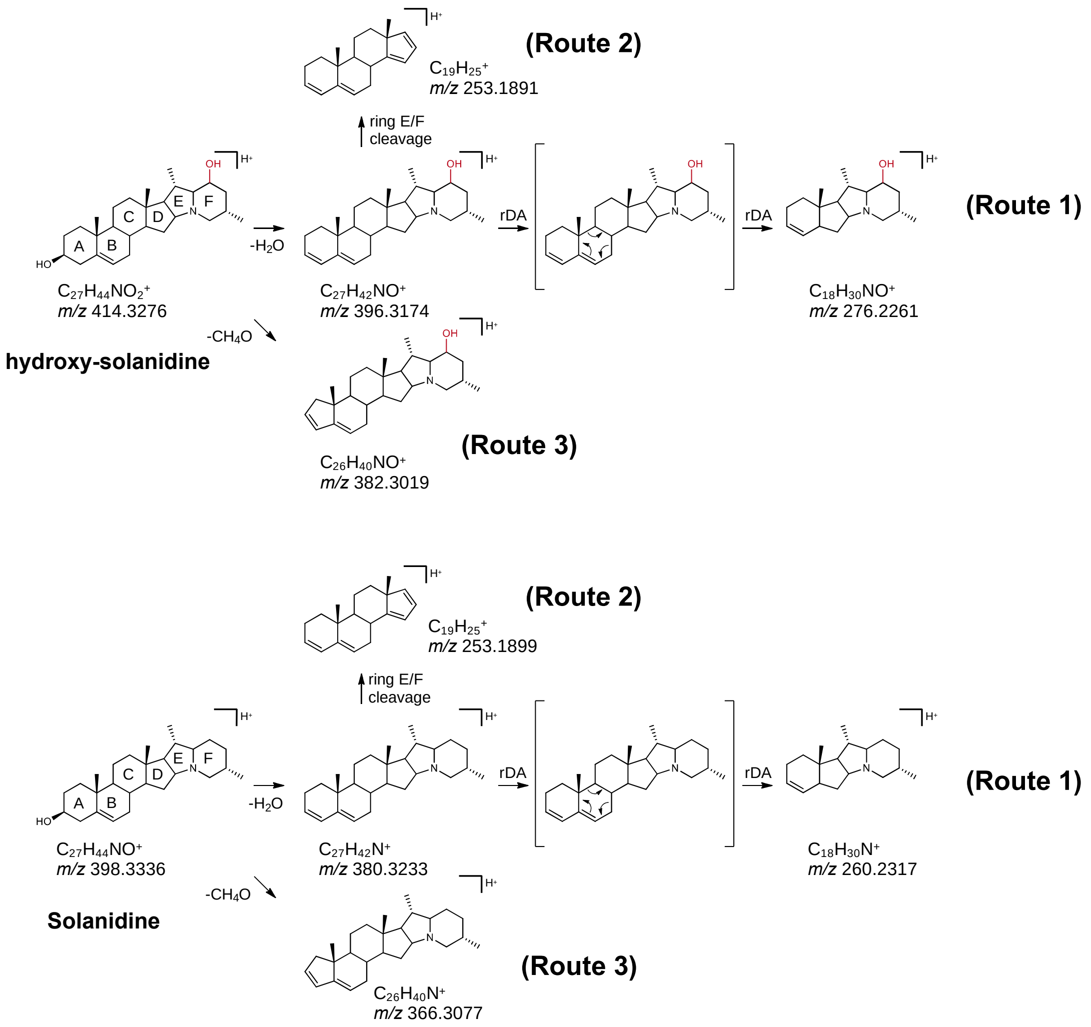

(Route 2)
C19H25+
m/z 253.1891
ring E/F cleavage
HO
A
B
C
D
E
F
N
OH
H+
-H2O
rDA
rDA
(Route 1)
C27H44NO2+
m/z 414.3276
C27H42NO+
m/z 396.3174
C18H30NO+
m/z 276.2261
-CH4O
hydroxy-solanidine
(Route 3)
C26H40NO+
m/z 382.3019
(Route 2)
C19H25+
m/z 253.1899
ring E/F cleavage
-H2O
rDA
rDA
(Route 1)
C27H44NO+
m/z 398.3336
C27H42N+
m/z 380.3233
C18H30N+
m/z 260.2317
-CH4O
Solanidine
(Route 3)
C26H40N+
m/z 366.3077

Supplementary Figure 31. Higher energy MS-MS analysis of the GAME32 enzyme assay product with *α*-chaconine as a substrate. GAME32 (either from *S. chacoense*-M6 or *S. chacoense*-8380-1) was expressed in *E. coli* cells. Here, representative GAME32 (from *S. chacoense*-M6) enzyme assay product analysis is presented. Loss of C-3 sugars in leptinine I and *α*-chaconine resulted in hydroxy-solanidine and solanidine aglycones respectively. **(a)** Comparison between the MS-MS fragmentation spectra (120 ev) of the hydroxy-solanidine (upper panel) and solanidine (lower panel). **(b)** Annotation of ions observed in hydroxy-solanidine (upper panel) and solanidine (lower panel) MS-MS analysis, Upper panel: the fragmentation 'Route 1' is the sequential loss of water molecule (*m/z* 396.31) followed by a retro-Diels–Alder (rDA) reaction and finally loss of A/B ring (*m/z* 276.22); the second fragmentation route ('Route 2') involves loss of the E & F-rings from the molecule *m/z* 396.31 resulting in the major fragment $C_{19}H_{25}^{+}$ (*m/z* 253.18); Route 3 involves loss of CH_4O giving fragment $C_{26}H_{40}NO^{+}$ (*m/z* 382.30). Lower panel: the fragmentation 'Route 1' is the sequential loss of water molecule (*m/z* 380.32) followed by a retro-Diels–Alder (rDA) reaction and finally loss of A/B ring (*m/z* 260.23); the second fragmentation route ('Route 2') involves loss of the E & F-rings from the molecule *m/z* 380.32 resulting in the same major fragment $C_{19}H_{25}^{+}$ (*m/z* 253.18); Route 3 involves loss of CH_4O giving fragment $C_{26}H_{40}NO^{+}$ (*m/z* 366.30). Based on characteristic fragments observed here, we confirmed the localization of the newly incorporated hydroxyl-group at the E/F-ring part of the leptinine I, which is consistent with proposed leptinine I structure and not on the rings A-D of the cholesterol backbone.

a

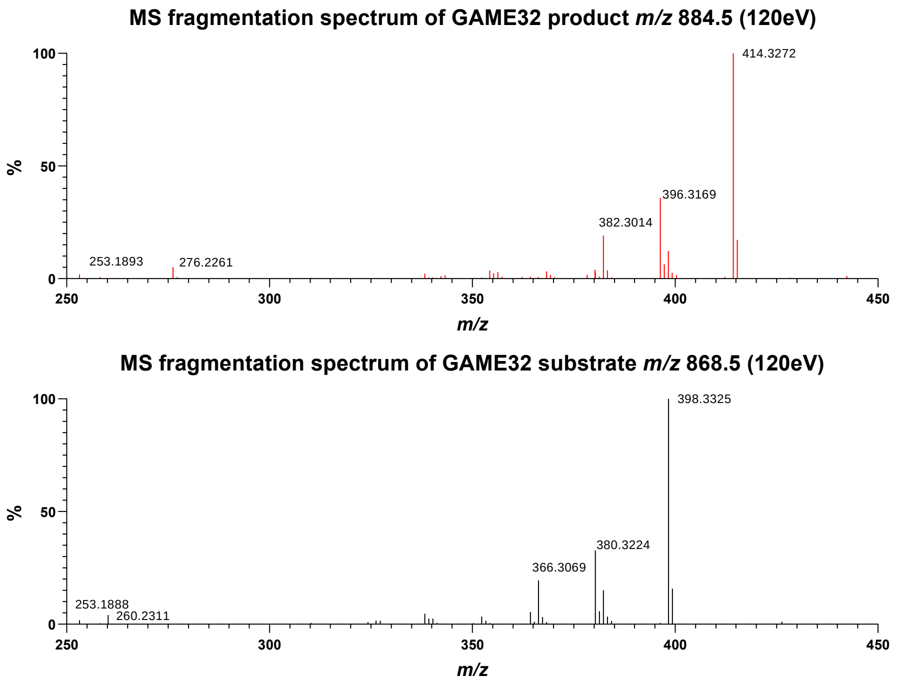
MS fragmentation spectrum of GAME32 product m/z 884.5 (120eV)
100
50
0
%
414.3272
396.3169
382.3014
253.1893
276.2261
250
300
350
400
450
m/z
MS fragmentation spectrum of GAME32 substrate m/z 868.5 (120eV)
100
50
0
%
398.3325
380.3224
366.3069
253.1888
260.2311
250
300
350
400
450
m/z

b

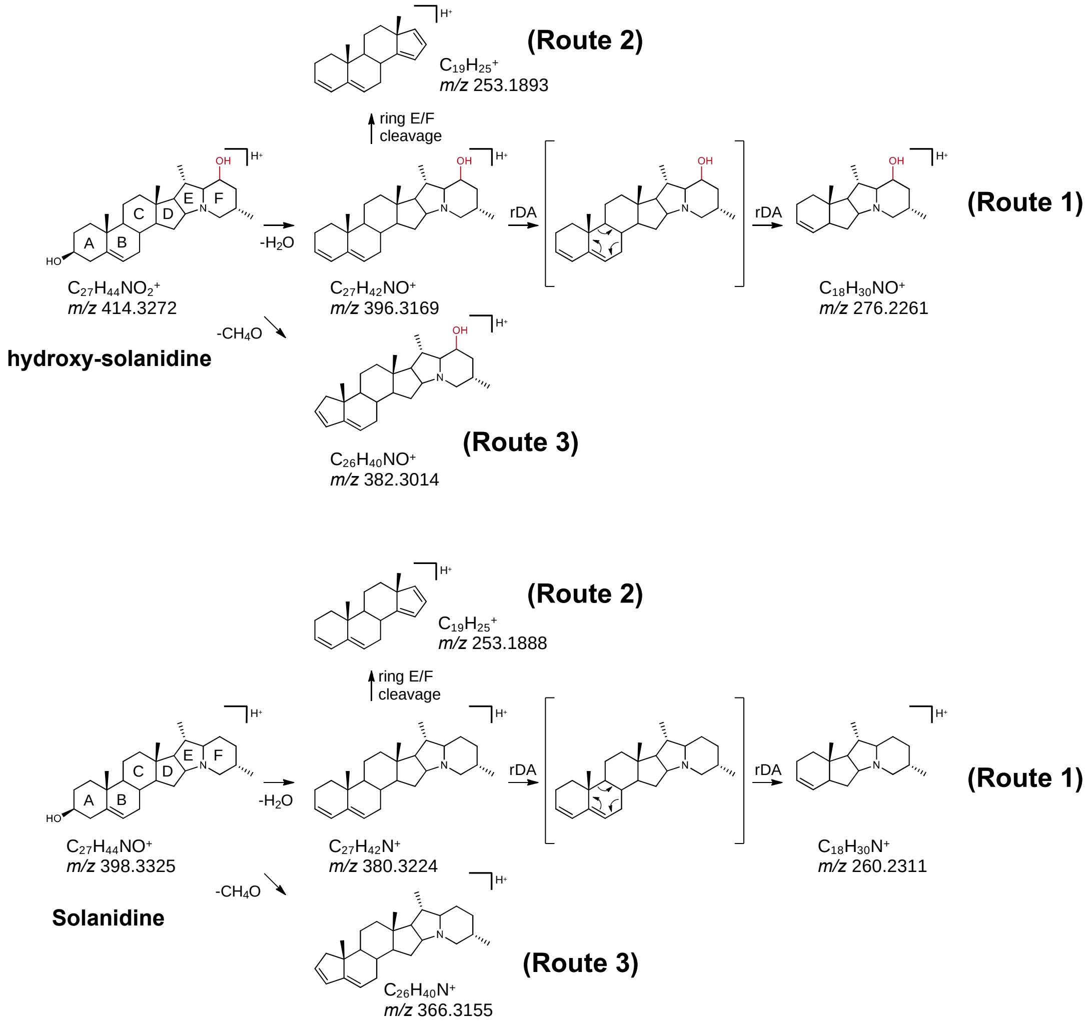
(Route 2)
C19H25+
m/z 253.1893
ring E/F cleavage
OH
H+
A
B
C
D
E
F
N
HO
-H2O
rDA
rDA
(Route 1)
C27H44NO2+
m/z 414.3272
C27H42NO+
m/z 396.3169
C18H30NO+
m/z 276.2261
-CH4O
hydroxy-solanidine
(Route 3)
C26H40NO+
m/z 382.3014
(Route 2)
C19H25+
m/z 253.1888
ring E/F cleavage
C27H44NO+
m/z 398.3325
C27H42N+
m/z 380.3224
C18H30N+
m/z 260.2311
-CH4O
Solanidine
(Route 3)
C26H40N+
m/z 366.3155

Supplementary Figure 32. MS-MS analysis of the GAME32 enzyme assay product with α-solanine as a substrate. GAME32 (either from *S. chacoense*-M6 or *S. chacoense*-8380-1) was expressed in *E. coli* cells. Here, representative GAME32 (from *S. chacoense*-M6) enzyme assay product analysis is presented. Loss of C-3 sugars in leptinine II and *α*-solanine resulted in hydroxy-solanidine and solanidine aglycones respectively. **(a)** Comparison between the MS-MS fragmentation spectra (120 ev) of the hydroxy-solanidine (upper panel) and solanidine (lower panel). **(b)** Annotation of ions observed in hydroxy-solanidine (upper panel) and solanidine (lower panel) MS-MS analysis, Upper panel: the fragmentation 'Route 1' is the sequential loss of water molecule (*m/z* 396.31) followed by a retro-Diels–Alder (rDA) reaction and finally loss of A/B ring (*m/z* 276.22); the second fragmentation route ('Route 2') involves loss of the E & F-rings from the molecule *m/z* 396.31 resulting in the major fragment $C_{19}H_{25}^{+}$ (*m/z* 253.18); Route 3 involves loss of CH_4O giving fragment $C_{26}H_{40}NO^{+}$ (*m/z* 382.30). Lower panel: the fragmentation 'Route 1' is the sequential loss of water molecule (*m/z* 380.32) followed by a retro-Diels–Alder (rDA) reaction and finally loss of A/B ring (*m/z* 260.23); the second fragmentation route ('Route 2') involves loss of the E & F-rings from the molecule *m/z* 380.32 resulting in the same major fragment $C_{19}H_{25}^{+}$ (*m/z* 253.18); Route 3 involves loss of CH_4O giving fragment $C_{26}H_{40}NO^{+}$ (*m/z* 366.30). Based on characteristic fragments observed here, we confirmed the localization of the newly incorporated hydroxyl-group at the E/F-ring part of the leptinine II, which is consistent with proposed leptinine II structure and not on the rings A-D of the cholesterol backbone.

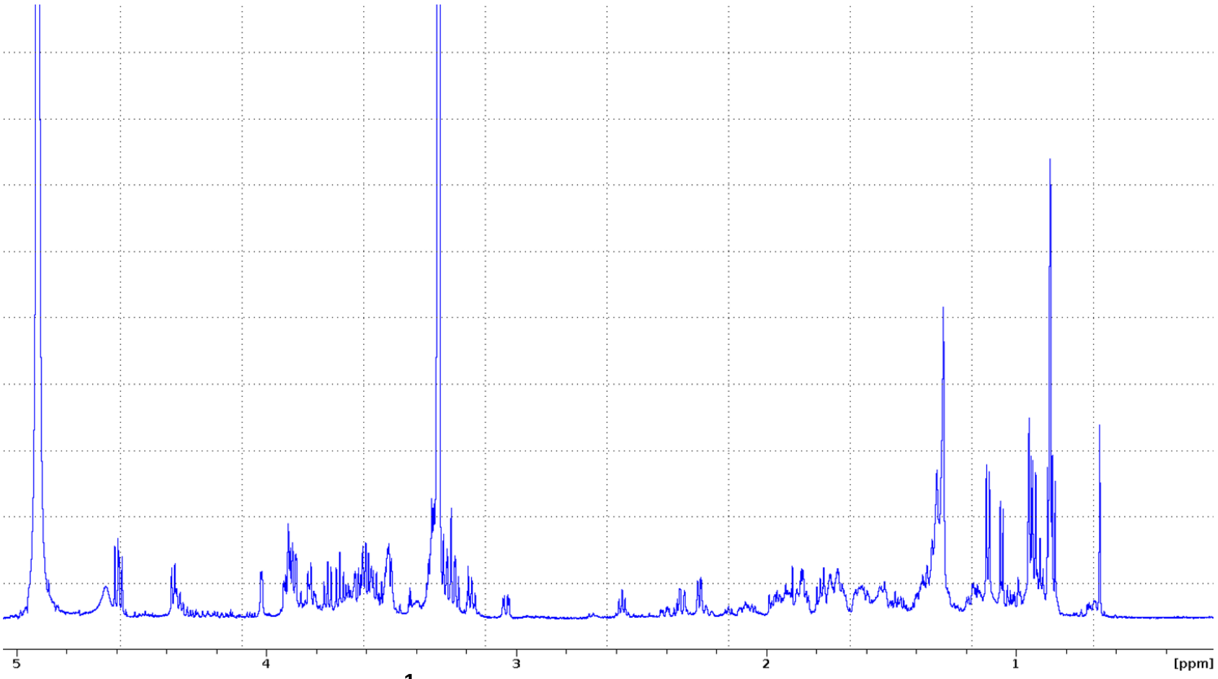

Supplementary Figure 33. ^{1}H NMR Spectrum of hydroxytomatine.

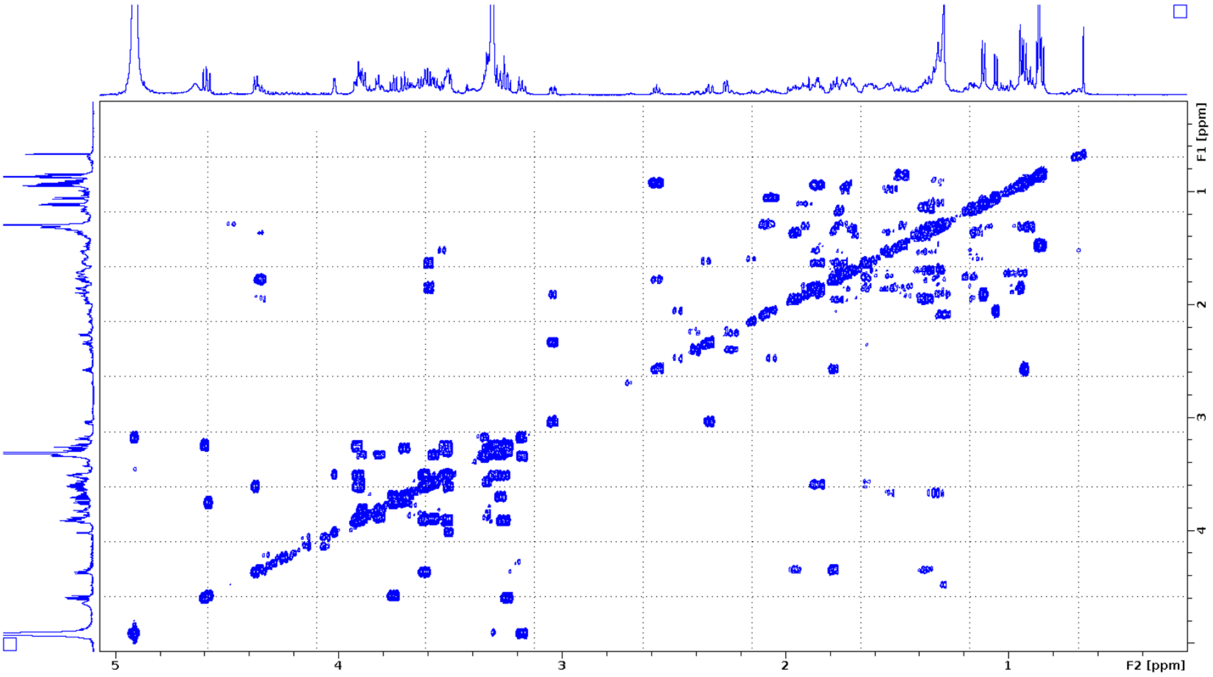

Supplementary Figure 34. 2D dqf-COSY NMR Spectrum of hydroxytomatine.

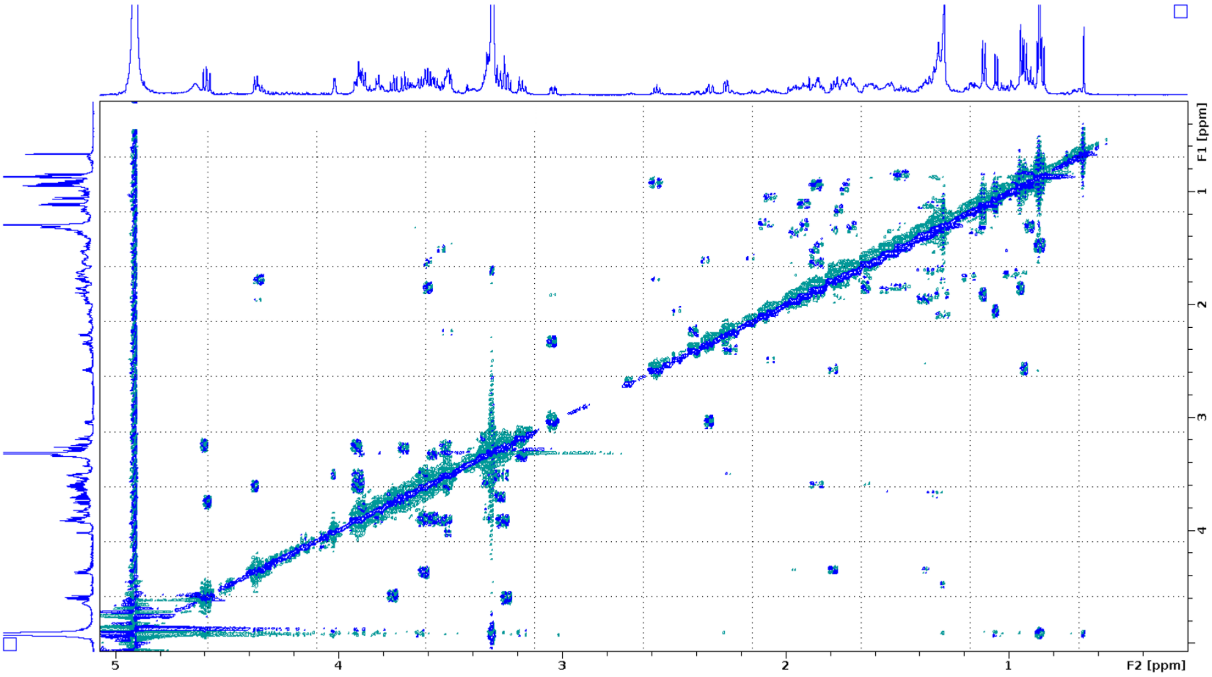

Supplementary Figure 35. 2D ROESY Spectrum of hydroxytomatine.

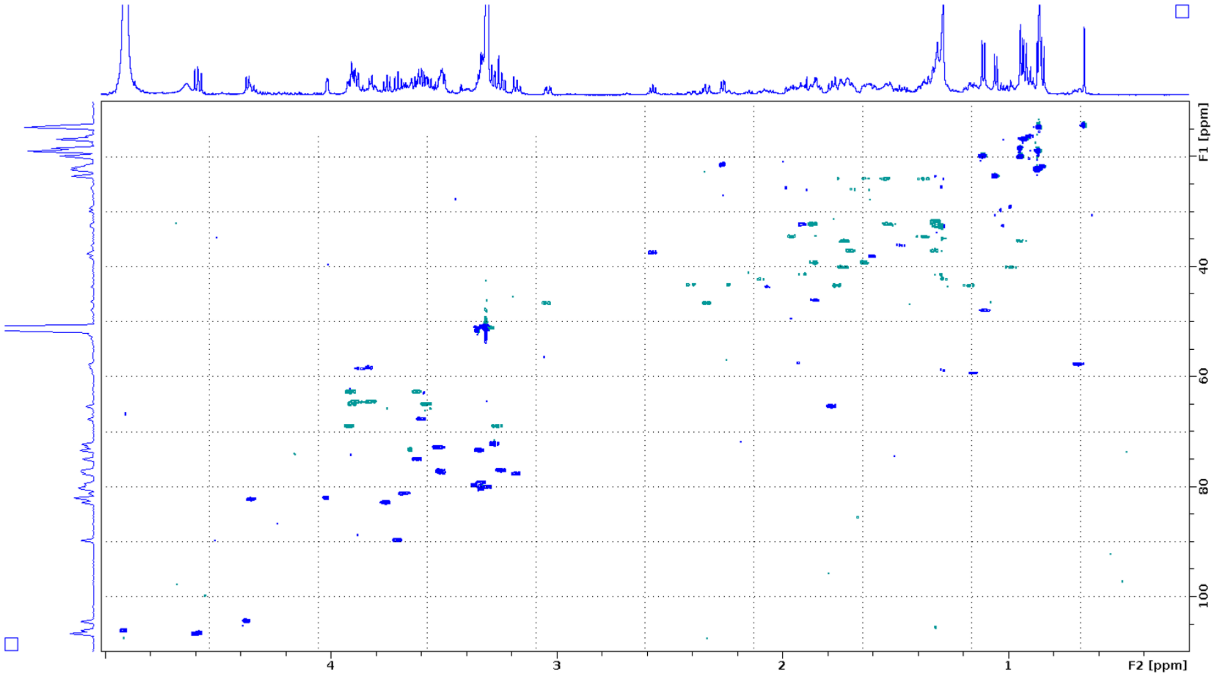

Supplementary Figure 36. 2D HSQC Spectrum of hydroxytomatine.

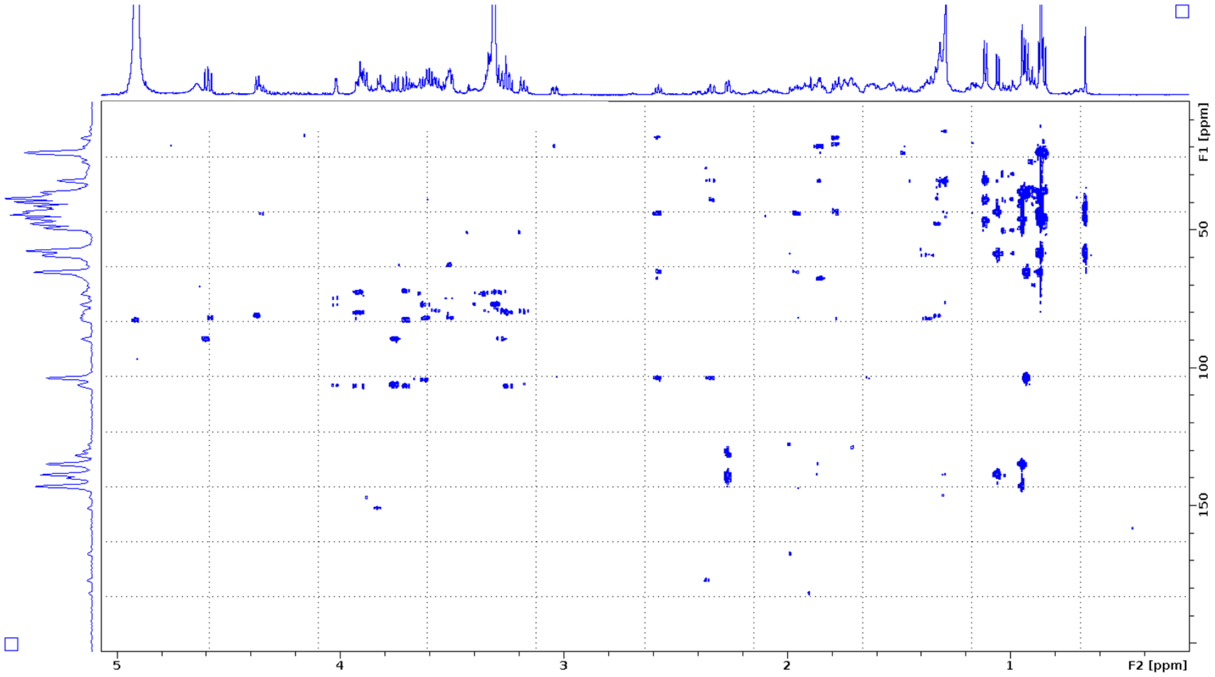

Supplementary Figure 37. 2D HMBC Spectrum of hydroxytomatine.

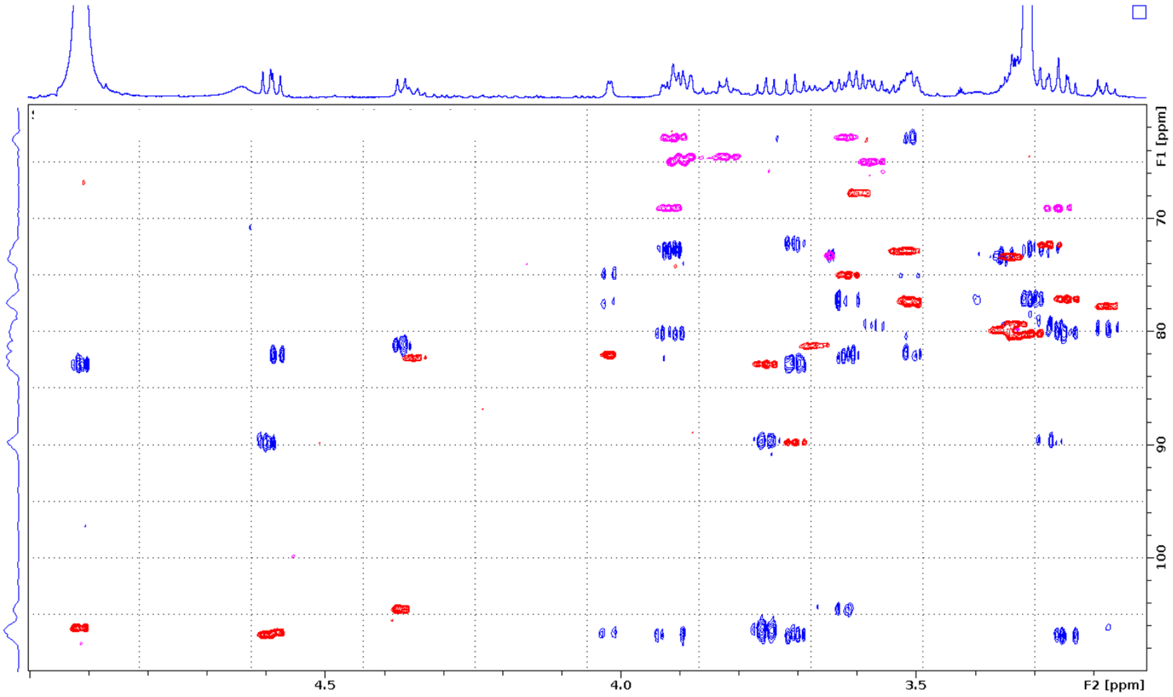

Supplementary Figure 38. Superposition of 2D HMBC over HSQC Spectrum of hydroxytomatine.

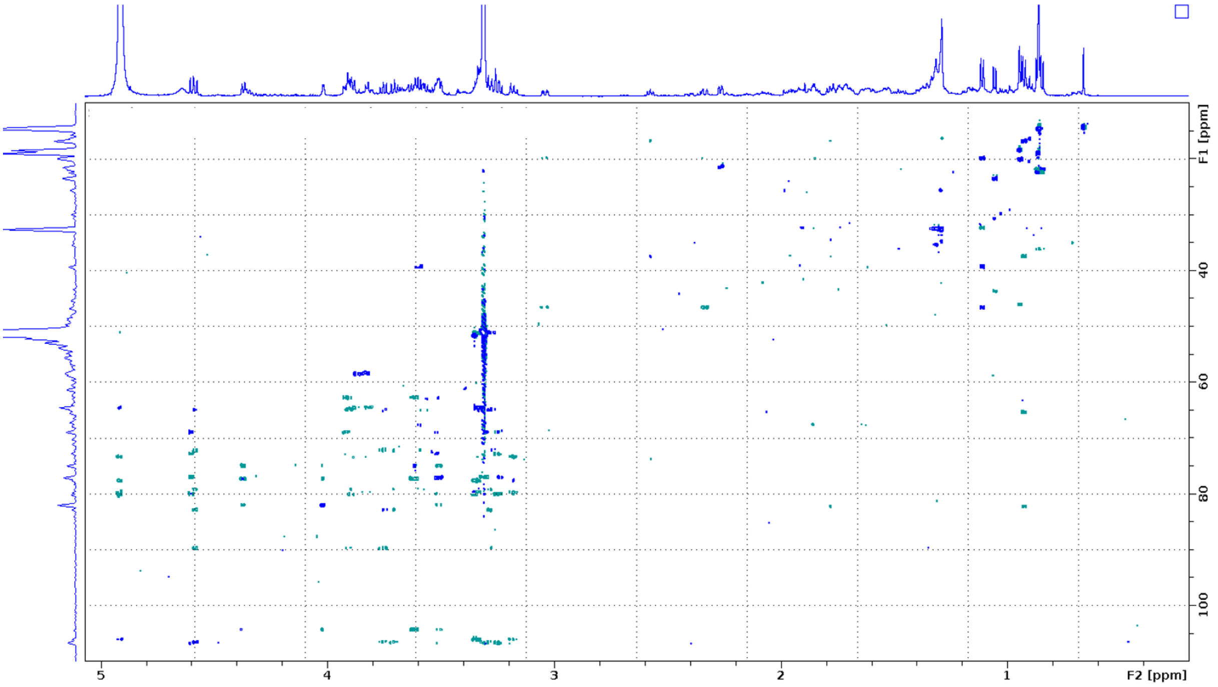

Supplementary Figure 39. 2D HSQC-DIPSI Spectrum of hydroxytomatine.

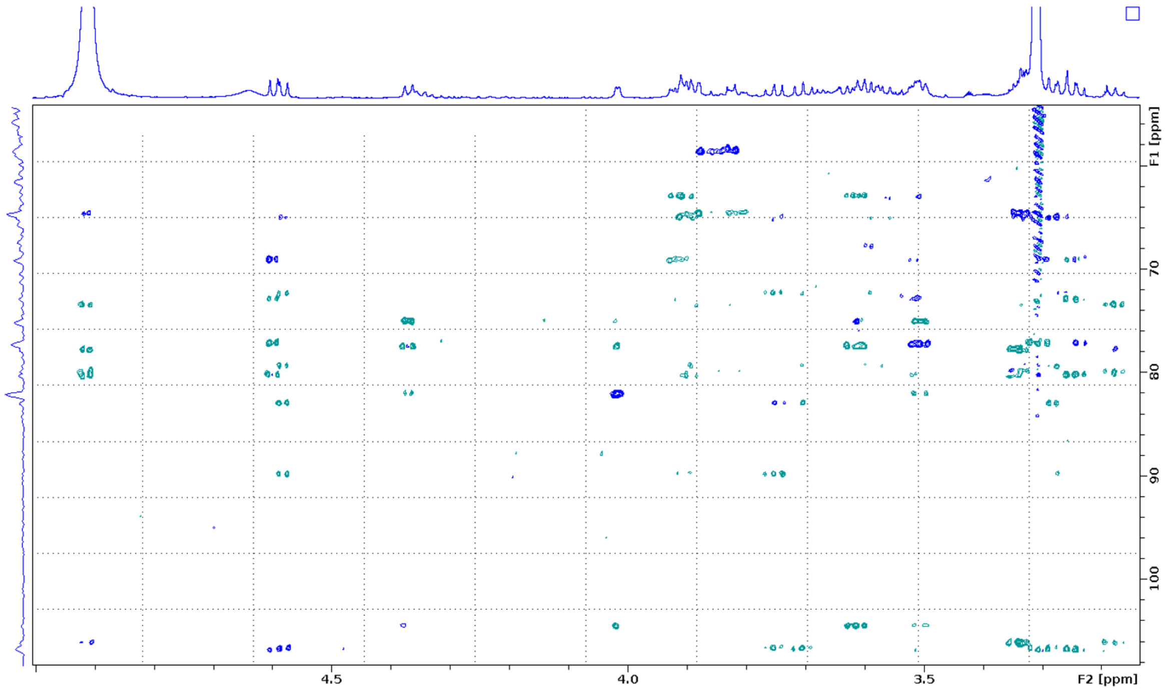

Supplementary Figure 40. 2D HSQC-DIPSI Spectrum of hydroxytomatine showing close-up view of the glycosylated region.

Supplementary Table 1. NMR spectral data for isolated hydroxytomatine compound in this study.

Position	^{13}C shift (ppm)	^{1}H shift (ppm)	HMBC Correlations
1	40.07	0.99<br>1.74 (ax)	
2	32.26	1.53 (ax)<br>1.87	
3	81.20	3.68	Gal-1
4	37.15	1.33<br>1.70 (ax)	C-3, 5, 10
5	47.85	1.10	
6	32.61	1.30	C-10
7	35.36	0.95<br>1.72 (ax)	
8	38.19	1.60	
9	57.68	0.69 (ax)	C-10,11
10	38.65		
11	24.03	1.37<br>1.54	C-9
12	43.48	1.17<br>1.76	C-18
13	44.04		
14	59.32	1.15	
15	34.62	1.37<br>1.96 (ax)	C-14, 16<br>C-13, 17
16	82.23	4.35	C-13, 17
17	65.39	1.78	C-12,16,18,21
18	19.10	0.87	C-12,13,14,17
19	14.59	0.86	C-1,5,9,10
20	37.59	2.58	C-13,17,21, 22,23
21	16.89	0.93	C-17,20,22
22	103.80		
23	67.65	3.60 (ax)	C-24
24	39.32	1.63 (ax)<br>1.86	C-23,29
25	32.35	1.92	
26	46.68	2.34<br>3.04	C-22,24,25<br>C-22,27
27	19.88	1.11	C-24, 25,26
Gal-1	104.45	4.37 (ax)	C-3
Gal-2	74.92	3.61 (ax)	Gal-1,3, 5
Gal-3	77.27	3.51 (ax)	Gal-4
Gal-4	82.14	4.02 (eq)	Gal-2,3, Glc1-1
Gal-5	77.27	3.50 (ax)	Gal-2,4,6
Gal-6	62.82	3.62<br>3.91	Gal-1<br>Gal-4

Glc1-1	106.59	4.58 (ax)	Gal-4,
Glc1-2	82.82	3.75 (ax)	Glc1-1,3 Glc2-1
Glc1-3	89.85	3.69 (ax)	Glc1-2,4, Xyl-1
Glc1-4	72.23	3.27 (ax)	Glc1-3,6
Glc1-5	79.45	3.33 (ax)	
Glc1-6	64.89	3.57<br>3.90	Glc1-5
Glc2-1	106.16	4.91 (ax)	Glc1-2
Glc2-2	77.74	3.18 (ax)	Glc2-1,3
Glc2-3	79.83	3.36 (ax)	Glc2-4
Glc2-4	73.29	3.34 (ax)	
Glc2-5	80.39	3.33 (ax)	Glc2-4
Glc2-6	64.49	3.82<br>3.89	Glc2-4
Xyl-1	106.76	4.60 (ax)	Glc1-3
Xyl-2	77.10	3.24 (ax)	Xyl-1, 3
Xyl-3	80.09	3.30 (ax)	Xyl-1, 2, 4
Xyl-4	72.74	3.52 (ax)	Xyl-3, 5
Xyl-5	69.09	3.26<br>3.92	Xyl-3, 4<br>Xyl-1, 3, 4

Note: Refer Methods section for experimental details. ax (axial) and eq (equatorial) depict protons in ring structures of aglycone and sugars.

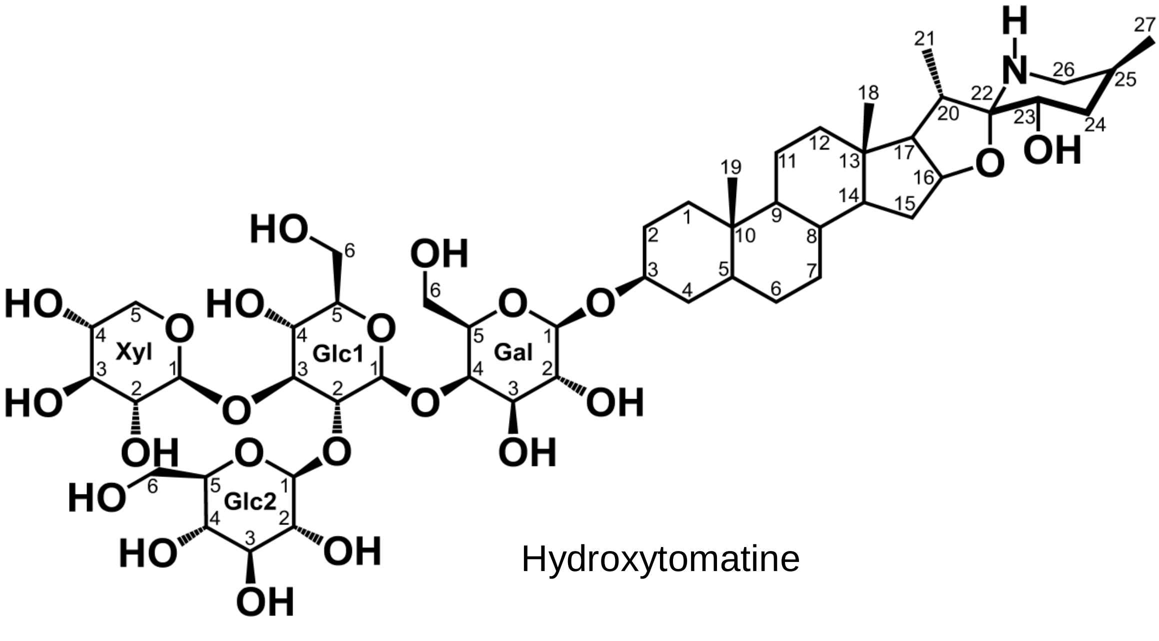

Hydroxytomatine
