# Supplementary material for: Pathways to defense metabolites and evading fruit bitterness in genus Solanum evolved through 2-oxoglutarate-dependent dioxygenases
Source: Nat Commun. 2019 Nov 14;10:5169. doi: 10.1038/s41467-019-13211-4 (PMC6856131; doi:10.1038/s41467-019-13211-4)
Supplement: Supplementary file 2 — Reporting Summary [file 41467_2019_13211_MOESM2_ESM.pdf]

nature research

Corresponding author(s): Prof. Asaph Aharoni and Prashant Sonawane

Last updated by author(s): 25 September, 2019

# Reporting Summary

Nature Research wishes to improve the reproducibility of the work that we publish. This form provides structure for consistency and transparency in reporting. For further information on Nature Research policies, see Authors & Referees and the Editorial Policy Checklist .

## Statistics

For all statistical analyses, confirm that the following items are present in the figure legend, table legend, main text, or Methods section.

| n/a | Confirmed | |
|---|---|---|
| ☐ | ☒ | The exact sample size (*n*) for each experimental group/condition, given as a discrete number and unit of measurement |
| ☐ | ☒ | A statement on whether measurements were taken from distinct samples or whether the same sample was measured repeatedly |
| ☐ | ☒ | The statistical test(s) used AND whether they are one- or two-sided<br>*Only common tests should be described solely by name; describe more complex techniques in the Methods section.* |
| ☒ | ☐ | A description of all covariates tested |
| ☒ | ☐ | A description of any assumptions or corrections, such as tests of normality and adjustment for multiple comparisons |
| ☐ | ☒ | A full description of the statistical parameters including central tendency (e.g. means) or other basic estimates (e.g. regression coefficient) AND variation (e.g. standard deviation) or associated estimates of uncertainty (e.g. confidence intervals) |
| ☒ | ☐ | For null hypothesis testing, the test statistic (e.g. *F*, *t*, *r*) with confidence intervals, effect sizes, degrees of freedom and *P* value noted<br>*Give P values as exact values whenever suitable.* |
| ☒ | ☐ | For Bayesian analysis, information on the choice of priors and Markov chain Monte Carlo settings |
| ☒ | ☐ | For hierarchical and complex designs, identification of the appropriate level for tests and full reporting of outcomes |
| ☒ | ☐ | Estimates of effect sizes (e.g. Cohen's *d*, Pearson's *r*), indicating how they were calculated |

*Our web collection on statistics for biologists contains articles on many of the points above.*

## Software and code

Policy information about availability of computer code

| | |
|---|---|
| Data collection | Not applicable for our study |
| Data analysis | Microsoft Excel 2016 and GraphPad Prism 7 softwares were used for statistical analysis . |

For manuscripts utilizing custom algorithms or software that are central to the research but not yet described in published literature, software must be made available to editors/reviewers. We strongly encourage code deposition in a community repository (e.g. GitHub). See the Nature Research guidelines for submitting code & software for further information.

## Data

Policy information about availability of data

All manuscripts must include a data availability statement. This statement should provide the following information, where applicable:

- Accession codes, unique identifiers, or web links for publicly available datasets
- A list of figures that have associated raw data
- A description of any restrictions on data availability

Data supporting the findings of this work are available within the paper and its Supplementary Information files. A reporting summary for this Article is available as a Supplementary Information file. The datasets generated and analyzed during the current study are available from the corresponding author upon request. The source data underlying Figures 2c-d, 3a-b, 4a and Supplementary Figs 2-6 (panel b), 15a-d, 16a-b, 18a-c, and 22a-d are provided as a Source Data File. Publicly available tomato different tissues RNA-seq data and ILs RNA-seq data used in our manuscript was retrieved from NCBI Sequence Read Archive with BioProject ID PRJNA307656 and DRYAD repository (http://dx.doi.org/10.5061/dryad.rm5v5) respectively.

# Field-specific reporting

Please select the one below that is the best fit for your research. If you are not sure, read the appropriate sections before making your selection.

- [x] Life sciences
- [ ] Behavioural & social sciences
- [ ] Ecological, evolutionary & environmental sciences

For a reference copy of the document with all sections, see nature.com/documents/nr-reporting-summary-flat.pdf

# Life sciences study design

All studies must disclose on these points even when the disclosure is negative.

| | |
|---|---|
| Sample size | Statistical based sample size calculation was not applicable to our study. Details of technical/biological replicates related to various experiments are provided in the manuscript, wherever necessary. |
| Data exclusions | No data was excluded in this study. |
| Replication | Details of technical/biological replicates used in various experiments are provided in methods section as well as in Main Figures and Supplementary Figures legends, wherever necessary. |
| Randomization | Not applicable for our study. |
| Blinding | Blinding was not relevant for our study. |

# Reporting for specific materials, systems and methods

We require information from authors about some types of materials, experimental systems and methods used in many studies. Here, indicate whether each material, system or method listed is relevant to your study. If you are not sure if a list item applies to your research, read the appropriate section before selecting a response.

## Materials & experimental systems

| n/a | Involved in the study |
|---|---|
| [x] | [ ] Antibodies |
| [x] | [ ] Eukaryotic cell lines |
| [x] | [ ] Palaeontology |
| [x] | [ ] Animals and other organisms |
| [x] | [ ] Human research participants |
| [x] | [ ] Clinical data |

## Methods

| n/a | Involved in the study |
|---|---|
| [x] | [ ] ChIP-seq |
| [x] | [ ] Flow cytometry |
| [x] | [ ] MRI-based neuroimaging |
