# Supplementary material for: Pathways to defense metabolites and evading fruit bitterness in genus Solanum evolved through 2-oxoglutarate-dependent dioxygenases
Source: Nat Commun. 2019 Nov 14;10:5169. doi: 10.1038/s41467-019-13211-4 (PMC6856131; doi:10.1038/s41467-019-13211-4)
Supplement: Supplementary file 3 — Description of Additional Supplementary Files [file 41467_2019_13211_MOESM3_ESM.docx]

**Description of Additional Supplementary Files**

File name: Supplementary Data 1

Description: Level of seven SGAs across tomato Backcross inbred lines (BILs) and Introgression lines (ILs) population. Relative peak area is presented for each SGA. Leaf-dip method was used for rapid screening of SGAs in leaf tissues, and further SGA analysis was performed using LC-MS. More experimental details are provided in Methods section.

File name: Supplementary Data 2

Description: List of genes present in chromosome 2 region of IL2-1 line linked to hydroxytomatine and acetoxytomatine SGA content. The SGA content in leaf tissues of BILs and ILs population was determined by leaf-dipping method and further analysis by UPLC-qTOF-MS. 2-ODD genes are marked in red.

File name: Supplementary Data 3

Description: 2-ODD GAME proteins (GAME31, GAME32, GAME32-like and 2-ODD34) and their homologous sequences used in construction of phylogenetic analysis.

File name: Supplementary Data 4

Description: Oligonucleotides used in this study.
