# Supplementary material for: Pathways to defense metabolites and evading fruit bitterness in genus Solanum evolved through 2-oxoglutarate-dependent dioxygenases
Source: Nat Commun. 2019 Nov 14;10:5169. doi: 10.1038/s41467-019-13211-4 (PMC6856131; doi:10.1038/s41467-019-13211-4)
Supplement: Supplementary file 4 — Supplementary Data 1–4 [file 41467_2019_13211_MOESM4_ESM.zip › Data 3.docx]

**>Sl 2-ODD-1**

MASTKVKIPTIDFSNEELKPNTPLWESTKVQLFEAFQEYGCIEAIYGENPNEIREGIFDIEKKIFEFPLE

TKMKNHSEIPLHIGYIGQIPHLPSYESLCIPNFLAPQSVENFANIFWPHGNPEFCNLVKSYANSLLKLDE

MIKRMILENLGLEKHINELLDNFVLFRFTHYKGTLSINKDENDKYDGLGAHTDNNFLTFIAQNQVNGLQI

NKNGEWINATISPNSFVVLSGDSFKAWTNGRLHSPLHRVAMPRENDRLSLQFNTLSKPGHFIEAPKELVD

EKHPLLFKPYEMHGLFNYVASNPGTPNAFQAYCGV

**>Sp 2-ODD-1**

MASTKVKIPTIDFSNEELKPNTPLWESTKIKLFEAFQEYGCIEAIYGENPNEIREGIFDIEKKIFEFPLE

TKMKNHSEIPLHIGYIGQIPHLPSYESLCIPNFLAPQSVENFANIFWPHGNPEFCNLVKSYANSLLKLDE

MIKRMILENLGLEKHINELLDNFVLFRFTHYKGSLSINKDENDKYDGLGAHTDNNFLTFIAQNQVNGLQI

NKNGEWINASISPNSFVVLSGDSFKAWTNGRLHSPLHRVAMPRENDRLSLQFNTLSKPGHFIEAPKQLVD

EKHPLLFKPYEMHGLFNYVASNPGIPNAFQAYCGV

**>Sl 2-ODD-2**

MASTKVKIPTIDFSNEELKPNTPLWESTKIQLFEALQEYGCIEAILYDKNLNEIREGLFDFSKKLFEFPL

ETKMKNISEVQYHIGYIGQIPHLPSYESLGIPDFLAPQSVENFANIFWPHGNHEFCNLVKSYASSLLKLD

QIIKRMILENLGLEKHINELLDNFALFRFSHYKGSLSINKDENDKYDGLSAHTDNNFLTFIAQNQVNGLQ

INKNGEWINATISPNSFVVLSGDSFKAWTNGRLHSPLHRVEMPKEGDRLSLQFNTLSKPGHFIEAPKELV

DEKHPLLFKPYEMLGLLNYVASNAGTPNAFQAYCGV

**>Sp 2-ODD-2**

MASTKVKIPTIDFSNQELKPNTPLWESTKIQLFDALQEYGCIEAILYDKNLNEIREGLFDFSKKLFEFPL

ETKMKNISEVQYHIGYIGQIPHLPFYESLGIPDFLAPQSVENFANIFWPHGNPEFCNLVKSYANSLLKLD

QIIKRMILENLGLEKHINELLDNFALFRFSHYKGSLSINKDENDKYDGLGAHTDNNFLTFIAQNQVNGLQ

INKNGEWINASISPNSFIVLSCDSFKAWTNGRLHSPLHRVAMPREGDRLSLQFNTLSKPGHFIEAPKELV

DEKHPLLFKPYEMLGLLNYVASNAGTPNAFQAYCGV

**>Sl GAME31**

MASIKSVKVPTIDFSNYQELKPNTPLWESTKIQVFEALQEYGCFEAIYDKVSKEIREETFDMSKEIFEFP

LETKVKNISEKPMHGYMGMIPQLPLYESLCIPDLLNPQSLEKFSNIFWPQGNQHFCNLIKSYSNPLVELD

GMLKRMISENLGLKNHIDELLNANYFLFRFTHYKGSSIASGDENNKAAGLGGHTDGNFLTFISQNQVNGL

QINKNGEWIDVIISPNSYVVLAGDSFKAWTNGRLHSPLHRVTMSGQNDRLSIQLFSLSKPGHFIQAPKEL

VDEEHPLLFKPFEILELFKYGTTEAGYTAPPSDLFKIYCGV

**>Sp GAME31**

MASTKSVKVPTIDFSNYQDLKPNTPLWESTKIQVFEAFQEYGCFEAIYDKVPKEIREETFDMSKEIFEFP

LETKVKNISEKPMHGYMGMIPQLPLYESLCIPDLLNPKSLQNFANIFWPQGNQHFCNLVKSYSNPLVELD

EMLKRMISENLGLKNHIDELLNANYFLFRFTHYKGSSIASGDENNKAAGLGGHTDGNFLTFISQNQVNGL

QINKNGEWIDVNISSNSYVVLAGDSFKAWTNGRLHSPLHRVTMSGENDRLSIQLFSLSKPGHFIEAPKEL

VDEEHPLLFKPFEILGLFEYGTTEAGYTAPPSDLFKIYCGV

**>St GAME31**

MASTKVKIPTIDFSNLELKPNTPLWESTKVQVFEALKEYGCFEATYDKIPNEIREGIFGITKEIFQFPLE

TKVKNYSDITLHGYVGMIPHLPFYESLCIPDLLNPQNVETFANIFWPHGNPDFCNLVKAYSNPLMELDEM

LKKMILENLGLENHIDELLDINYMRFRFTHYKGSSIISGDHENNIKQDGLNGHTDGNFLTFISQNQVNGL

QINKNGEWIDVNISPNSYVVLSGDSFKAWTNGRLHSPIHKVKIFGESDRFSIQLFSFSKPGHFIKAPKEL

VDEEHPLLFKPFEMVGLSEYVTSQAGYAAPSDAFKAYCGL

**>Sm GAME31**

MGSTKSIKVPTIDFSNHQDLKPNTPQWESTKDQVFEAFQEFGCFEAIYDKVPNEIRKGMFDVSKEIFEFPLETKLKNLSDKPLHGYMGMIPNLPLYESLCIPDLLNPQSLQNFENIFWPHGNPDFCNLVKCYSNPLVELDEMLKRMILEKLGVENQIDELLDPKYVLFRFTHYKGSSPTNGDKNTKSEGLGGHTDGNFLTFIAQNQVSGLQINKNGEWIDVNISPNSFAVLSADSFKAWTNGRLHSPIHRVTMAGENDRFSIQLFSLSKPGHFIEAPKELVDEQHPLLFKPYEMLGLFKYVTSQSGYGAPGDAFKAYCGV

**>Sc GAME31 (8380-1)**

MASTKSVKVPTIDFSNLEELKPNSPLWESTKIQVFEALQEYGCFEAIYKVPNEIKDGMLGISKEIFEFPLETKLKNFSEKPMHGYMGMIPQLPLYESLCIPDLLNPQSLETFANIFWPQGNHHFCDLVNSYSNPLVELDEMLKRMISENLGLKNHIDELLNTNYFLFRFTHYKGSSIISGDGNNKTAGLGGHTDGNFLTFISQNQVNGLQINKNGEWIDVNISPNSCVVLAGDSFKAWTNGRLHSPVHRVTMAGESDRLSIQLFSLSKPGHFIEAPKELVDEEHPLLFKPFEILGLFGYASSEAGYGAPPSDVFESYCGV

**>Sc GAME31 (M6)**

MASTKSVKVPTIDFSNLEELKPNSPLWESTKIQVFEALQEYGCFEAIYKVPNEIKDGMLGISKEIFELPLETKLKNFSEKPMHGYMGMIPQLPLYESLCIPDLLNPQSLETFANIFWPQGNHHFCDLVKSYSNPLVELDEMLKRMISENLGLKNHIDELLNTNYFLFRFTHYKGSSIISGDGNNKTAGLGGHTDGNFLTFISQNQVNGLQINKNGEWIDVNISPNSCVVLAGDSFKAWTNGRLHSPVHRVTMAGESDRLSIQLFSLSKPGHFIEAPKELVDEEHPLLFKPFEILGLFGYASSEAGYGAPPSDVFKTYCGV

**>Ca 2-ODD-1**

MASTKVQIPTIEFSNIKPNSPNWESTKIQVFEALQEYGCFEAIYNNIPNEIREAMFATSKEIFEFPLETK

MKNLSKKPFHGYAGMIPDLPLFESLCIADLLNPQSVENFANIFWPHGNSDFCNLVNSYSNPLVELDAMVK

RMILEKLGLQNYIDEFLDHKYFLFRFTRYKALSTISGAENSNPAGLNGHTDGTIMTIISQNQVNGLQINK

NNEWIDVNISPNSYAVLAGDSFKAWTNGRLHSPIHRVKTTGESDRLSIQLFSLPKPGHFTEAPKELVDEE

HPLLFKPFETLELFEYLTSHAGDSAPGETLKAYCGV

**>St 2-ODD-1**

MKVPTIDFCKPELKPGTIQWDSTKSQVFQALQEYGCFEATYDKLRNETLEAMFGRSKEIFEFPLETKMKNLSKKLPFNGYIGKLPTLPLYESVCIDDLLQLEIVETFANIFWPEGNPYKVVKSYSKPLVELDEMVKRMVLESLGLQNYIDEFLDLTSFLLRLTKYNATQDEDMGNKLGIGDHTDGNFLTIISQNQVNGLQILKKNGEWIDVDISSNSFVVLSGDSFMAWTNGRLHSPLHRVTMAGENDRFSIQLFADPKTDCTIKAPKELVDEEHPLLFKPYDMLGYFEFFGTEAGREAGPNVLKAYCGV

**>Sc 2-ODD-1**

MKVPTIDFCKSELKPGTIQWDSTKSQVFQALQEYGCFEAIYDKLRNETLEAMFGRSKEIFEFPLEIKMKNLSKKLPFNGYIGKLPTLPFNVVKSYSKPLVELDEMVKRMVLESLGLQNYIDQFLDLTSFLLRLTKYKAGQDEDIGNKPGIGDHTDGNFLTIISQNQAWTNGRLHSPLHRVTMAGENDRFSIQLFADPKTDCTIKAPKELVDEEHPLLFKPYDMLGYFEFFGTEAGREAGPNILKAYCGV

**>Sc GAME32 (8380-1-1)**

MASTKVKIPTIDFCNLELKPNTPQWESIKVQVFEALKEFGCFEAIYDKVPNEIREGMFDNLKEVFDFPLSKLIEYREKPFHIYDGQVPIIELYGSVLAADLALPNSVETFANTFWSDGNPNFSNVAKSYFKQLMELNEMVTKMVLESLGLKNYIDEILNSNVFFSRFTNYKVIKGEDENKSGLPPHTDSSYLTIIKQSQNGLQVLYKNGEWIELNNTSPNSYIVLSEDAFMAWTNDSLTSAEHRVVTTGDKDRISIQLFSLPKLEYTVKAPKELVDEDHPLLFKPFNMLEYLKYTMSGDKSGTNLKDYCRL

**>Sc GAME32 (8380-1-2)**

MASTKVKIPTIDFCNLELKPNTPQWESIKVQVFEALKEFGCFEAIYDKVPNEIREGMFDNLKEVFDFPLSKLIEYREKPFHIYDGQVPIIELYGSVLAADLALPNSVETFANTFWSDGNPNFSNVAKSYFKQLMELNEMVTKMVLESLGLKNYIDEILNSNVFFSRFTNYKVIKGEDENKSGLPPHTDSSYLTIIKQSQNGLQVLYKNGEWIELNNTSPNSYIVLSEDAFMAWTNDSLTSAEHRVVTTGDKDRISIQLFSLPKLEYTVKAPKELVDEDHPLLFKPFNLLEYNKYTMSGDKYGTNLKDYCRL

**>Sc GAME32 (M6)**

MASTKVKIPTIDFCNLELKPNTPQWESIKVQVFEALKEFGCFEAIYDKVPNEIREGMFDNLKEVFDFPLSKLIEYREKPFHIYDGQVPIIELYGSVLAADLALPNSVETFANTFWSDGNPNFSNVAKSYFKQLMELNEMVTKMVLESLGLKNYIDEILNSNVFFSRFTNYKVIKGEDENKSGLPPHTDSSYLTIIKQSQNGLQVLYKNGEWIELNNTSPNSYIVLSEDAFMAWTNDSLTSAEHRVVTTGDKDRISIQLFSLPKLEYTVKAPKELVDEDHPLLFKPFNMLEYLKYTMSGDNNGVNLKDYCRL

**>Sc GAME32-like (M6)**

MASTKVKIPTIDFCNLELKPNTPQWESIKVQVFEALKEFGCFEAIYDKVPNEIREGMFDNLKEVFDFPLSKLIEYREKPFHIYDGQITSIPLYGNVSSADLVLPNSVETFANTFWSDGNPNFSNVAKSYFKQLMELNDMVTKMVLESLGLKNYIDEILNSNFFFSRFTNYKVIKGEDQYKSELPPHTDSSYLTIIKQIQNGLQVLYKNGEWIEVNNTSPNSYIVLSADAFMAWTNDSLTAAEHRVVTTGDKDRISIQLFSLPKLEYTVKAPKELVDEDHPLLFKPFNMLEYLKYTMSGDKSGTNLKDYCRL

**>St GAME32-like**

MASTKVKIPTIDFCNLELKPNTPQWESIKVQVFEALKEFGCFEAIYDKVPNEIREGMFDTLKEVFDFPLSKLIEYREKPFHIYDGQITSIPLYGSVSSADLVLPNSVETFTNTFWSDGNPNFSNVAKSYFKQLMELNDMVTKMVLESLGLKNYIDEILNSNIFFSRFTNYKVLEGEDENRSELPPHTDSSYLTIIKQIQNGLQVLYKNGEWIEVNNTSPNSYIVLSADAFMAWTNDNLTSAEHRVVTTGDKDRLSIQLFSLPKLDYTVKAPK

**>Sp 2-ODD34**

MASTKVKIPTIDFCNSELKPNTPQWESTKVQVFEALQEFGCFEAIYDKVPNEIIEGMFDNLKEVFDFPLS

KLIEYREKPFHIYDGQIPSIPLYGSVSSADLALPNSVETFSNTFWSHENPNFSNVAKSYFKQLMELNEMV

KRMVLESLGLNNYIDEFLNSNVFMSRFTNYKVIKGENENKAELPSHTDSSYLTIIKQNQNGLQVLYKNGE

WIELNHTSPNSYIVLSADVFMAWTNGRLTSAQHKVVTTGDKDRISIQLFSIPNPDYTLKAPKELVDEEHP

LMFKPFKLPEFFKYIMLGAKNGSGVKNYCGL

**>Sl 2-ODD34**

MASTKVKIPTIDFCNSELKPNTPQWESTKVQVFEALQEFGCFEAIYDKVPNEIIEGMFDNLKEVFDFPLS

KLIEYREKPFHIYDGQIPSIPLYGSVSSADLVLPNSVETFSNTFWSHGNPNFSNVAKSYFKQLMELNEMV

KRMVLESLGLNNYIDEFLNSNVFMSRFTNYKVIKGENENEAALPSHTDSTYLTIIKQNQNGLQVLYKNGE

WIELNHTSPNSYIVLSADIFMAWTNDKLTSAQHRVVPTGDKDRISIQFFSFPNPDYTLNVPKELVDEEHP

LMFKPFKLPEFNKYIMLGAKNGLGLKNYCGL

**>St 2-ODD34-2**

MASTKVTIPTIDFCDSELKPNTPQWESTKVQVFEALQEFGCFEAIYNKVPNEIREGMFDTLKEVFDFPLPKLIEYREKPFHIYDGQIPSVPLFGSVYSADLVLPNSVETFANTFWSHGNPNFSNVAKSYFKQLMELNDMVEKMVLESLGLKNYTDEFLNSNVYMSRFTNYKVIKGENENKSALPSHTDSSYLTIIKQNQNGLQAWTNDRLTSAQHRVVTTGDKDRFSVQLFSLLNPDYTVKVPKELVDEEHPLMYKPFKMPEYNKYLMLGAKNGLGVKNYCGL

**>St 2-ODD34-1**

MASTKVKIPTIDFFNSELKPNTPQWESTKVQVFEALQEFGCFEAIYNKVPNEIREGMFDTLKEVFDFPLPKLIEYREKPFHIYDGQIPCVPLFGSVYSADLVLPNSVETFANTFWSHGNPNFSNVAKSYFKQLMELNDMVEKMVLESLGLKNYIDEFLNSNVYMSRFTNYKVIKGENENKSELPSHTDSSYLTIIKQNQNGLQVLYKNGEWIELNHTSPNSYIVLSADALMAWTNDRLTSAQHRVVTTGDKDRFSVQLFSLVNPDYTLKVPKELVDEKHPLMYKPFKMPEYNKYLMLGAKNGLGVKNYCGL

**>Sc 2-ODD34 (8380-1)**

MASTNVKIPTINFCNSELKPNTPQWESTKVQVFEALQEFGCFEAIYDKVPNEIREGIFDTLKEVFDFPLSKLIEYREKPFHVYDGQIPSVPLFGSVYSADLVLPNSVETFANTFWPHGNPNFSNVAKSYFKQLMELNDMVKKMVLESLGLKNYIDEFLNSNVYMSRFTNYKVIKGENQNKSALPSHTDSSYLTIIKQNQNGLQVLYKNGEWIELNHTSPNSYIVLSADVFMAWTNDRLTSAQHRVVTTGDKDRFSIQVFSFPNPDYTVKVPQELVDEEHPLMFKPFKLPEFNKYIKLGAKNGPGLKNYCGY

**>Sc 2-ODD34 (M6)**

MASTNVKIPTINFCNSELKPNTPQWESTKVQVFEALQEFGCFEAIYNKVPNEIREGMFDTLKEVFDLPLSKLIEYREKPFHVYDGQIPSVPLFGSVYSADLVLPNSVETFANTFWSDGNPNFSNVAKSYFKQLMELNDMVKKMVLESLGLKNYIDEFLNSNVYMSRFTNYKVIKGENQNKSALPSHTDSSYLTIIKQNQNGLQVLYKNGEWIELNHTSPNSYIVLSADVFMAWTNDRLTSAQHRVVTTGDKDRFSIQVFSFPNPDYTVKVPQELVDEEHPLMFKPFKLPEFNKYIKLGAKNGPGLKNYCGY
